# Supplementary material for: Improving policy design and epidemic response using integrated models of economic choice and disease dynamics with behavioral feedback
Source: PLoS Comput Biol. 2025 Oct 3;21(10):e1013549. doi: 10.1371/journal.pcbi.1013549 (PMC12510651; doi:10.1371/journal.pcbi.1013549)
Supplement: S1 Appendix — The Supporting Information provides a high-level overview of the general FIEM framework, which integrates individual decision-making with infectious disease dynamics, along with detailed definitions of all variables used in the model. It provides formal descriptions of the decision and infection model components, explains the model parameterization process and data sources, and presents the FIEM algorithm in detail. The document also outlines the policy scenarios evaluated in the study, including labor restrictions, cash transfers, and paid sick leave. Additionally, it lists all simulation parameters, discusses key limitations and assumptions underlying the model, and suggests possible extensions. (PDF) [file pcbi.1013549.s001.pdf]

Supporting Information for: Improving policy design and epidemic response using integrated models of economic choice and disease dynamics with behavioral feedback

Hongru Du[1,2†*], Matthew V. Zahn[3,4†*], Sara L. Loo[5], Tijs W. Alleman[5], Shaun Truelove[5,6], Bryan Patenaude[5], Lauren M. Gardner[1,6], Nicholas Papageorge[3], Alison L. Hill[6,7,8]

1 Department of Civil & Systems Engineering, Johns Hopkins University, Baltimore, Maryland, United States of America
2 Department of Systems and Information Engineering, University of Virginia, Charlottesville, Virginia, United States of America
3 Department of Economics, Johns Hopkins University, Baltimore, Maryland, United States of America
4 McDonough School of Business, Georgetown University, Washington, DC, United States of America
5 Department of International Health, Johns Hopkins Bloomberg School of Public Health, Baltimore, Maryland, United States of America
6 Department of Epidemiology, Johns Hopkins Bloomberg School of Public Health, Baltimore, Maryland, United States of America
7 Department of Biomedical Engineering, Johns Hopkins University, Baltimore, Maryland, United States of America
8 Department of Ecology and Evolutionary Biology, University of Toronto, Toronto, Ontario, Canada

† These authors contributed equally to this work.
* hongrudu@virginia.edu, matthew.zahn@georgetown.edu

Supplementary Text

General framework

In this paper we present an integrated dynamic framework that captures human behavior and disease spread that we refer to as the Feedback-Informed Epidemiological Model (FIEM). This complex exercise involves fully integrating methods from two distinct fields in a reasonable, credible, and tractable way that does not unduly limit either dimension. From epidemiology, we use a risk-stratified dynamic compartmental model of disease spread (1, 2). From economics, we draw on methods rooted in dynamic utility maximization and discrete choice modeling (3–5). These components are integrated by allowing behavior at the individual-level to influence aggregate-level outcomes such as disease spread. An advantage of FIEM is that it is flexible and can readily incorporate extensions in all three of these dimensions (i.e., the model of behavior, the epidemiological disease model, and aggregate outcomes). In this section, we provide a basic description of the key model components. It is written for an audience familiar with mathematical concepts used in economics and epidemiology so that members of each discipline understand our decisions and crucially how we integrated the tools from each discipline.

We are concerned with a population of individuals exposed to an infectious disease. At each time point (period), each individual is characterized by a set of "state variables" that describe their infection status, economic well-being, and other factors that may influence health or wealth going forward. At fixed times, individuals make a behavioral decision based on calculating how each possible decision will impact their overall well-being ("utility", a function of state variables and their decision) in the current period, and predicting how it will impact their state variables—and thus utility—in future periods. These decisions may involve trade-offs—between different aspects of utility like health vs wealth, or between current and future well-being—and optimal choices may vary between individuals. For example, a person with pre-existing health conditions might see infection as more costly than a person without pre-existing health conditions and optimally choose to engage in less risky behavior to avoid getting infected. Similarly, a person with

low-socioeconomic status might see a greater downside to not working, since the amount they can consume (i.e., income not saved) if they do not work is very low. The forward-looking aspect of an individual's decision is captured by how they perceive the likelihood of infection. If they observe low infection levels, they may conclude that engaging in riskier behaviors is worthwhile. This is the channel through which disease dynamics influence choices (in the current period).

After individuals have made a behavior decision, they are aggregated into risk groups (or more generally, assigned an individualized risk-level), defined by an individual's state variables and the decisions they made for the current period. Within the model, a period is a point in time when individuals make decisions about their behavior and it can be flexibly adjusted based on the context (daily, weekly, etc.). Risk group membership determines how individuals experience different states of infection and how likely they are to transition between different states (e.g., the number of social contacts and thus risk of acquiring infection, duration of infectiousness, severity of symptoms, likelihood of knowing their infection status, or efficacy of therapy). Given the distribution of risk groups and the distribution of infection states in each, the epidemiological model predicts how the pathogen will spread among the population in a given time window, and thus determines how an individual's infection status changes from the current period to the next one. Guided by the epidemiological model, infection transmits probabilistically from infectious to susceptible individuals, and infected individuals progress through different stages of infection. This process, along with transitions for other state variables in the model, determines what state variables the individual will observe in the next period when the decision process begins again.

FIEM integrates behavior and disease spread models in two ways. First, individuals react to their own as well as population-wide infection levels—described by the epidemiological model—when making choices about what actions to take in the current period. Second, while individuals make individually-optimal decisions, these choices are aggregated across the population and used as inputs that affect how the infection evolves from one period to the next. As a result, FIEM is a complete model that captures the feedback between behavior and disease spread. These are the two features that are critical for evaluating the effects and heterogeneous burdens of counterfactual policies for infection control.

Key Components

FIEM has several key model components, which are briefly summarized below and expanded upon below in the **Model Details** section below.

The first component is the flow utility function $u(z, d, \epsilon; \Theta)$, which measures the overall well-being (utility) of an individual in the current period. 'Utility ($u(\cdot)$) is a function of observable-state variables summarized by z, each available alternative in the individual's decision making process is denoted by d, state variables unobserved to the modeler but known to the individual are in the vector ϵ and Θ is a vector of utility model parameters. Each period, individuals choose among available alternative to maximize the present discounted value of their lifetime flow utilities based on how they expect state variables to evolve in the future, given their actions in the current period.

The next component of FIEM is the set of rules governing the transitions of state variables over time, and the expectations individuals have about them, described by transition matrix $\mathbf{P}(z', \epsilon'|z, d, \epsilon; \Psi)$ with transition model parameters Ψ and primes denoting the state variables for the next time step. We break this component down into transitions involving non-infection state variables ($\mathbf{A}$) vs changes in infection status ($\mathbf{Q}$). Non-infection state variables could change due to processes such as aging, relocation, acquisition of medical conditions that impact disease risk, job loss or promotion, or emergence of new pathogen variants that have differential risk profiles. Infection status transitions include progression from being in an uninfected to infected state, or infected to recovered state. Transitions could be deterministic or stochastic, and may or may not depend on the actions of others individuals in the population. Individuals may have perfect or imperfect information about the nature of these transitions depending on the application and available data.

The innovative component of FIEM is our framework governing transitions of infection status state variables, which endogenously incorporates outcomes from the modeled decision process. The dynamics of infection are described by a risk stratified compartmental model of disease spread. Individuals acquire and transmit infection, and progress between stages of disease, according to established epidemiological principles

and pathogen-specific parameter values. The incidence of new infections depends jointly on the prevalence of susceptible and infectious individuals, and on their likelihood of contact, so individual transition probabilities are always tied to the infection status of others in the population. The risk group an individual belongs to can directly influence their probability of transiting between disease states, by way of risk-group specific parameters (e.g., vulnerability effects risk of developing severe disease; decision to work impacts number of contacts). In addition, heterogeneous contact patterns across risk groups—non random mixing—can allow for concentration of infection in certain risk groups and amplify differences in infection dynamics between groups (e.g., low SES individuals have increased risk of acquiring infection due to higher number of contacts, and the fact that those contacts are more likely to be infected). The risk group assignments of individuals, and hence the distribution of risk groups in the population, are determined by the decisions individuals make as governed by the behavioral model as well as other state variables. This creates a dynamic feedback between disease spread and behavior.

Together, the utility function and the expectations about state variable transitions between time periods form the final key object of FIEM, the value function $V(z, \epsilon; \Theta, \Psi)$. This function captures the dynamic decision problem by describing the total expected utility—the sum of the current period utility plus (discounted) expected values in future periods given possible state transitions—under the assumption that individuals will make optimal decisions in each period. The value function can be solved using Bellman's principle of optimality (6, 7) and associated numerical methods, to extract expressions for choice probabilities conditional on current state variables. These rules allow FIEM to predict behavior that arises under different policies and, when combined with data on decision making, can be used to facilitate the estimation of the utility function's parameters, which are econometically identified under modest assumptions about the distribution of unobserved state variables, the discount rate, and individual preferences (8, 9).

Scenario and Parameters

To demonstrate the capabilities of FIEM in this paper, we designed a simple scenario based on the acute phase of the COVID-19 pandemic. Our goal is to capture and evaluate two forms of vulnerability that impact decision making and disease spread: health and economic. Our approach features calibrated model parameters, informed (but not directly inferred) from the literature. We demonstrate our calibrations are reasonable as the model reproduces predictions consistent with basic theoretical premises of epidemiology and economics (e.g., if more people engage in risky behavior, more infection will happen; people work if doing so means they earn more money; etc.). In future work we can expand the model to include additional details, formally infer parameters from data, and propagate parameter estimate inference to the model's predictions, for example to test whether predicted differences between policies are statistically distinguishable from zero.

Disease spread is described by a simple SIRS model (susceptible, infectious, recovered, susceptible) model, in which individuals who are "susceptible" to disease may transition to become infected based on exposure to another infected individual, and those who are infected are "infectious" and capable of transmitting infection for some time, before progression to a "recovered" state where they are no longer infectious and have developed some immunity to re-infection, which may eventually wane, leading them to return to the susceptible state. We used an average infectious period of 7 days, an average duration of fully-protective immunity of 6 months, and a basic reproduction number (R_0) of 2.6 (average number of secondary infections produced by each infected individual before recovery) (Table A).

We focused on a single decision that represents a common choice individuals in many settings faced early on in the pandemic. Each period, individuals make a decision to work in person or to not work. If choosing to work, individuals earn income but are more likely to incur costs (monetary and non-monetary) related to infection. Susceptible individuals who work increase their number of contacts with potentially infectious individuals, and thus increase their risk of infection. Getting infected carries utility costs associated with disease symptoms or missed economic opportunities. Working incurs additional costs for infected individuals, which may capture the discomfort of working while sick or the stigma against working while infectious. The value of R_0 we report corresponds to the value when a fixed 75% workforce participation rate, reflecting the population-level average proportion working under the selected wage and baseline consumption without the influence of disease, and assuming no behavior changes in response to the disease.

We allow for two forms of individual heterogeneity in addition to infection state: an individual's socioeconomic status (SES, high vs low), and their vulnerability to the disease (vulnerable vs non-vulnerable). Labor income and consumption values are parameterized such that not working presents a greater relative trade-off for low-SES individuals than high-SES ones based on data from the Survey of Consumer Finances (10). Low-SES individuals are assumed to have more contacts even after conditioning on their decision, and are more likely to contact other low-SES individuals. Vulnerable individuals face higher utility costs of infection compared to non-vulnerable ones (e.g., could experience more severe or longer duration symptoms), but have no difference in per-exposure susceptibility to acquiring disease. We don't explicitly model working from home, but its impact is approximately captured by our parameterization, which allows high SES individuals to have fewer contacts at work and a lesser decline in consumption if abstaining from in person work. We assume that individuals have perfect information about their own current infection state, but their information about the distribution of infection states within population has a seven-day lag. Given this information, we assume individuals can accurately predict how their work decision today will influence their probability of transitioning between infection states in the next period.

Assumptions and Extensions

There are several assumptions within FIEM. Unless noted otherwise, these assumptions are common to the FIEM framework and not the specific scenarios in our analysis.

First, we assume individuals engage in dynamic utility maximization with exponential discounting (i.e., we rule out hyperbolic discounting or impatience in decision making). This assumption is testable with data on human behavior or discounting and can be easily adapted to account for these other types of behaviors if warranted. FIEM can also alter the information available to individuals about state variables and how they may evolve given their actions. Our scenario assumes individuals possess perfect information (i.e., correct beliefs) about their risk group and infection state, and that individuals know their probability of transitioning infection states from one period to the next based on the transmission probabilities from seven days earlier. This assumption is intended to capture lags reporting information about disease transmission in the population. Moreover, we require that individuals assume that these transmission rates will hold for all future periods when making their current period choices. In practice this means individuals do not learn to anticipate these updated transmission probabilities at the start of each period. Given data on beliefs or information transition, this assumption can be altered to capture information asymmetries and perception biases, which had a known impact on the response to COVID-19 (11–13). Our general framework and scenario abstract from peer effects (i.e., how choices of other individuals influence your decisions) but this could be captured as part of the flow utility function. The general framework and our scenario exercise also rule out the strategic interaction among individuals when making their behavior decisions but do capture how the aggregate levels of infection within a period can influence individual behavior. Relaxing this assumption is possible but can lead to multiple equilibria in the behavior model, which carries methodological and computational challenges. The scenario also assumes that risk-group membership directly informs the probability of contact between individuals. This linkage is key to our framework, though the specifics of these relationships can be modified according to the states modeled and the questions at hand.

Within the FIEM framework, certain assumptions are made regarding the epidemiological dynamics. Primarily, disease transmission is modeled in our scenario using a compartmental structure, meaning individuals are classified into a finite number of discrete infection states, abstracting from the reality in which pathogen load, complex immune responses, and diverse symptoms may continuously evolve in unique ways in each individual. Our particular COVID-19-inspired scenario assumes a novel disease entering a population with no prior immunity, where infection implies infectiousness, and a temporary period of perfect immune protection exists but wanes post-infection (SIRS model), leading to damped oscillations even in the absence of any behavioral feedback. However, our general framework could accommodate any sort of infectious disease. The current FIEM framework assumes that the disease transmission model can be described with a transmission matrix which at each time describes the probability an individual in state X will move to state Y by the end of the time period (i.e., a Markov process). However, the model could be extended to allow

state transitions to also depend on past states (i.e., infection history). The version of the model used in our scenario assumes that the parameters of the disease transmission model are constant over time, but this could be relaxed to allow for dynamic parameter adjustment, for example due to other interventions altering transmission rates (e.g., mask use) or duration of immunity (e.g., pathogen variant).

In our scenario we permit two margins of individual heterogeneity to inform behavior decisions and disease spread. While this makes our scenario simple, FIEM itself is general and can be extended to capture additional features. On the epidemiological side for example, incorporating additional margins like age and co-morbidities can better inform disease spread and health outcomes. Additionally, a SIR model without stratification by age is too rudimentary to adequately capture the dynamics of COVID-19, and does not capture additional complexities like human mobility, emergent pathogen variants, and vaccination which may be relevant to the disease dynamics (14–16). On the behavior side, the scenario assumes away many factors that are relevant for determining behavior in response to infection (e.g., gender, race, education, risk preferences, political or religious beliefs), omits savings, financial behavior, and interactions between individual behavior and the macroeconomy. Despite these assumptions, we see our scenario exercise as a proof of concept that illustrates how much can be accomplished with a parsimonious model that has these components implemented in a credible manner. The clear next step for future work is to expand out this framework to capture a richer setting, which FIEM is future proofed to do in many directions.

Model Details

Variables

Individuals are indexed by m, and time is measured in discrete increments with each period denoted by t.The total size of the population at time t is denoted by N_t such that $m \in \{1, \ldots, N_t\}$. This notation is flexible and can allow the total size of the population to change over time. In each period t, an individual makes a decision $d_{mt} \in \mathcal{D}$ where $\mathcal{D}$ denotes the set of possible choices. We use d^*_{mt} to denote the optimal decision an individual can make within a period. A decision is optimal if it is associated with the highest benefits—measured in terms of expected lifetime utility, where these benefits are measured by a value function—to an individual at that time.

Different individuals may have different optimal decisions within a time period and the optimal decision for individuals need not correspond to the optimal decision for the population. Certain behavioral choices can be associated with costs which are denoted by h_{mt}. These costs are specific to the behavior choice and distinct from other types of costs that may influence other factors such as income lost or infection risk. For example, models of labor supply typically include a term for the disutility of work, which captures the non-monetary costs associated with going to work (e.g., arranging childcare, time spent commuting, interacting with colleagues). The exact interpretation or use of these costs can depend on the application at hand.

State variables refer to the factors that influence the well-being or utility for an individual and may vary over time. In general, there are three types of observable state variables in FIEM. First are individual non-infection factors (e.g., age, socioeconomic status, vulnerability to a disease), denoted by the vector $k_{mt} \in K$, where K is the set of all possible vectors of non-infection state variables. The second state variable tracks an individual's infection state and is denoted by $x_{mt} \in \mathcal{X}$ and depends on the epidemiological parameterization that is applied. The third type of state variables within FIEM are population-level state variables that are denoted by the vector E_t. At a minimum, this vector aggregates the total number of people within each of the infection states in the model but could be extended to track other population-level factors that are relevant for an individual's well-being (e.g., aggregate economic output in the economy such as wages and good production, vaccine availability, hospital capacity, government policies). We use the vector $z_{mt} = \{k_{mt}, x_{mt}, E_t\}$ to summarize these observable state variables associated with an individual at a given point in time.

We additionally assume there may be state variables that are known to individuals and impact their decisions, but are unobserved to the modeler. We denote these by ϵ_{mdt}; these may be choice specific—hence the d index. Including these unobserved variables allows us to recreate the ubiquitous finding that individuals

with the same apparent state/information do not always make identical decisions.

FIEM also has outcomes and payoff variables that are related to the decisions made by individuals in the model. Individuals are mapped to risk groups based on their non-infection state variables k_{mt} and behavioral decisions d_{mt}. An individual's risk group assignment at time t is denoted by $g_{mt} \in \mathcal{G}$, where $\mathcal{G}$ is the set of all possible risk groups. The population-level state variables E_t can be further disaggregated by risk group, denoted as E_{gt}. Individuals' decisions and state variables influence their utility (see next section) through intermediate variables that are realized or calculated using the particular model structure and parameters relevant to the problem. For example, in our example we track earnings w_{mt}, as well as the subset of earnings spent that influence utility: "consumption", c_{mt}. Individuals also realize hassle costs each period h_{mt} that influence their behavior in that period.

Decision model

The utility an individual in the model derives at time t, which is a measure of "well-being", is captured by the flow utility function $u(z_{mt}, d_{mt}, \epsilon_{mdt}; \Theta)$, which depends on their observable-state variables z, the decision the individual makes denoted by d, state variables unobserved to the modeler but available to the individual ϵ, and a vector of utility parameters Θ. The exact parameterization of the flow utility function carries implications for how agents will behave by altering their trade-offs and perceptions of risk. These features can vary based on the context or scenario.

In this framework, each individual makes the decision each time period that gives the maximum expected value of their utility over time, conditional on their current state variables. The goal is to determine this optimal decision sequence—or more formally, to solve for the optimal decision in each time period given state variables—under our model for decision-dependent payoffs and state variable transitions between time periods. This dynamic optimization problem is described by the value function,

$$V(z_{mt}, \epsilon_{mdt}; \Theta, \Psi) = \max_{\{d_{m\tau} \in \mathcal{D}\}_{\tau=t}^{\infty}} \left\{ \mathbb{E}\left[\sum_{\tau=t}^{\infty} \kappa^{\tau-t} u(z_{m\tau}, d_{m\tau}, \epsilon_{dm\tau}; \Theta) | z_{mt}, d_{mt}, \epsilon_{mdt} \right] \right\}. \quad (1)$$

The value function $V(z_{mt}, \epsilon_{mdt}; \Theta, \Psi)$ represents the maximum flow utility payoffs on an infinite horizon ranging from $t \to \infty$, made by choosing the optimal set of future decisions $\{d_{m\tau}\}_{\tau=t}^{\infty}$. Discounting ($\kappa < 1$) is used to reflect individuals' preferences for more immediate payoffs ("present-discounted"). The expectations operator $\mathbb{E}$ is used to capture the expected utility under stochastic dynamics governing how the observed and unobserved state variables in future periods ($z_{m\tau}$, $\epsilon_{md\tau}$) will evolve conditional on the current period's state variables (z_{mt}, ϵ_{mdt}) and behavior decisions d_{mt}. More generally, the expectation could also be over alternative beliefs an individual holds about the likelihood of these transitions. Indicated by the conditional term "$|z_{mt}$", this equation includes the constraint that the state variables evolve from one period to another according to the transition rules of the model.

The value function encodes the solution to a dynamic discrete time optimization problem, but solving for an infinite set of future decisions is intractable. To overcome this issue, we use Bellman's principle of optimality to reformulate the problem as a recursive "Bellman equation" (6, 7). In simple terms, Bellman's principle of optimality states that if you're trying to find the optimal path to a goal, any point along the way should get you closer to your end goal most effectively. This allows us to express the solution of the value function in terms of two consecutive periods—rather than tackling the full infinite sequence at once—thereby significantly simplifying the problem,

$$V(z_{mt}, \epsilon_{mdt}; \Theta, \Psi) = \max_{d_{mt} \in \mathcal{D}} \left\{ u(z_{mt}, d_{mt}, \epsilon_{mdt}; \Theta) + \kappa \mathbb{E}[V(z_{m,t+\Delta t}, \epsilon_{md,t+\Delta t}; \Theta, \Psi) | z_{mt}, \epsilon_{mdt}, d_{mt}] \right\}. \quad (2)$$

where Δt denotes the time interval between decisions. The expected value of the value function at time $t + \Delta t$ can be rewritten as the weighted average over all future states (observed and unobserved), where each future state's contribution is weighted by its probability of occurrence upon transitioning from $t \to t + \Delta t$, as encoded in the transition matrix $\mathbf{P}$,

$$V(z_{mt}, \epsilon_{mdt}; \Theta, \Psi) = \max_{d_{mt} \in \mathcal{D}} \Big\{ u(z_{mt}, d_{mt}, \epsilon_{mdt}; \Theta) + \kappa \sum_{(z_{m,t+\Delta t}, \epsilon_{m,t+\Delta t})} \mathbf{P}(z_{m,t+\Delta t}, \epsilon_{md,t+\Delta t} | z_{mt}, \epsilon_{mdt}, d_{mt}; \Psi) V(z_{m,t+\Delta t}, \epsilon_{md,t+\Delta t}; \Theta, \Psi) \Big\}. \tag{3}$$

The value $\mathbf{P}(z_{m,t+\Delta t}, \epsilon_{m,t+\Delta t} | z_{mt}, \epsilon_{mt}, d_{mt}; \Psi)$ describes the probability that an individual ends up in the state $\{z_{m,t+\Delta t}, \epsilon_{m,t+\Delta t}\}$ at time $t + \Delta t$ conditional on being in state $\{z_{mt}, \epsilon_{mt}\}$ at time t and making the decision d_{mt}. In this work, $\mathbf{P}$ encodes the infection model that describes how individual infection state variables (x_{mt}) evolve based on the actions of individuals within the model. The transition matrix can include components describing transitions for other observed state variables, such as age, and can encode assumptions about how unobserved state variables influence transitions. The transition matrix used in the decision model and epidemiological model can differ, allowing FIEM to capture a situation where individuals base their decisions of faulty perceptions of their transition probabilities.

To make the simulation of the decision model tractable, we must avoid integrating over the unknown states. Hence, we make a series of convenient assumptions about the unobserved state variables ϵ—established in the economics literature—to permit calculation of the value function and the optimal decision (4, 8, 9, 17, 18):

- Additive separability in the flow utility function, i.e. $u(z, \epsilon, d; \Theta) = u(z, d; \Theta) + \epsilon(d)$.
- Conditionally independent in the transition model, meaning that: 1) Conditional on the current observed state and decision choice, the future state does not depend on the current unobserved variables, and 2) conditional on the future state, the future unobserved variables do not depend on the current unobserved variables. This yields: $\mathbf{P}(z_{m,t+\Delta t}, \epsilon_{m,t+\Delta t} | z_{mt}, \epsilon_{mt}, d_{mt}; \Psi) = \mathbf{p}(\epsilon_{m,t+\Delta t} | z_{m,t+\Delta t}; \Psi)$ $\mathbf{p}(z_{m,t+\Delta t} | z_{mt}, d_{mt}; \Psi)$.
- Independent and identically distributed (i.i.d.) across individuals, periods, and choices, so that $p(\epsilon_{mt} | z_{mt}; \Psi) = p(\epsilon; \Psi)$, and following a type-I extreme value distribution (standard Gumbel distribution). Note that this assumption implies conditional independence.
- Individuals do not forecast how the arguments of P will evolve in future periods when making decisions in the current period. Rather, they assume that transitions implied by the current period state variables and transition matrix will persist in future periods and they do not anticipate that this transition matrix will change based on their actions.

While methods exist to relax the assumptions regarding unobserved state variables, they significantly complicate calculations. Under these assumptions, we can define a decision specific "net-of-errors" expected value function $\bar{V}(z_{mt}, d; \Theta, \Psi)$ that excludes the decision-specific randomness from the original value function,

$$\bar{V}(z_{mt}, d; \Theta, \Psi) = u(z_{mt}, d; \Theta) + \kappa \sum_{z_{m,t+\Delta t}} \mathbf{P}(z_{m,t+\Delta t} | z_{mt}, d; \Psi) \left[\nu + \log \left(\sum_{j \in D} \exp\left(\bar{V}(z_{m,t+\Delta t}, j; \Theta, \Psi) \right) \right) \right], \tag{4}$$

Here ν is Euler's constant, which is the expected utility resulting from the Gumbel distributed unobserved states (≈ 0.57, distinct from Euler's number $e \approx 2.72$). The assumption that the unobserved states are i.i.d. samples of a type I extreme value distribution conveniently yields an expression for the probability that a particular choice d is optimal—and thus chosen—action for individual m conditional on the observed state variables z_{mt},

$$\phi(d | z_{mt}, \Theta, \Psi) = \frac{\exp(\bar{V}(z_{mt}, d; \Theta, \Psi))}{\sum_{j \in \mathcal{D}} \exp(\bar{V}(z_{mt}, j; \Theta, \Psi))} \tag{5}$$

Conceptually, the probability that a choice is made is proportional to its relative value; decisions associated with higher values are more likely to be chosen.

If the net-of-errors value function is known, then the conditional choice probabilities of Equation (5) can be used to calculate individual's decisions at any time, and thus to simulate the model (for example to make predictions under different counterfactual scenarios). They can also be used in a likelihood framework to estimate the parameters of the model. However, Equation (4) does not immediately give the net-of-errors value function, since it is a recursive functional equation. Additional steps are required to find a numerical value function that satisfies this equation, and the method we use to do this is described below in Algorithm 1.

Non-infection state variable transitions

Non-infection state variables may evolve from one period to the next. The exact nature of these transitions is application specific. For example, if the scenario calls for tracking an individual's age across periods, the transition for this state variable may be deterministic. These transitions are allowed to depend on the actions of individuals within the model. For example, in a model that allows for financial savings, the amount of savings in the next period depends on how the stock of savings in the current period changed (i.e., all new income is consumed, some new income is saved, savings are used to smooth consumption, etc.).

To formally capture these transitions, recall $\mathbf{A}$ denotes the transition matrix for the non-infection state variables k, and is a constituent of $\mathbf{P}$. The probability that an individual in state k_{mt} at time t transitions to state $k_{m,t+\Delta t}$ by time $t + \Delta t$ is given by $\mathbf{A}(k_{m,t+\Delta t}|k_{mt}, d_{mt}; \Psi)$, referred to as $\mathbf{A}_{mt}$ for brevity.

Infection model

The dynamic epidemic model consists of a discrete set of disease states, $\mathcal{X}$ and a transition matrix $\mathbf{Q}$, whose elements specify the probability that an individual in infection state x_{mt} at time t transitions to infection state $x_{m,t+\Delta t}$ by time $t + \Delta t$, formally denoted $\mathbf{Q}(x_{m,t+\Delta t}|x_{mt}, g_{mt}, E_t; \Psi)$, or abbreviated as $\mathbf{Q}_{mt}$. The variable g_{mt} is the risk group to which an individual is assigned, and E_t represents the aggregate number of individuals in each infection state and includes risk group combinations across the whole population (E_{gt}).

The transitions between infection states are indirectly related to the decision choices d and non-infection state variables k resulting from the aforementioned decision model through assignment into risk groups $g \in \mathcal{G}$. Thus conditional on individual m's risk group at time t, g_{mt}, the model transition matrix is independent of d_{mt} and k_{mt}, and only depends on aggregated risk-group level distributions of infection states. All individuals in the same risk group have the same transition matrix, so $\mathbf{Q}_{mt} = \mathbf{Q}_{gt}$ for $m \in g$. Risk group assignments don't change as a part of the infection dynamics encoded in this model, and the sum of individuals in each risk group must be constant after these transitions, until another decision occurs. The parameter vector Ψ includes all parameters for the infection model, including the way in which parameters may (or may not) be impacted by risk group membership. The infection model is a component of the overall transition matrix used in the decision model ($\mathbf{P}$).

The epidemiological model is encoded in discrete time, with time intervals marked by the times at which we allow behavioral decisions to be updated. The time step Δt can be arbitrarily small, and the rules used to generate the transition matrices $\mathbf{Q}_{gt}$ could themselves be a continuous model between t and Δt (for example a system of ordinary differential equations), or a discrete model with much smaller time steps than those used in the decision model.

FIEM allows for any disease model wherein the effect of decisions on disease dynamics could be translated into decision-dependent risk-group membership. In theory, the number of risk groups could be as large as the population itself, although this would dramatically increase the computational cost of the model. The rates encoded in the transmission matrix typically describe a combination of reactions that depend only on model parameters, and thus represent constant per capita rates (e.g., the rate of progressing to more advanced stages of infection typically does not depend on the prevalence of infection in the population), and and other reactions wherein the per capita transition rate depends (typically linearly) on the number of individuals in another state (e.g., in typical infection models, the rate at which individuals transition from the susceptible

to infected state depends on the proportion of the population that is already infected, since they are the ones from whom transmission could occur).

Scenario

We designed a simple scenario based on the early part of the COVID-19 pandemic, where infection spreads in a previously unexposed population, conferring temporary immunity to reinfection, and individuals can make the decision to abstain from in-person work, reducing their contacts and risk of infection but losing income.

We track N individuals stratified into one of three infection states—susceptible to infection but currently uninfected (s), infected and infectious (capable of transmitting to others, i), and recovered and immune to re-infection (r). Thus, the set of infection states is $\mathcal{X} = \{s, i, r\}$ and individual m's infection state variable at a given time t, denoted by x_{mt}, takes one of these values. Individuals who are infected sustain a utility cost θ_x, which represents an expectation over all possible outcomes of disease (ranging from asymptomatic infection to death). We assume that individuals have perfect information about their infection-status and that they know the aggregate distribution of infection states with a lag $l = 7$ days when making decisions for the current period.

We consider a heterogeneous population to capture and evaluate two forms of vulnerability: health and economic. Individuals are divided into two levels of socioeconomic status (SES), and two levels of vulnerability to infectious disease, both of which we assume do not change over time. No other individual-level non-infection-status state variables are tracked. Formally,

$$k_m = \{VUL_m, SES_m\} \tag{6}$$

$$VUL_m = \begin{cases} 0 & \text{Non-vulnerable} \\ 1 & \text{Vulnerable} \end{cases} \qquad SES_m = \begin{cases} 0 & \text{High-SES} \\ 1 & \text{Low-SES} \end{cases} \tag{7}$$

Individuals with vulnerability sustain an additional utility cost of infection, which multiples the cost by a factor θ_v, representing their higher risk of developing more severe forms of infection.

Each period an individual makes a decision d_{mt} to work or not (we abstract from working at home):

$$d_{mt} = \begin{cases} 0 & \text{Not work} \\ 1 & \text{Work} \end{cases} \tag{8}$$

so the set of possible decisions is $\mathcal{D} = \{0, 1\}$.

Individuals that choose to work earn a wage (w_{mt}) based on whether they are low or high SES. The amount an individual consumes, c_{mt}, depends on their earnings in that period. If they work, we assume they consume their entire wages, while if they don't work, they consume a lower "baseline" amount, which is also based on their SES status and strictly lower than their consumption if they had worked ($\bar{c}_L$ or $\bar{c}_H$ for low and high SES, respectively). Wages and consumption are formally defined as:

$$w_{mt} = \begin{cases} w_H & SES_m = 0 \\ w_L & SES_m = 1 \end{cases} \qquad c_{mt} = \begin{cases} w_{mt} & \text{if } d_{mt} = 1 \\ \bar{c}_H & \text{if } d_{mt} = 0 \;\&\; SES_m = 0 \\ \bar{c}_L & \text{if } d_{mt} = 0 \;\&\; SES_m = 1. \end{cases} \tag{9}$$

We assume there are non-monetary costs associated with working (e.g., arranging childcare, transportation, dislike of work), which we refer to as "hassle costs" and denoted by the product $\theta_h h_{mt}$. We assume that these hassle costs follow a log normal distribution by allowing $\log h_{mt} \sim \mathcal{N}(0, \sigma_h^2)$. Using a distribution instead of a fixed value adds a source of inter-individual variation to the model, which while abstracting from the many sources of variation in reality, allows us to avoid all similar individuals from making the same decision under the same conditions, while still tracking a minimum number of state variables and parameters. If an individual decides to work while infected, hassle costs increase by p_c, which could represent

the discomfort of working while experiencing symptomatic disease, or stigma associated with working while visibly infectious, for example.

Under this model, an individual's flow utility function at time t is specified as:

$$u_{mt}(z_{mt}, d_{mt}, \Theta) = \log c_{mt}(d_{mt}) - (d_{mt} + i_{mt} p_c)\theta_h h_{mt} + i_{mt}\theta_x(1 + VUL_m \theta_v), \tag{10}$$

where i_{mt} is an indicator variable with value 1 if an individual is in the infected state and value 0 if they are not. We use $\log c_{mt}(d_{mt})$ to capture consumer risk aversion to lost consumption.

Given this parameterization, the total number of risk groups is eight (vulnerability vs non-vulnerable, low- vs high-SES, and working vs not working). Since we assume that SES and vulnerability are fixed over time, the component of the state transition matrix tracking non-infection variables ($\mathbf{A}$, is an identity matrix), and the overall transition matrix $\mathbf{P}$ is therefore defined by the infection model $\mathbf{Q}$. We further note that, as described above, all individuals in the same risk group have the same transition matrix, thus we have $\mathbf{Q}_{mt} = \mathbf{Q}_{gt}$. We describe transitions in our infection model by risk group, resulting in eight 3×3 transition matrices $\mathbf{P}_{gt}$.

We describe infection dynamics using a continuous-time risk-stratified SIRS (susceptible-infectious-recovered-susceptible) model. Susceptible individuals in risk group g can acquire infection from infected individuals in any risk group, at a rate (often called the "force of infection") that depends on their propensity to make contacts with individuals in corresponding risk group g_2 ($\mathbb{C}_{g,g_2}$) and the per-contact rate of disease transmission (β). Infected individuals immediately become infectious, recover from infection and lose the ability to transmit at a rate γ_g, leading to an average duration of infection of $1/\gamma_g$. Recovered individuals are immune to re-infection, but lose immunity and become susceptible again with a waning rate α_g (where $1/\alpha_g$ is the average duration of protection). The model is defined by a set of possible transitions that individuals can undergo to move from one state to another, along with their corresponding rates (probabilities per time).

Event	Transition	Rate
Infection	$s_g \longrightarrow i_g$	$\beta \sum_{g_2 \in G} \mathbb{C}_{g,g_2} I_{g_2}(t)/N$
Recovery	$i_g \longrightarrow r_g$	γ_g
Loss of immunity	$r_g \longrightarrow s_g$	α_g

(11)

The expected value of number of individuals in each state over time under this model can be expressed as the following system of ordinary differential equations,

$$\begin{aligned} \frac{dS_g}{dt} &= -\beta \sum_{g_2 \in G} \mathbb{C}_{g,g_2} S_g(t) I_{g_2}(t)/N + \alpha_g R_g(t), \\ \frac{dI_g}{dt} &= \beta \sum_{g_2 \in G} \mathbb{C}_{g,g_2} S_g(t) I_{g_2}(t)/N - \gamma_g I_g(t), \\ \frac{dR_g}{dt} &= \gamma_g I_g(t) - \alpha_g R_g(t), \end{aligned} \tag{12}$$

where $S_g(t)$, $I_g(t)$, and $R_g(t)$ represent the total number of susceptible, infected, and recovered individuals for each risk group at time t, while N represents the total population, computed as $N = \sum_{g \in \mathcal{G}} S_g(t) + I_g(t) + R_g(t)$. Risk-group membership is defined during the decision phase of the model and is fixed for each time period for which the infection model is simulated, so the total number of individuals in each risk group cannot change in the infection model.

This risk-group-level infection model is used to construct the transition matrix $\mathbf{P}_{gt}$ describing the probability that any particular individual in risk group g and infection state x_t at time t transitions to state $x_{t+\Delta t}$ during the current time period. The model is forward simulated between time t and $t + \Delta t$, and the probability of transition is taken as the fraction of individuals who transitioned. For example,

$$
\begin{aligned}
P_{gt}(x_{t+\Delta t} = r | x_t = s) &= 0 \\
P_{gt}(x_{t+\Delta t} = r | x_t = i) &= \frac{\mathcal{T}(I_g \to R_g)(t, t+\Delta t)}{I_g(t)} \\
P_{gt}(x_{t+\Delta t} = i | x_t = i) &= 1 - P_{gt}(x_{t+\Delta t} = r | x_t = i)
\end{aligned}
\tag{13}
$$

where $\mathcal{T}(I_g \to R_g)(t, t+\Delta t)$ denotes the number of individuals transitioning from infectious to recovered in risk group g between time t and $t + \Delta t$. Details of the numerical method used for simulating the model to future periods are provided in the "**Algorithm**" section.

Our infection model describes homogeneous mixing within each risk group and the option for heterogeneous mixing between different risk groups. Our model can incorporate heterogeneities that lead one group to have more total contacts than another group, as well as those that lead to preferential (i.e., assortative) mixing between certain groups. The way in which the decision to work—via risk group membership—influences disease risk is through the form of the contact matrix $\mathbb{C}$. We assume there is a baseline number of contacts each individual in the population would have if the entire population was high-SES and non-working C_0, which would result in a baseline probability of contacting any other individual in the population of C_0/N. For heterogeneous populations, we allow contact propensities to be group-dependent. For individuals who work, we let the propensity to contact other working individuals be increased by a factor σ_{work}. Low-SES individuals have a σ_{SES} increase in the probability of contact with others with low SES. We assume there is some degree of preferential mixing by vulnerability and risk group—regardless of the decision to work—so that the propensity for individuals to contact risk groups with the same vulnerability and SES is increased by σ_1. Mathematically, the contact propensities are,

$$
\begin{aligned}
\mathbb{C}_{g,g2} =& \sigma_{g,g2} C_0, \\
\sigma_{g,g2} =& \begin{cases}
\sigma_1 & \text{if } \{VUL, SES\}_g = \{VUL, SES\}_{g2} \\
\sigma_{\text{work}} & \text{if } d_g = d_{g2} = 1 \\
\sigma_{\text{SES}} & \text{if } SES_g = SES_{g2} = 1 \\
\sigma_{\text{work}}\sigma_{\text{SES}} & \text{if } \{d, SES\}_g = \{d, SES\}_{g2} = \{1,1\} \\
\sigma_1\sigma_{\text{work}} & \text{if } d_g = d_{g2} = 1 \text{ and } \{VUL, SES\}_g = \{VUL, SES\}_{g2} \\
\sigma_1\sigma_{\text{SES}} & \text{if } SES_g = SES_{g2} = 1 \text{ and } \{d, VUL\}_g = \{d, VUL\}_{g2} \\
\sigma_1\sigma_{\text{work}}\sigma_{\text{SES}} & \text{if } \{d, SES\}_g = \{d, SES\}_{g2} = \{1,1\} \text{ and } VUL_g = VUL_{g2} \\
1 & \text{Otherwise.}
\end{cases}
\end{aligned}
\tag{14}
$$

Or, written as a matrix, $\sigma_{g,g2} =$

$$
\begin{array}{c|cccccccc}
 & g_{d_0,v_0,s_0} & g_{d_0,v_0,s_1} & g_{d_0,v_1,s_0} & g_{d_0,v_1,s_1} & g_{d_1,v_0,s_0} & g_{d_1,v_0,s_1} & g_{d_1,v_1,s_0} & g_{d_1,v_1,s_1} \\
g_{d_0,v_0,s_0} & \sigma_1 & 1 & 1 & 1 & \sigma_1 & 1 & 1 & 1 \\
g_{d_0,v_0,s_1} & 1 & \sigma_1\sigma_{\text{SES}} & 1 & \sigma_{\text{SES}} & 1 & \sigma_1\sigma_{\text{SES}} & 1 & \sigma_{\text{SES}} \\
g_{d_0,v_1,s_0} & 1 & 1 & \sigma_1 & 1 & 1 & 1 & \sigma_1 & 1 \\
g_{d_0,v_1,s_1} & 1 & \sigma_{\text{SES}} & 1 & \sigma_1\sigma_{\text{SES}} & 1 & \sigma_{\text{SES}} & 1 & \sigma_1\sigma_{\text{SES}} \\
g_{d_1,v_0,s_0} & \sigma_1 & 1 & 1 & 1 & \sigma_1\sigma_{\text{work}} & \sigma_{\text{work}} & \sigma_{\text{work}} & \sigma_{\text{work}} \\
g_{d_1,v_0,s_1} & 1 & \sigma_1\sigma_{\text{SES}} & 1 & \sigma_{\text{SES}} & \sigma_{\text{work}} & \sigma_1\sigma_{\text{work}}\sigma_{\text{SES}} & \sigma_{\text{work}} & \sigma_{\text{work}}\sigma_{\text{SES}} \\
g_{d_1,v_1,s_0} & 1 & 1 & \sigma_1 & 1 & \sigma_{\text{work}} & \sigma_{\text{work}} & \sigma_1\sigma_{\text{work}} & \sigma_{\text{work}} \\
g_{d_1,v_1,s_1} & 1 & \sigma_{\text{SES}} & 1 & \sigma_1\sigma_{\text{SES}} & \sigma_{\text{work}} & \sigma_{\text{work}}\sigma_{\text{SES}} & \sigma_{\text{work}} & \sigma_1\sigma_{\text{work}}\sigma_{\text{SES}}
\end{array}
$$

where the subscripts d_0 and d_1 are used to denote risk group members do not and respectively do work, v_0 and v_1 are used to denote risk group members are not or are vulnerable, and s_0 and s_1 are used to denote members are of high or low SES.

Note that our formulation of the force of infection (rate at which susceptible individuals become infected) term in Eqs (11) and (12) is intentionally somewhere between the traditional density-dependent and

frequency-dependent assumptions (19). We wanted transmission to be invariant to the total population size N, but to react to the distribution of individuals across risk groups (N_g), since risk group size changes dynamically in our model, unlike commonly used age- and spatial structures in infectious disease models which are more rigid. For example, when fewer individuals choose to go to work, the remaining working individuals contact fewer other individuals in their workplace. Hence, our formulation retains the relative probability of contacts between risk groups, but not the absolute number of contacts. The probability an individual in group g contacts someone else in group $g2$ is $c_{g,g2} = \mathbb{C}g, g2/N$ and the *number* of contacts an individual in group g has with someone in group $g2$ is $C_{g,g2} = \mathbb{C}g, g2(N_{g2}/N)$.

Parameters

Our model includes parameters that characterize the decision model (Θ) and infection model (Ψ). To showcase FIEM, we chose parameter values for the model to reasonably capture the trade-off between health and wealth that individuals faced during the early stages of the COVID-19 pandemic. Parameter values were either fixed based on values estimated in the literature, or chosen to produce realistic model output. In future work, parameter values could be more formally inferred using a combination of datasets describing infection dynamics, preventative behaviors, labor supply, etc.

Infection model parameters: The parameters of the infection model are summarized in Table A. We make the simplifying assumption that risk group membership affects *only* contact probabilities, and does not confer any differential susceptibility, duration of infectiousness, or duration of immunity (i.e., $\gamma_g = \gamma$ and $\alpha_g = \alpha$). However, this assumption can easily be relaxed to allow for additional complexity or to model different decisions in future work.

We assume an average duration of infectiousness of 7 days, based on studies estimating generation intervals, duration of viral shedding, and symptom duration, which corresponds to an average rate of recovery from infectiousness of $\gamma = 1/7 = 0.14$ days (20–23). After recovering from infection, we assume immunity that confers perfect but temporary protection against re-infection, with the average duration of protection of 6 months (rate of waning $\omega = 0.004$/day). This value was taken from two meta-analyses that measured the efficacy of protection against the Omicron variant as a function of time since prior infection with pre-Omicron variants. The rate of waning of protection against pre-Omicron variants was estimated to be significantly slower, but is less relevant due to the emergence of the Omicron variant in November 2020. Since the decay of protection is assumed to be constant in our simplified model but observed to be variable over time in these studies, we chose this waning rate to be consistent with the residual protection remaining at 7.5 months (the time between peak pre-Omicron infections in spring 2020 and peak Omicron infection in late 2020/early 2021 in many regions of the world) (24, 25).

We assume a baseline rate of contacts at any given time $C_0 = 4$, which is informed by the average effective number of contacts occurring at home relevant to respiratory disease transmission(26–28). To model variation in the propensity of contact between risk groups, we assume individuals who work have 4 times the probability of contact with other individuals who work ($\sigma_{\text{work}} = 4$), which is roughly based on observed numbers of average work contacts and our assumption that 75% of the population is working at baseline. We additionally assume individuals who are of low socioeconomic status have 1.5 times the probability of contact with other low socioeconomic status individuals ($\sigma_{\text{SES}} = 1.5$), based on numerous lines of evidence suggesting low SES status is associated with more household crowding as well as jobs that require more high-risk in-person interactions (29–34). Finally, to add an additional source of assortitivity ("homophily") to capture the many ways in which human interactions tend to include preferential mixing across many sociodemographic and behavioral characteristics (35–38), we assume individuals have 1.5 times greater probability of contact with individuals belonging to the same vulnerability and SES groups ($\sigma_1 = 1.5$). Note that because our model is based on proportional mixing and is does not include a formal network model of discrete contacts, only the product of the transmission rate β (which is calibrated to give the desired epidemic growth, $R_0 = 2.6$, based on a meta-analysis of estimates from early COVID-19 outbreaks (39)) and the contact matrix $\mathbb{C}$ influences results, not their separate values nor the exact number of contacts they imply.

Decision model parameters: The parameters of the decision model describe how an individual's utility depends on their state variables and the decision they make (Equation 10). These parameters are listed, along with descriptions, interpretations, assumed values, and data sources, in Table B.

To parameterize the US population with two SES groups of equal size, we used the median pre-tax family incomes for the 20–40% percentiles for the low SES group and those of the 60–80% for the high SES group. All data was obtained from the 2022 Survey of Consumer Finances (SCF) (40). For the low SES group this corresponded to a median annual pre-tax family income of \$35,600 per year or \$98 per day, and for the high SES group it was \$95,700 per year or \$262 per day. To estimate consumption in the absence of wages (baseline consumption, when not working), we used estimates of the family holdings of financial assets from the SCF. For low SES families we estimated savings rates of 15% (or baseline consumption values of \$15 per day), and for high SES families we estimated 25% savings (or baseline consumption of \$66 per day).

To ease the computational burden associated with having to uniquely evaluate value functions for each individual, we discretize distribution of hassle costs h into three categories:

$$\log \overline{h}_{mt} = \begin{cases} -0.38 & \log h \leq -\sigma_h \\ 0 & \log h \in (-\sigma_h, \sigma_h) \\ 0.38 & \log h \geq \sigma_h \end{cases}$$

where the value for each category is approximately the conditional mean of $\log h$ within each interval for a distribution standard deviation $\sigma_h = 0.25$. Together with the utility parameter θ_h, we set the baseline hassle cost for the average individual at \$116 per day. A one standard deviation increase results in a hassle cost of \$193 per day, while a one standard deviation decrease brings it down to \$73 per day. This range captures the variation in hassle costs across individuals, reflecting different levels of inconvenience or burden. These hassles are moderate in size and used to generate variation in choices within our stylized application. Richer models informed by data may break up these costs into different sources and allow for more heterogeneity in their values across the population.

To choose realistic values for the utility cost of infection (θ_x, θ_v), we attempted to estimate the value per statistical case of COVID-19 for the United States. The value per statistical case (VSC) extends the concept of the value per statistical life (VSL) by taking into account all possible clinical outcomes of infection, not just the chance of death. It is a measure of individuals' willingness to pay to avoid negative outcomes of disease. After reviewing literature estimates for VSC for COVID-19, we identified serious limitations in the few existing estimates (41–43, reviewed below). Conducted in early 2020, they did not rely on detailed clinical characteristics specific to COVID-19 to estimate their effects on quality of life, but instead used other diseases or injuries as proxies. They also did not attempt to include how early in the pandemic, uncertainty (and in some cases, overestimation) of the likelihood of severe outcomes as well of fear of spreading infection to loved ones influenced individuals' willingness to avoid infection. Therefore, we calibrated out simulation to be consistent with a VSC of approximately \$6,000 per day, slightly higher than, but in the same range, as existing estimates.

To match our simulation to a particular VSC, we chose values of θ_x (utility cost of infection) and θ_v (fold increase in cost of infection in vulnerable individuals) and then calculated the average compensating variation in our simulated population between being in the infected state vs. not being in the infected state (i.e., the amount of consumption that would leave an individual indifferent in utility terms between the two options). We first chose $\theta_v = 3$ to represent the realistically large difference in the risk of severe COVID-19 between groups. For example, this is consistent with a 3-fold increase in the risk of death between ages 40 and 50 (44) or in the risk of hospitalization between ages 40 and 60 (45). Then, the value of θ_x is calibrated such that the daily cost of infection in the model (compensating variation described above) matches the desired VSC. Let z denote the daily cost of infection, and $\hat{c}$ denote the average baseline daily consumption (estimated at \$40.5, computed as \$66 for high-SES individuals and \$15 for low-SES individuals). We determine θ_x by solving:

$$\log(\hat{c} + z) + \theta_x = \log(\hat{c}), \tag{15}$$

which equates post-infection utility to baseline utility adjusted by the infection cost. Substituting $\theta_x = -5$ and $\hat{c} = 40.5$, yields $z \sim \$5,912$.

Estimating value per statistical case of COVID-19: We stratified the potential outcomes of COVID-19 into five degrees of severity: Fatal (resulting in death), critical infection (requiring care in an ICU), severe infection (requiring hospitalization but not ICU-level care), mild infection (symptomatic infection not requiring hospitalization), and asymptomatic infection. We then estimated proportion of infections that resulted in each outcome. For deaths, we used the central estimate of the infection-fatality risk (IFR) reported in a large meta-analysis, for age 40 (0.068%, 44). For hospitalized infections (severe and critical), we started with an estimate of the case-hospitalization-risk (CHR) (for age group 35–44, 2.8%, 45), and then adjusted this for the proportion of infections that end up as reported cases by comparing estimated prevalence of infection to cumulative reported cases (as of late Nov 2020, an estimated 9.4% of the national population was serpositive indicating prior infection, whereas cumulative cases amounted to 4.1% of the population (46, 47). This indicated that 44% of infections had been detected nationally, assuming no sero-reversion and representativeness of cohorts used for serosurveillance). Then, we estimated the fraction of all hospitalized cases that end up in the ICU ('critical') using national clinical outcomes data from 2020 ($\approx$ 20%, 47), and assigned the rest to be 'severe'. We assumed all deaths occurred in critically-ill individuals, so subtracted the probability of death to get the probability of non-fatal critical infection (ignoring the fact that in reality, some deaths may occur in those not yet hospitalized or in critical care). Finally, we used an estimate that 40% of COVID-19 infections (pre-vaccination) are asymptomatic (48), and then assigned the remaining fraction to be mild (58.7%).

We used two different sources for estimates of the value per statistical case at each level of severity (42, 43) (Table C). The United States Department of Health and Human Services published an internal report and later a peer-reviewed study on VSC estimates for COVID-19 by Robinson et al. (41, 42). For each non-fatal outcome, VSC values were estimated based on the QALYs (Quality Adjusted Life Years) lost due to the duration and severity of the disease outcome: \$5,000 for mild infection \$11,000 for severe infection, and \$2,113,000 for critical infection (again, for age 40). For fatal infections, the associated VSC is HHS's standardized central estimate for the value of statistical life (VSL) for a 40 year old using a 2% annual future discount factor (\$13,100,000). With our outcome probabilities described above, these values result in an implied average VSC of $\approx$ \$17,000, or with an average duration of infection of seven days, $\approx$\$2,500 per day of infection (range \$1,140 to \$8,000 using their upper and lower estimates). Note that HHS's values should be considered loose approximations, as they were estimated in 2020/2021 by collecting studies that calculated QALYs lost for other diseases that had overlapping symptoms to COVID-19 (such as influenza, COPD, other causes of acute respiratory distress), many of which they admit are of limited similarity and quality, and have not been updated with specific calculations for COVID-19 (42). In particular, their large discrepancy between estimates of value for severe vs critical infection is due to their assumption that severe infection results in only around 6 weeks of post-hospitalization sequelae, whereas critical infection leads to lifelong disability—an oversimplification inconsistent with current understanding of infection. They did not specifically address asymptomatic infection—seeming to use proxies for this category that included only symptomatic conditions, so we took the liberty of adding the asymptomatic category at zero cost as a compromise.

Separately, an estimate from Kniesner and Sullivan (43) used VSC estimates taken from the U.S. Department of Transportation's estimates of effective reductions in VSL for different levels of injury severity. With their mapping, the VSCs for COVID-19 were \$10,900 for asymptomatic infection, \$32,700 for symptomatic, \$512,200 for hospitalization without critical care, \$1,144,500 for ICU care without mechanical ventilation, \$2,899,400 for ICU care with mechanical ventilation, and \$11,000,000 for death. With our outcome probabilities described above, and using CDC estimates that 50% of ICU patients require mechanical ventilation, these values result in an implied average VSC of $\approx$\$40,000 or $\approx$\$5,700 per day of infection. The authors suggest this is likely an underestimate, as prior studies on VSL conducted in the context of real or hypothetical pandemics have found that the 'dread' of infectious disease outbreaks leads individuals to associate higher value with avoiding infection compared to other causes of death (49, 50) Based on those studies, the authors

suggest VSL multipliers up to 3–5-fold may be appropriate for COVID-19, and thus the daily average VSC could be as high as \$20,000 to \$30,000. Based on the wide variation in these estimates, we chose to calibrate our simulation to be roughly consistent with a VSC of ∼\$6,000 per day, which is somewhat in the middle (logarithmically) of the range suggested by these estimates (\$1,000 to \$30,000).

Algorithm

In this section we describe how to recover the solution of the combined behavior-disease model each period. To summarize, at the beginning of a period, each individual is associated with state variables $z_{mt} = \{k_{mt},\ x_{mt},\ E_t\}$. An iterative numerical procedure (Algorithm 1), described in detail below, is used to numerically recover the net-of-errors value functions (Equation 4) for each possible combination of state variables and decisions. These are then used to calculate the conditional probabilities of making different decisions for each individual (Equation 5), which in turn allow us to construct the model's risk groups. In the intervening time period Δt until the next decision is made, infection levels evolve in a risk-group specific manner according to the infection dynamics model. The cumulative transitions in infection status during the proceeding period are used to construct the transition matrix describing expectations about changes in infection states in the future, that then feeds into the estimated value functions—and hence decisions—at the next decision update. Continually repeating this procedure of successively approximating the value functions, calculating decision probabilities, and executing changes in decision status and infection levels for the next time step allows us to simulate the paths of choices individuals make over time as their state variables evolve.

We use the nested fixed point method, a type of successive approximation introduced by (4), to recover the full solution to an individual's dynamic discrete choice problem at each time step. To understand how this method works, consider the following simple example. Suppose the decisions d and states z are binary so there are four combinations. We use a and b to index the states z, and 0 and 1 to index the decisions d. Without loss of generality, transitions between a and b are captured by the transition matrix $\mathbf{P}_{mt}(d, z; \Psi)$ (note since we are here describing decision making at the individual level, we index by m). This process produces a system of equations based on the net-of-error value functions. To slightly abuse notation, let $\bar{V}^i_{zt}(d)$ denote iteration i of the net-of-error value function for an individual with state variables z making decision d in period t:

$$\begin{aligned}
\bar{V}^{i+1}_{at}(0) &= u(0,a;\Theta) + \kappa \sum_{z_{m,t+\Delta t}} \mathbf{P}_{mt}(0,a;\Psi)\left[\nu + \log\left(\exp(\bar{V}^i_{a,t+\Delta t}(0)) + \exp(\bar{V}^i_{b,t+\Delta t}(0)) + \exp(\bar{V}^i_{a,t+\Delta t}(1)) + \exp(\bar{V}^i_{b,t+\Delta t}(1))\right)\right] \\
\bar{V}^{i+1}_{at}(1)) &= u(1,a;\Theta) + \kappa \sum_{z_{m,t+\Delta t}} \mathbf{P}_{mt}(1,a;\Psi)\left[\nu + \log\left(\exp(\bar{V}^i_{a,t+\Delta t}(0)) + \exp(\bar{V}^i_{b,t+\Delta t}(0)) + \exp(\bar{V}^i_{a,t+\Delta t}(1)) + \exp(\bar{V}^i_{b,t+\Delta t}(1))\right)\right] \\
\bar{V}^{i+1}_{bt}(0) &= u(0,b;\Theta) + \kappa \sum_{z_{m,t+\Delta t}} \mathbf{P}_{mt}(0,b;\Psi)\left[\nu + \log\left(\exp(\bar{V}^i_{a,t+\Delta t}(0)) + \exp(\bar{V}^i_{b,t+\Delta t}(0)) + \exp(\bar{V}^i_{a,t+\Delta t}(1)) + \exp(\bar{V}^i_{b,t+\Delta t}(1))\right)\right] \\
\bar{V}^{i+1}_{bt}(1) &= u(1,b;\Theta) + \kappa \sum_{z_{m,t+\Delta t}} \mathbf{P}_{mt}(1,b;\Psi)\left[\nu + \log\left(\exp(\bar{V}^i_{a,t+\Delta t}(0)) + \exp(\bar{V}^i_{b,t+\Delta t}(0)) + \exp(\bar{V}^i_{a,t+\Delta t}(1)) + \exp(\bar{V}^i_{b,t+\Delta t}(1))\right)\right]
\end{aligned} \tag{16}$$

(4) proved that under certain assumptions (i.e., $\kappa < 1$ and bounded flow utility), the true value function is a fixed point of this system of equations. Thus, given an initial guess for the values of $\bar{V}^0_{zt}(d)$, we can iterate on this system to recover the numerical values for the value functions within a pre-specified tolerance for a given utility parameter vector Θ.

The infectious disease dynamics are described by continuous rates (Equation 11) at which individuals in a given risk group transition between susceptible ($S(t)$), infected ($I(t)$), and recovered ($R(t)$) states. Although we model disease dynamics on a continuous time scale, the decision model requires a transition matrix describing infection dynamics over a discrete time period. To efficiently make this bridge in timescales while avoiding numerical errors with discretizing continuous models, we calculate updates of the infection model in smaller time steps, δt, using a simple discrete time Euler update. For the SIRS model described in Equation

(12), the transition probabilities are

$$
\begin{aligned}
p(s_{g,t+\delta t}|s_{gt}) &= \left(1 - \beta \sum_{g_2 \in G} \mathbb{C}_{g,g_2} I_{g_2}(t)/N\right) \delta t \\
p(i_{g,t+\delta t}|s_{gt}) &= \left(\beta \sum_{g_2 \in G} \mathbb{C}_{g,g_2} I_{g_2}(t)/N\right) \delta t \\
p(r_{g,t+\delta t}|s_{gt}) &= 0 \\
p(s_{g,t+\delta t}|i_{gt}) &= 0 \\
p(i_{g,t+\delta t}|i_{gt}) &= 1 - \gamma_g \delta t \\
p(r_{g,t+\delta t}|i_{gt}) &= \gamma_g \delta t \\
p(s_{g,t+\delta t}|r_{gt}) &= \alpha_g \delta t \\
p(i_{g,t+\delta t}|r_{gt}) &= 0 \\
p(r_{g,t+\delta t}|r_{gt}) &= 1 - \alpha_g \delta t
\end{aligned} \tag{17}
$$

which are the elements of the transition matrix $\mathbf{P}_{gt}$. To update the state of each individual, we randomly choose a transition to occur using a multinomial distribution with parameters given by the transition probabilities.

We conclude with brief comments on computation and tractability. As the model includes more state variables or decisions, the number of components within equation (16) increases, leading to a higher computational burden. Thus it is possible to specify a model that is so rich that solving for the fixed point of equation (16) becomes intractable. However, we do not see this as a major concern to the FIEM. The computational burden can be alleviated by using parallel computing (i.e., if your parameterization allows you to break up the net-of-error value functions into groups) or through the use of fixed-point acceleration algorithms. Additionally, computational resources are still continuously improving. Finally, an effective model should be parsimonious enough to answer the question at hand without introducing unnecessary features that can exacerbate any tractability issues.

Policy Scenarios

In the main text we use FIEM to simulate the effects of four alternative policies on disease spread, labor supply, and other individual outcomes. The motivation behind each policy is to reduce the number of contacts individuals have within a period. If individuals have fewer contacts then the infectious disease will not spread as much through the population absent the intervention. The policies differ in terms of the mechanism that is used to reduce contacts, which groups are targeted, and their fiscal costs to the government. Here we detail how these policies are implemented as part of the FIEM framework. Individuals cannot anticipate any of these policies and do not modify their behavior in the run up to their implementation.

The first policy is the labor restriction. This policy loosely resembles some of the so-called "lockdown" measures used during the COVID-19 pandemic that resulted in some businesses or agencies not being open. Under this policy, a randomly chosen share of the population is forced to remain at home and does not receive wages, while the other portion of the population can choose to work or not. In practice, this means that the portion of the population that is under the restriction does not solve the dynamic programming problem because they are only able to take a single action each period. In terms of the notion introduced previously, this policy reduces the set $\mathcal{D}$ for the randomly chosen portion of the population to a singleton (i.e., not working for the period).

The second policy is an unconditional cash transfer. This policy also resembles actions taken during the recent COVID-19 pandemic when governments provided direct cash payments to individuals. Each period, individuals receive a cash transfer from the government. This transfer is added to the income the individual receives that period based on whether they work or not (i.e., $w^*_{mt} = w_{mt} + b_{mt}$, where b_{mt} is the value of the

Algorithm 1 FIEM Solution

procedure FIEM($t, z_{mt}, \Psi, \Theta, \Delta t, \delta t, n, \text{tol} = 10^{-6}$)
  Initializing t, state variables z_{m0}, number of periods (n), decision model step size (Δt), infection model step size (δt)
  while $t < n$ **do**
    Calculate transition matrix $\mathbf{P}_{gt}(E_t; \Psi)$ using Equation (17) with time step = Δt, calculate flow utility $u(z, d; \Theta)$
    Numerically recover net-of-error value functions $\bar{V}_{zt}(d)$ using $\mathbf{P}_{gt}$, $u(\cdot)$ and guess for $\bar{V}^0_{zt}(d)$:
    Initializing $i = 0$
    while $\Upsilon > \text{tol}$ **do**
      Calculate $\bar{V}^{i+1}_{zt}(d)$ using Equation (16)
      Calculate $\Upsilon = ||\bar{V}^{i+1} - \bar{V}^i||_2$
      Update $\bar{V}_{zt}(d) = \bar{V}^{i+1}_{zt}(d)$
      $i = i + 1$
    end while
    Given $\bar{V}_{zt}(d)$ recover conditional choice probabilities $\phi(d|z_{mt}, \Theta, \Psi)$ using Equation (5)
    Randomly assign individuals their decision for the period with probabilities $\phi(d|z_{mt}, \Theta, \Psi)$
    Given decisions and non-infection state variables, construct risk groups
    Initialize j = 0
    while j < Δt **do**
      Calculate $\mathbf{P}_{gt}(E_t; \Psi)$, where time step = δt
      Randomly assign individuals their updated infection states $x_{m,t+\delta t}$ with probabilities in $\mathbf{P}_{gt}$
      j = j + δt
    end while
    $t = t + \Delta t$
  end while
end procedure

transfer provided). Since we rule out savings, these cash transfers are consumed by individuals that period. The third policy is a conditional cash transfer. It is the same as the previous policy but only individuals that choose not to work will receive the payment from the government (i.e., individual m will only receive the payment b_{mt} if $d^*_{mt} = 0$). These policies were used later on in the COVID-19 pandemic. Individuals know they will receive the government payment if they choose not to work that period.

The final policy simulates the effects of paid sick leave. While this policy was not part of the response to the COVID-19 pandemic used by most governments, it is designed based on the same core motivations. It seeks to directly target the populations that present the greatest risk of spreading the disease through the population by giving them a direct incentive to reduce contacts. This incentive arises through the same channel as the cash transfer measures by increasing the income and consumption of targeted individuals. The transfer is only available to individuals that are infected that choose not to work. Once the policy is activated, individuals are aware that they can receive this payment if they become infected and choose not to work. We do not assume there are any barriers to taking up the benefit.

Model Validation

In this section we present two sets of validation exercises. The first type of validation experiments focuses on ensuring the model of individual behavior responds in predictable ways, while the second type performs a similar assessment of the epidemiological model. The value of these exercises is that they confirm the models of individual behavior and epidemiological disease spread are designed and implemented correctly. They also develop intuition for what factors drive behavior and disease spread within each component of our framework in isolation. Establishing this baseline is important for evaluating the predictions and performance of the

integrated framework where both models feedback to each other.

Infection model validation In this section, we perform an additional set of validation exercises for the risk-stratified disease spread model. To validate the epidemiological core of our model, we first disabled the behavioral feedback component. In this configuration, the model reduces to a conventional stratified SIRS framework. In the special case of a single risk group, analytic results exist for the final peak size, peak time, and R_0, as well as threshold conditions for outbreak versus extinction (i.e., $R_0 < 0$). We confirmed that our implementation reproduced these expected results across a range of parameter values (see revised Table S4). Additionally, we compared the time series output of our model to results from previously developed code used in related SIRS modeling applications without behavioral feedback. This cross-validation demonstrated close agreement (see example visualization in Fig A).

Then, we explore the behavior of the disease dynamics when individuals' decisions do not change in response to the overall dynamics. We consider the disease dynamics when risk group membership stays constant (Fig B). This is equivalent to a standard compartmental disease model with some introduced level of heterogeneity in contact patterns. First, we note that when no individuals work, the disease is eliminated, as there is insufficient (or no) contact required to sustain an epidemic. Conversely, when all of the population work, the epidemic is quick and sharp. When an intermediate proportion of individuals work, e.g. half the population goes to work, the peak of the epidemic is significantly reduced. The decrease in the population working corresponds to a decrease in contact propensity, thus lowering the basic reproductive number.

We also consider the effect of different proportions of other static state variables (vulnerability and socioeconomic status). All other states constant (socioeconomic status and working state), vulnerability does not affect disease dynamics when there is no dynamic decision making; this is an expected result when vulnerability only feeds back to decision making, and it is the decision to work that inevitably drives disease dynamics.

Similarly, when the decision to work is constant, higher ratios of low- to high-socioeconomic status also increases the peak size and initial exponential growth rate of the epidemic. We note an important behavior, in that these dynamics hold true as a result of our parametrization of our contact matrix, where individuals have a higher probability of contact with those in their own risk group, workers interact with other workers more often, and low socioeconomic status individuals also interact at a higher propensity to contact other low-socioeconomic status individuals.

We also note that due to the choice of our SIRS compartmental structure, disease equilibria can result in oscillations, as the population cycles between susceptibility and recovery.

Behavioral model validation To test the specification of our model of individual behavior we perform four sensitivity analyses. Each of these exercises should produce a predictable response from the behavior model. Should our model replicate these expected responses, then we can be confident it is specified and implemented correctly.

First, we shutdown the disease component of the model and assess how the decision to work responds to the returns of working. Under this setup, the returns to working are captured by the relative difference in the amount an individual consumes (captured by the utility function) if they work vs not work. Since this experiment shuts down other factors that determine utility, we should see the probability an individual decides to work increases as the relative difference in the utility from working increases. This pattern is exactly what we see in Fig D panel (a), which plots the probability an individual works as a function of the difference in utility between working and not working.

Next we introduce the infectious disease dynamics into the model. For this specification the probability of getting infected is independent of the decision to work or not. Individuals that do get sick pay a utility cost while they are infected. Individuals still earn utility from consumption, which depends on their decision to work. Given this set up we should expect two patterns to emerge. First, conditional on an individual's health state (i.e., susceptible or infected) the probability of working or not should depend on the relative gap in the payoffs between both actions. This feature is driven by the independence of the infection probability and the labor supply decision. Second, being infected should act like a "fixed cost" in utility terms.

To assess whether the model captures these features in Fig D panel (b) we plot the value functions by infection state and labor supply decision as a function of the utility cost of infection. There are two takeaways. First, as the utility cost of infection increases the gap between the value functions for susceptible and infected individuals, consistent with the fixed cost of infection we expected. Second, conditional on a utility cost of infection and a health state, the gap between the value functions for working and not working is constant, which is consistent with probability of infection being the same for both individual behaviors.

The next experiment takes the prior one and allows the disease to impact the utility from working. Specifically, in addition to receiving utility from consumption, individuals that choose to work while infected pay an additional utility cost. Given this additional feature we should expect to see a response in the utility and corresponding behavior of infected individuals as the additional cost of working while infected increases. Panel (c) of Fig D reports the value functions for susceptible and infected individuals if they choose to work or not work as a function of the additional cost of working if infected. The value functions for susceptible and infected individuals that do not work are parallel, which reflects the gains from working and the penalty associated with being infected. The value function for infected individuals that choose to work declines as their penalty of working increases. As shown in Panel (d) of Fig D, these declines in the value function of working while infected translates into sharp decreases in the probability that an infected individual chooses to work. Moreover this change does not alter the probability that susceptible individuals choose to work within the model.

From here we investigate how the model reacts when allowing the probability of getting infected to depend on the decision to work or not. To isolate the effect of this feature we shutdown the additional cost of working for infected individuals that we introduced in the previous experiment. We should expect to see shifts in behaviors as the probability of infection conditional on work decision gets larger, as this will reflect individuals are rationally responding to engage in the behaviors that are less likely to get them infected and achieve greater utility. Fig E plots a heatmap of the probability a susceptible individual chooses to work as a function of the probability of getting infected if they work and not work. We note two takeaways. First, when facing the same probability of getting infected, the probability of working or not is equal. Second, as the probability of getting infected if an individual chooses to work gets larger, the probability of choosing to work falls. Related, as the probability of getting infected while not working increases, the probability an individual chooses to work rises.

Each of these exercises produced the anticipated effects. In particular, they demonstrate how individual behavior responds to its immediate payoffs as well as payoffs that materialize in later periods. These components are important for our integrated framework where individual decisions today impact aggregate disease dynamics for the next period, which in turn influence behavior.

Supplementary Tables

Table A. Infection model parameters (Ψ). Model parameters are fixed at these values unless explicitly noted in figures or tables in the paper.

Ψ	**Interpretation**	**Value**	**Unit**	**Ref**
β	The probability of transmission per infectious contact per time. This parameter is calibrated to give $R_0 = 2.6$ using the model with fixed decisions with 75% individuals working.	0.025	/day /individual	(39)
γ	Rate of recovery from infectiousness g, based on estimates of the serial interval and the duration of shedding of infectious virus centered at approximately 1 week. There is substantial variation in estimates across studies, individuals, and metrics, and infectious period is expected to depend on control measures that lead to isolation of infected individuals.	0.14	/day	(20–23)
α	Rate of waning of protective immunity after natural infection. Two systematic reviews measured the risk reduction for re-infection in individuals with documented prior infection, and suggest protection decays to 50% at 6 months, corresponding to an effective waning rate of ln(2)/180 days.	0.004	/day	(24, 25)
C_0	Baseline total number of contacts of each individual.	4		(26–28)
σ_1	Increased propensity to come into contact with individuals of the same vulnerability and socioeconomic status ("preferential mixing").	1.5		(35–38)
σ_{work}	Increased propensity to come into contact with other working individuals when deciding to work.	4		(26–28)
σ_{SES}	Increased propensity of contact between individuals of low SES. Low-SES people tend to work in industries with more in-person contacts and experience more household crowding. Value abstracted from studies to represent an intermediate degree of assortativity.	1.5		(29–34)
$\mathbb{C}$	The contact matrix C is a function of $C_0, \sigma_1, \sigma_{\text{work}}, \sigma_{\text{SES}}$.	Eq. (14)		

Table B. Decision model parameters (Θ).

Θ	Interpretation	Value	Unit	Ref
w_L	Average daily wage for low-SES individuals. When working, we assume consumption is equal to wages. The wages for low socioeconomic statuses (SES) were calculated from the 20–40% of family annual median income, which is $35,600 per year, which converts to approximately $98 per day.	98	$/day	(40)
w_h	Average daily wage for high-SES individuals. When working, we assume consumption is equal to wages. The wages for low socioeconomic statuses (SES) were calculated from the 60-80% of family annual median income, which is $95,700 per year, which converts to approximately $262 per day.	262	$/day	
$\bar{c}_L$	Baseline daily consumption (consumed when not working and not earning wages) for low SES individuals. Values are derived based on the amount of financial assets held by a family. We assume these assets are savings and define a savings rate as the fraction of these assets relative to their income. Thus we assume that low-SES individuals consume 15% of their savings when not working.	15	$/day	
$\bar{c}_H$	Baseline daily consumption (consumed when not working and not earning wages) for high SES individuals. Values are derived based on the amount of financial assets held by a family. We assume these assets are savings and define a savings rate as the fraction of these assets relative to their income. Thus we assume that high-SES individuals consume 25% of their savings when not working	66	$/day	
θ_x	Utility cost of infection. This cost is only paid when an individual's health state is infected. Given $\theta_x = -5$, the average individual in this study would be willing to pay $5,970 per day to not be in the infected state. See the Parameters section for an estimation of the daily cost of infection in dollars.	-5		(41)
θ_v	Additional utility cost of infection for vulnerable individuals. This feature is intended to capture the additional incentive vulnerable people have to avoid illness. Relative to the average non-vulnerable person in our simulated population, the average vulnerable individual would be willing to pay triple the amount of non-vulnerable to avoid infection. We intentionally set this value to be large to highlight the role of health-wealth trade-offs in our scenario.	3		
σ_h	Variance of hassle costs. We assume that hassle costs follow a log-normal distribution such that $\log h_{mt} \sim \mathcal{N}(0, \sigma_h)$. This distributional assumption has two implications. First, it imposes that hassle costs are positive. Second, there is a closed form for its expected value, which is computationally convenient.	0.25		
θ_h	Utility penalty for the hassle cost. Given the $\log h_{mt} \sim \mathcal{N}(0, \sigma_h)$ and $\theta_h = -0.5$, the main hassle cost paid by the average individual is equivalent to $116 per day, a one standard deviation increase in the hassle cost paid by the average individual in our simulated population is equivalent to $193, a one standard deviation decrease in the hassle cost paid by the average individual in our simulated population is equivalent to $73 per day. The hassle cots are calibrated to be moderate in size such that they generate variation in choices within our stylized application.	-0.5		
p_c	Additional utility cost for work if infected. Our framework assumes that the hassle costs of working are greater for infected individuals. In our model, we assume that choosing to work while infected incurs an extra 2 units of hassle cost compared to working in other infection states.	2		
κ	Discount factor. It captures the value of future flow utility payoffs today. A value of 0.96 implies individuals tend to prefer payoffs today but are still willing to wait for future period payoffs. A discount factor of 0.96 implies an annual discount rate of 4%.	0.96	/day	(51)
ν	Euler's constant	0.57721		
l	Information lag for each individual aims to capture the delay between onset and case reporting.	7	days	(52)

Table C. Estimates of value per statistical case (VSC) for COVID-19. All values are in US dollars.

**Infection severity**	**Risk of outcome**	**Value per statistical case (VSC)**	**Average VSC**	**Average daily VSC**	**Source**
Fatal	0.068%	[1] $13,100,000<br>[2] $11,000,000	$8,908<br>$7,480	$1,273<br>$1,069	Risk of outcome: (44). Value: [1] U.S. Dept of Health & Human Services (HHS) value per statistical life, 2023: (41, 42). [2] U.s. Dept of Transportation (DOT) value per statistical life, 2019 (43)
Critical	0.24%	[1] $2,113,000<br>[2] $2,021,950	$3,728<br>$3,567	$533<br>$510	Risk of outcome: Estimate from case-hospitalization-ratio (45), fraction of infections reported as cases (46), and fraction of hospitalizations involving intensive care units (ICU) ('critical'), removing fatalities (47). Value: [1] HHS VSCs, 2023: (41, 42). [2] DOT VSCs, 2019 (43)
Severe	1.0%	[1] $11,000<br>[2] $512,300	$108<br>$5,008	$15<br>$715	Risk of outcome: Same as above, but using fraction of hospitalizations not progressing to ICU. Value: as above
Mild	58.7%	[1] $5000<br>[2] $32,700	$2,935<br>$19,198	$419<br>$2,743	Risk of outcome: Infections not resulting in death or hospitalization (above), and not being asymptomatic (below). Value: as above
Asymptomatic	40%	[1] $0<br>[2] $10,900	$0<br>$4,360	$0<br>$623	Risk of outcome: (48). Value: [1] assumed zero, [2] as above
Overall		[1]<br>[2]	$15,679<br>$39,613	$2,240<br>$5,679	

Table D. A summary of policy scenarios and resulting cost breakdown by income loss and subsidy payment. Values in parentheses indicate the proportion of each cost component to the total cost. Total daily cost per capita includes both lost wages due to the disease (compared to a disease-free scenario with 75% labor supply) and the cost of any subsidy payments provided.

**Policy**	**Policy Scenario**	**Daily wage loss per capita**	**Daily subsidy payment per capita**	**Total daily cost per capita**
No intervention	No policy applied	\$13.0 (100%)	0 (0%)	\$13.0
Labor restriction	30% Labor restriction	\$36.3 (100%)	0 (0%)	\$36.3
	40% Labor restriction	\$44.4 (100%)	0 (0%)	\$44.4
	50% Labor restriction	\$53.2 (100%)	0 (0%)	\$53.2
	60% Labor restriction	\$62.1 (100%)	0 (0%)	\$62.1
	70% Labor restriction	\$72.0 (100%)	0 (0%)	\$72.0
Unconditional cash transfer	Payment = 10% average wage	\$19.9 (53%)	\$18.0 (47%)	\$37.9
	Payment = 20% average wage	\$24.5 (40%)	\$36.0 (60%)	\$60.5
	Payment = 30% average wage	\$27.2 (34%)	\$54.0 (66%)	\$81.2
	Payment = 40% average wage	\$29.8 (29%)	\$72.0 (71%)	\$101.8
	Payment = 50% average wage	\$31.3 (26%)	\$90.0 (74%)	\$121.3
Conditional cash transfer	Payment = 10% average wage	\$22.0 (73%)	\$8.0 (27%)	\$30.0
	Payment = 20% average wage	\$28.3 (61%)	\$18.0 (39%)	\$46.3
	Payment = 30% average wage	\$33.7 (53%)	\$29.4 (47%)	\$63.1
	Payment = 40% average wage	\$38.6 (48%)	\$42.0 (52%)	\$80.6
	Payment = 50% average wage	\$41.9 (43%)	\$54.7 (57%)	\$96.6
Paid sick leave	Payment = 10% average wage	\$11.4 (96%)	\$0.8 (4%)	\$12.2
	Payment = 20% average wage	\$10.2 (86%)	\$1.6 (14%)	\$11.8
	Payment = 30% average wage	\$9.3 (81%)	\$2.2 (19%)	\$11.5
	Payment = 40% average wage	\$8.5 (75%)	\$2.9 (25%)	\$11.4
	Payment = 50% average wage	\$7.9 (70%)	\$3.4 (30%)	\$11.3

Table E. Comparison of FIEM (shotdown decision model) and Theoretical Compartmental Model under different parameter settings. FIEM is initialized with a single risk group to align with the assumptions of the theoretical compartmental model for direct comparison under varying parameter settings.

Model	Transmission rate	Rate of recovery	Rate of waning	R_0	Peak size	Peak time
FIEM	0.30	0.14	0.0043	2.11	18.24%	45
Compartmental Model				2.14	18.18%	44
FIEM	0.30	0.14	0	2.12	17.88%	45
Compartmental Model				2.14	17.81%	43
FIEM	0.275	0.14	0	1.99	14.88%	50
Compartmental Model				1.96	14.76%	50
FIEM	0.25	0.14	0	1.99	11.15%	62
Compartmental Model				1.79	11.59%	58
FIEM	0.225	0.14	0	1.69	8.26%	68
Compartmental Model				1.61	8.32%	70
FIEM	0.2	0.14	0	1.46	4.90%	93
Compartmental Model				1.43	5.10%	90
FIEM	0.175	0.14	0	1.26	2.28%	129
Compartmental Model				1.25	2.23%	128
FIEM	0.15	0.14	0	1.08	0.38%	193
Compartmental Model				1.07	0.32%	202
FIEM	0.125	0.14	0	<1	–	–
Compartmental Model						

Supplemental Figures

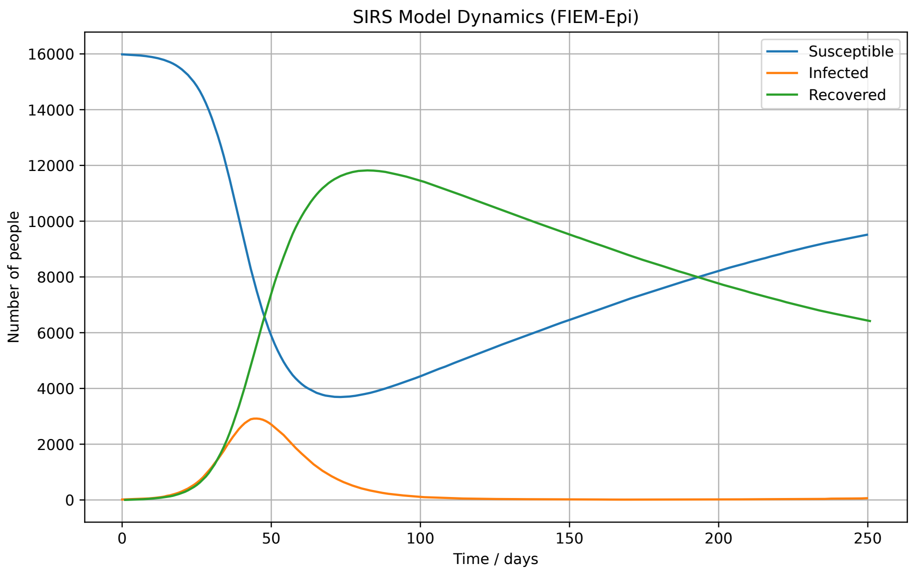

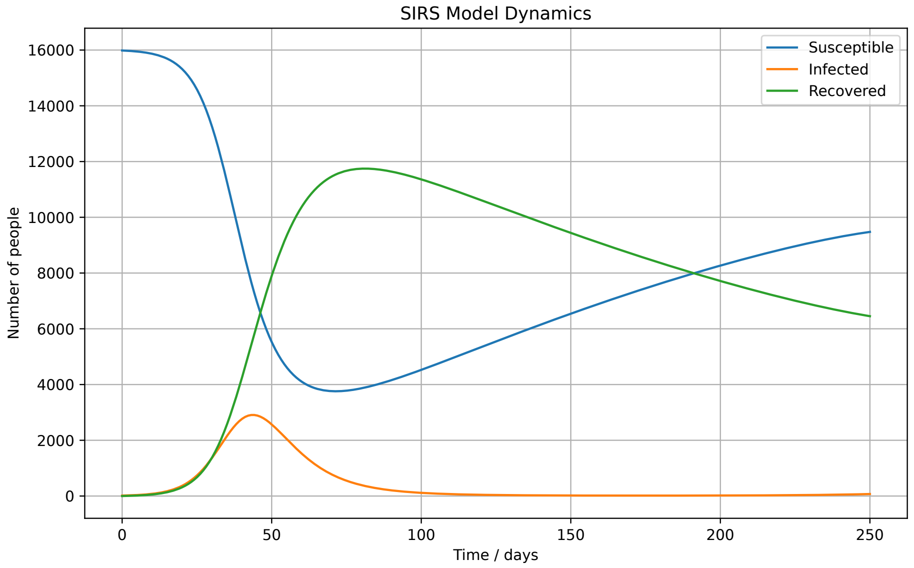

Fig A. Comparison of FIEM-Epi (shotdown decision model) and Theoretical Compartmental Model. Here, the transmission rate = 0.3, the rate of recovery = 0.14, and the rate of waning = 0.0043. FIEM is initialized with a single risk group to align with the assumptions of the theoretical compartmental model for direct comparison.

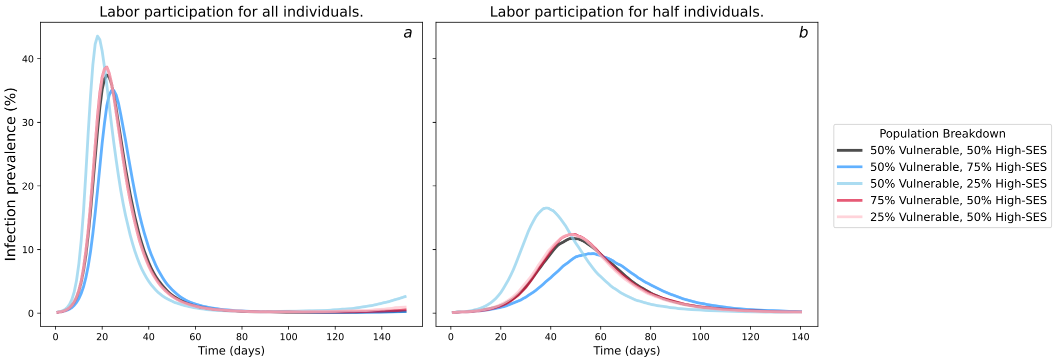

Fig B. Model testing with fixed work decisions. This figure shows the infection prevalence (%) under two fixed labor supply scenarios: (a) infection prevalence when all individuals are participating in the workforce, and (b) infection prevalence when only half of the individuals are participating. The color scale represents different initial population distributions with varying heterogeneous group sizes.

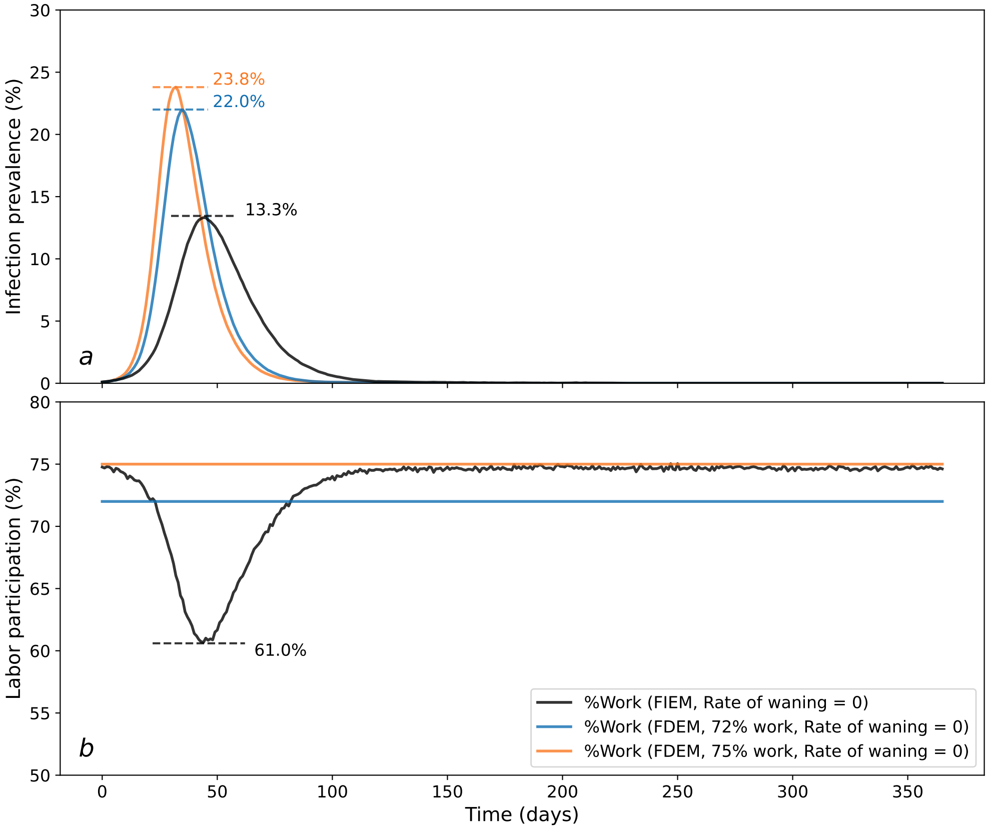

Fig C. Effect of endogenous behavior change on disease dynamics with waning rate (α) = 0. The time-course of infection and employment levels for the feedback-informed epidemiological model. (a) The share of the population that is infected under different models. (b) The share of the population that choose to work each period under different models.

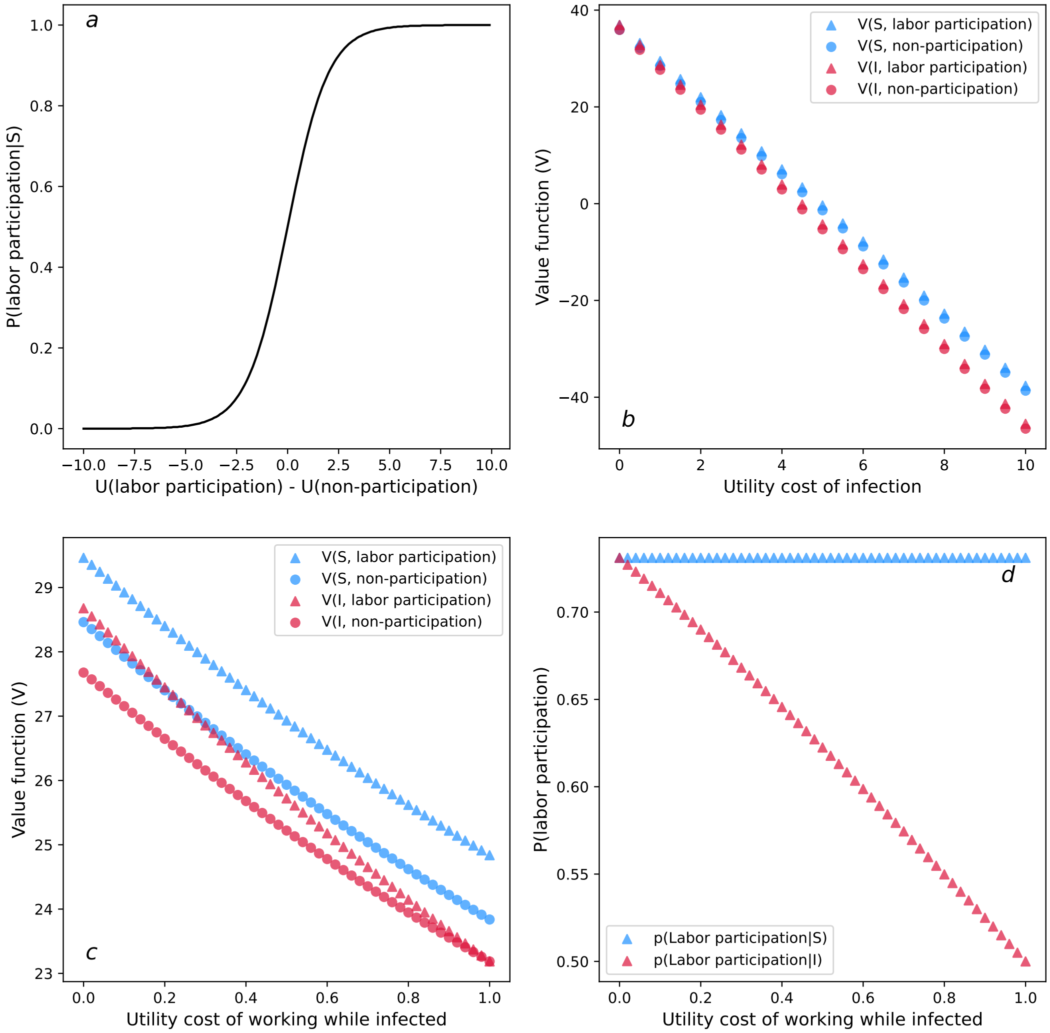

Fig D. Model validation for individual work behaviors. This figure analyzes labor supply under four distinct scenarios: (a) Baseline scenario without disease dynamics: Assess how work decisions respond to the utility difference between working and not working without considering the impact of disease. (b) Disease with fixed infection probability: Assume a constant probability of infection, independent of individual behavior. The plot illustrates the value functions of infection state and labor supply decision as a function of the utility cost of infection. (c) Infection impacts utility from working: Individuals working while infected face an additional utility cost beyond the utility from consumption. The plot shows value functions by infection state and work decision as a function of the utility cost of infection. (d) Labor supply vs. utility cost of working while infected: Under the same conditions as in (c), this plot depicts labor supply rates as a function of the utility cost of working while infected.

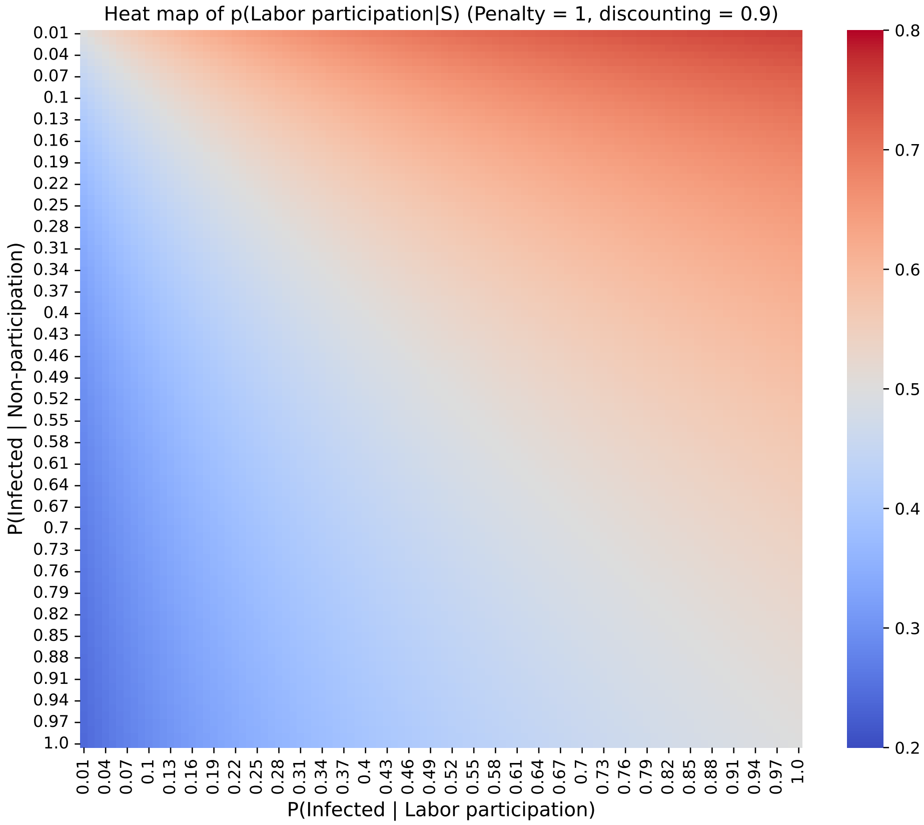

Fig E. Heatmap of labor supply decisions based on infection probability. The x-axis represents the probability of getting infected while participating in the workforce, and the y-axis represents the probability of getting infected while not participating. The color scale indicates the probability of choosing to work, with redder colors denoting a higher likelihood of working.

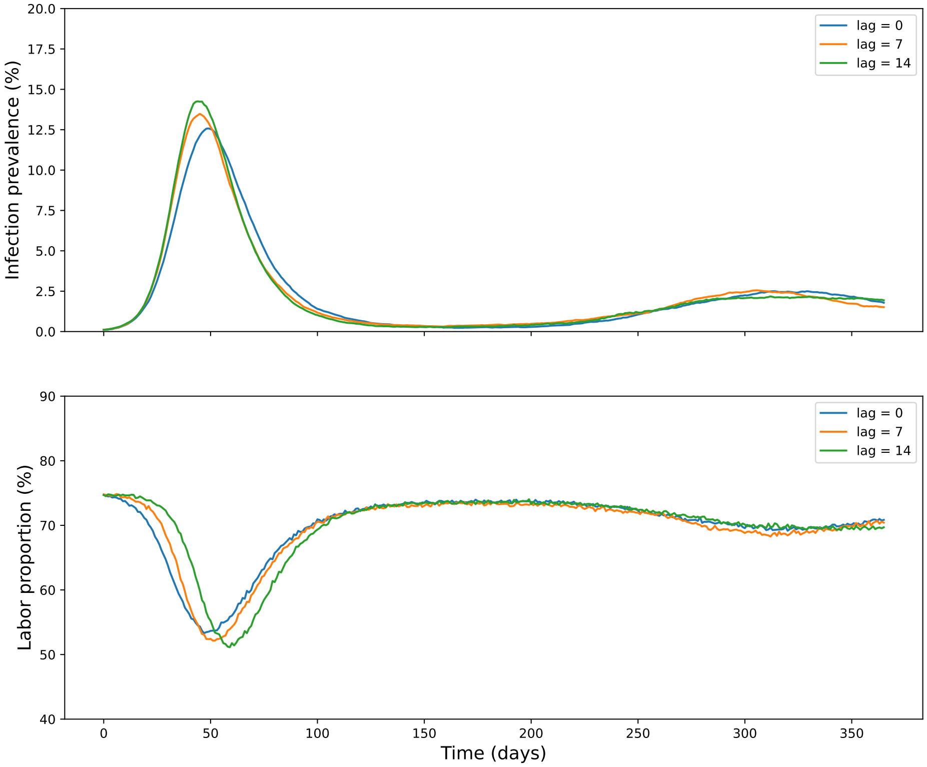

Fig F. Sensitivity analysis to the amount of information individuals have about the aggregate infection rates when making decisions. A lag of 0 means individuals know the true aggregate infection rate in real time, a lag of 7 means individuals know the aggregate infection rate from 7 periods prior to the current one, and a lag of 14 means individuals know the aggregate infection rate 14 periods prior to the current one. The top panel reports the share of the population that is infected and the bottom panel reports the share of the population that works under these three information structures.

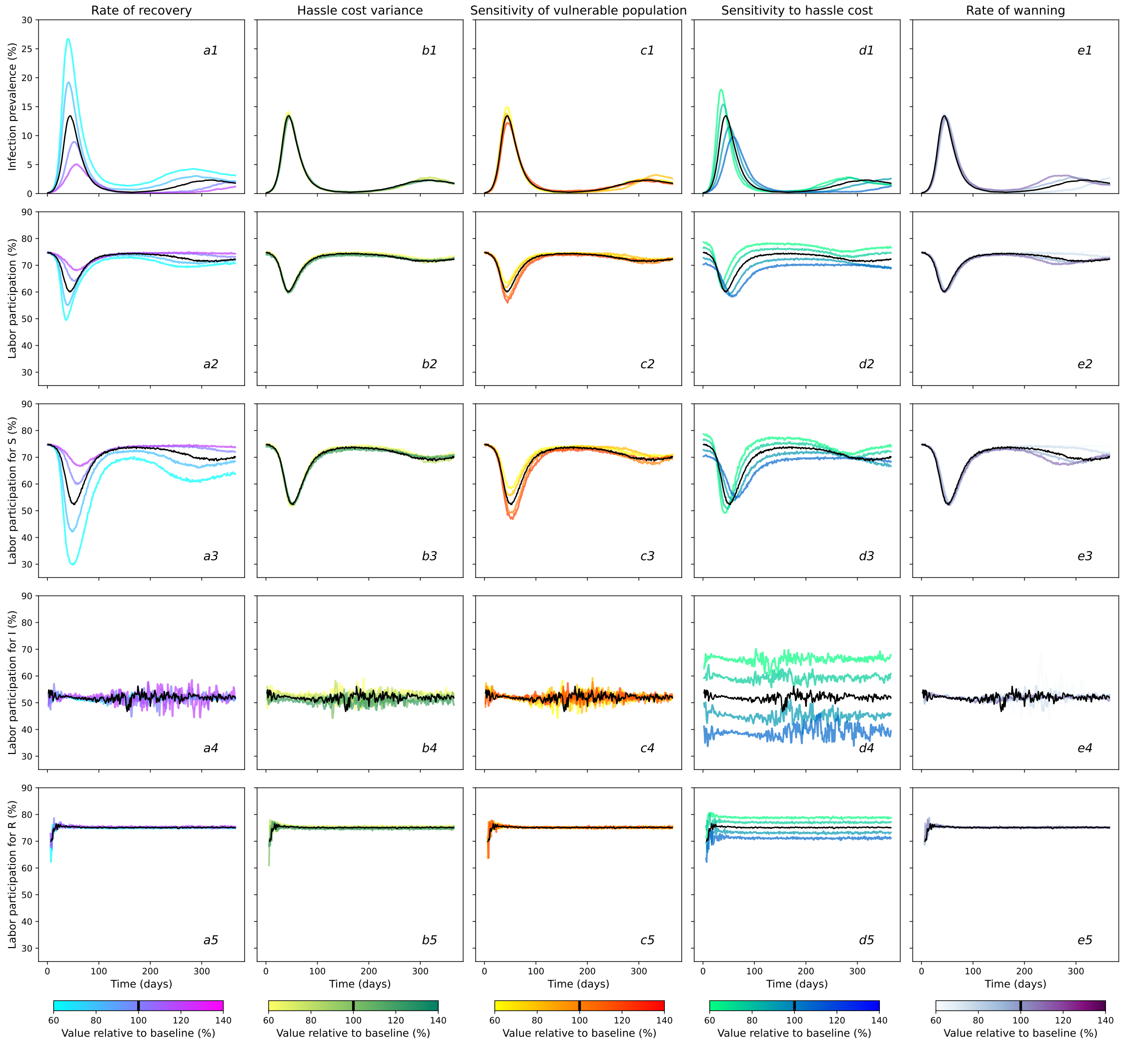

Fig G. Sensitivity analysis of selected model parameters. The black curves in each panel show our baseline parameter values as given in Tables B and A. Row (1) reports the population infection rate, Row (2) reports the share of the population that choose to work, and rows (3–5) report the work decisions by susceptible, infected and recovered groups. Column (a) varies the recovery rate, (b) alters the variance of hassle cost, (c) varies the sensitivity of vulnerable populations to the infection risk, (d) alters the sensitivity of hassle cost, and (e) varies the immunity winning rate.

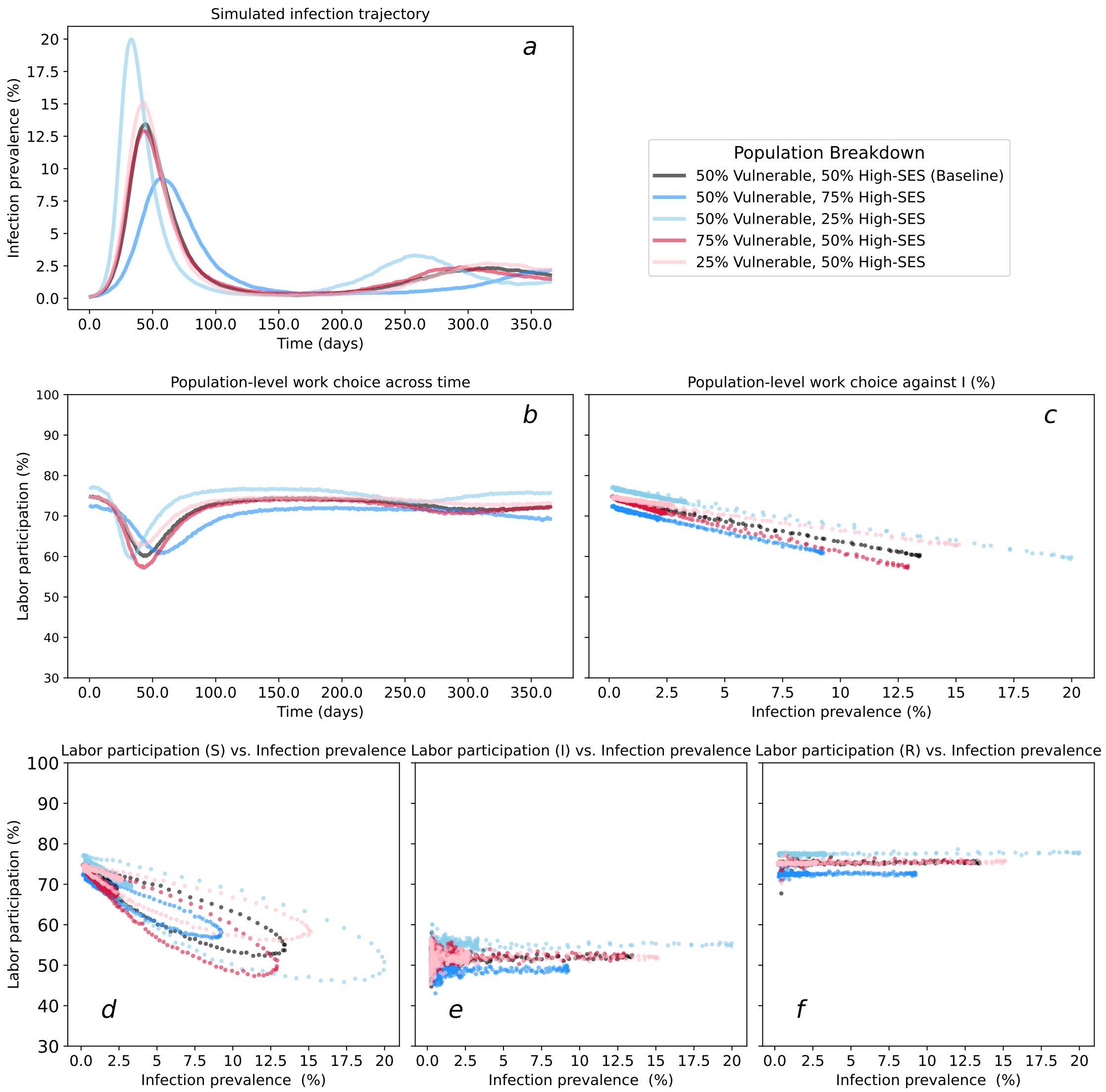

Fig H. Illustration of decision dynamics with heterogeneous populations. Panel (a) captures the share of the population that is infected; (b) includes the share of the population that choose to work; (c) shows the population infection rate against the share of the population that choose to work; (d–f) show the work choice probabilities for susceptible, infected, and recovered health state groups respectively. Five populations are simulated and differ based on the distribution of non-infection characteristics (i.e., vulnerability to the disease and SES). The black curve represents the baseline parameter values.

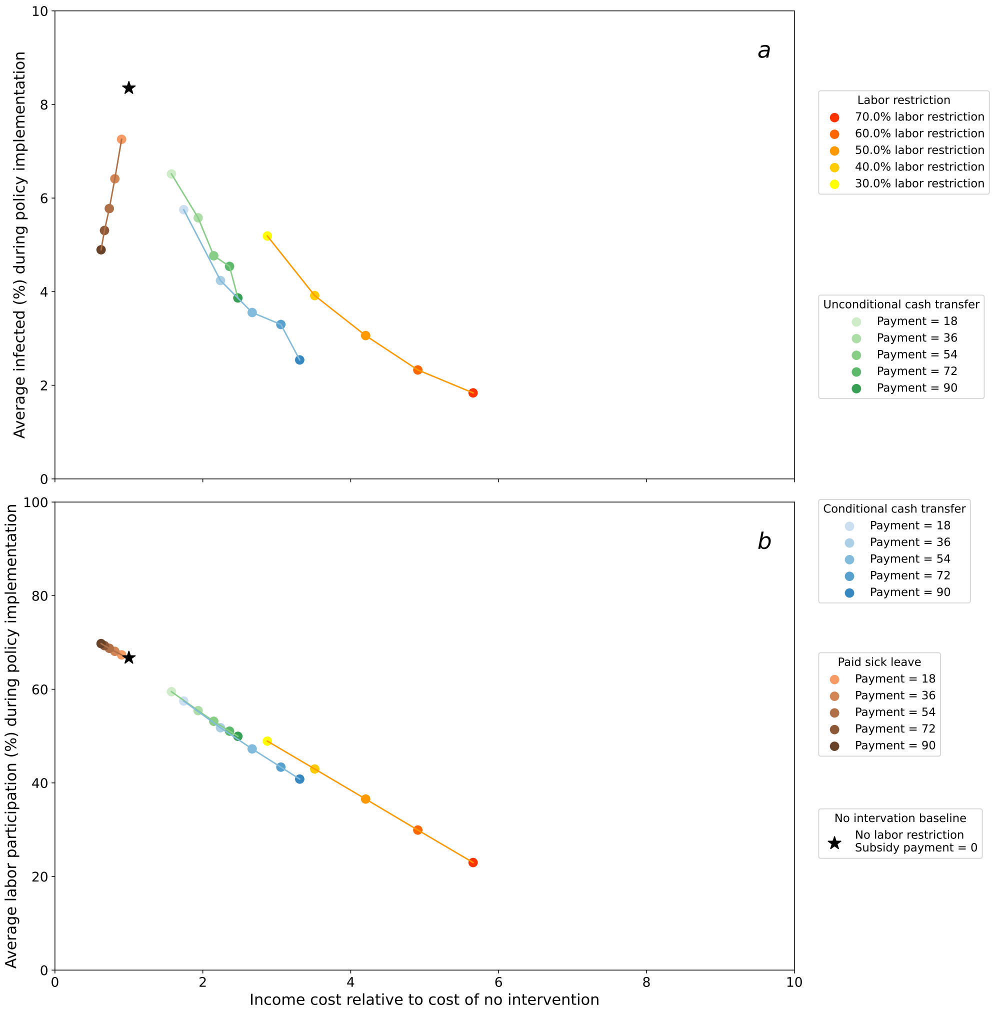

Fig I. Average infection and labor supply by different policy interventions. This figure plots the average share of the population that is infected and working under different policy interventions as a function of their income loss. Panel a plots the average infection rates over the time period when the policies are in effect, while panel b plots the average share of the population that chose to work during this time period. The cost captures lost wages an individual experiences relative to their baseline wage earnings predicted by the model when there is no intervention. The simulated policies include: a labor restriction policy that limits how much of the population is able to choose to work; an unconditional cash transfer that is delivered to all individuals each period; a conditional cash transfer that is delivered to individuals that choose to not work each period; and a paid sick leave policy, which allows infected individuals to earn their full wage if they choose to not work while infected.

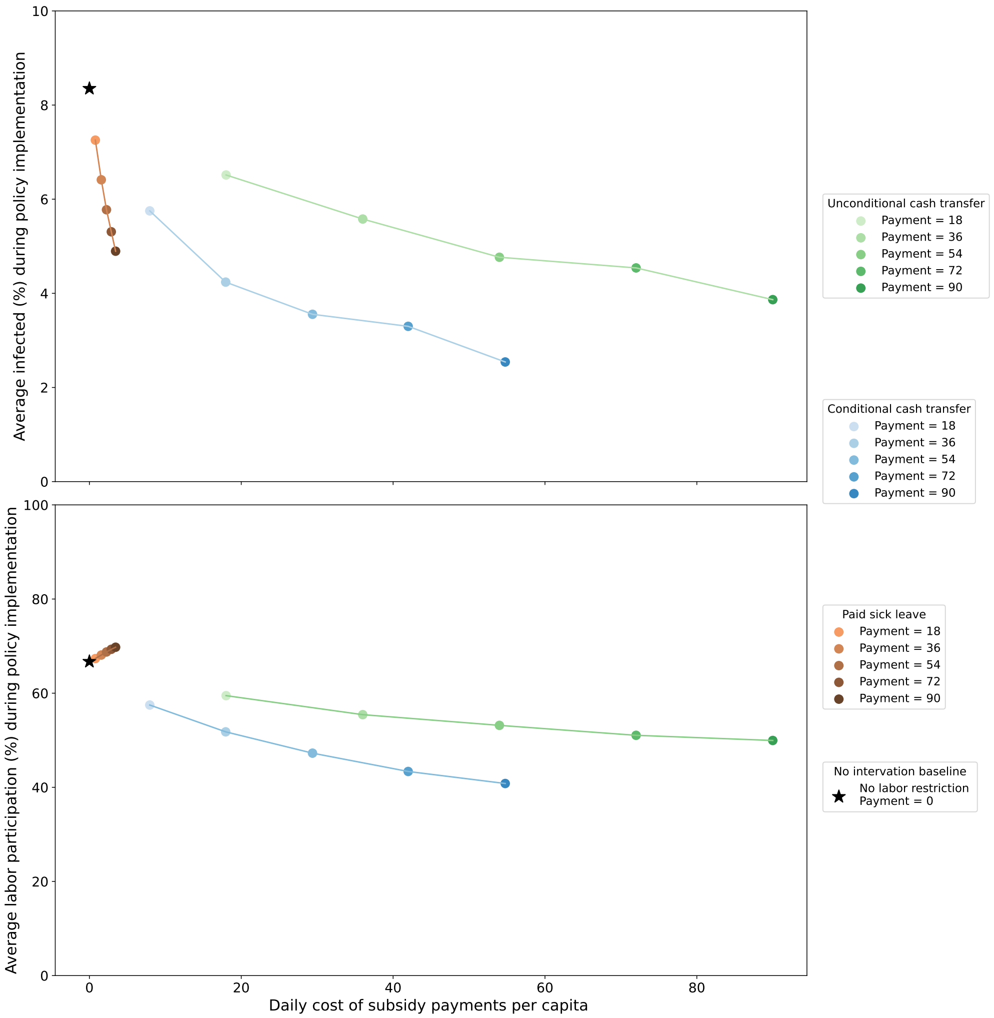

Fig J. Average infection and labor supply by different policy interventions. This figure plots the average share of the population that is infected and working under different policy interventions as a function of their income loss. Panel a plots the average infection rates over the time period when the policies are in effect, while panel b plots the average share of the population that chose to work during this time period. The cost captures government spending to fund these cash transfers. The simulated policies include: a labor restriction policy that limits how much of the population is able to choose to work; an unconditional cash transfer that is delivered to all individuals each period; a conditional cash transfer that is delivered to individuals that choose to not work each period; and a paid sick leave policy, which allows infected individuals to earn their full wage if they choose to not work while infected.

References

[1] Roy M. Anderson and Robert M. May. *Infectious Diseases of Humans: Dynamics and Control.* Oxford University Press, Oxford, 1991.

[2] Matt J. Keeling and Pejman Rohani. *Modeling Infectious Diseases in Humans and Animals.* Princeton University Press, Princeton, NJ, 2008.

[3] Daniel McFadden. Conditional logit analysis of qualitative choice behavior. In Paul Zarembka, editor, *Frontiers in Econometrics*, pages 105–142. Academic Press: New York, 1974.

[4] John Rust. Optimal replacement of gmc bus engines: An empirical model of harold zurcher. *Econometrica*, 55(5):999–1033, 1987.

[5] Kenneth E. Train. *Discrete Choice Methods with Simulation.* Cambridge university press, 2009.

[6] Richard Bellman. *Dynamic Programming.* Princeton University Press, Princeton, N.J., 1957.

[7] Avinash K Dixit. *Optimization in Economic Theory.* Oxford Univeristy Press, 1990.

[8] V. Joseph Hotz and Robert A. Miller. Conditional choice probabilities and the estimation of dynamic models. *The Review of Economic Studies*, 60(3):497–529, 1993.

[9] Thierry Magnac and David Thesmar. Identifying dynamic discrete decision processes. *Econometrica*, 70(2):801–816, 2002.

[10] Board of Governors of the Federal Reserve Board. 2022 survey of consumer finances, 2023. Available at https://www.federalreserve.gov/econres/scfindex.htm.

[11] Elena Druică, Fabio Musso, and Rodica Ianole-Călin. Optimism bias during the covid-19 pandemic: Empirical evidence from romania and italy. *Games*, 11(3), 2020. ISSN 2073-4336. doi: 10.3390/g11030039.

[12] Iro Fragkaki, Dominique F. Maciejewski, Esther L. Weijman, Jonas Feltes, and Maaike Cima. Human responses to covid-19: The role of optimism bias, perceived severity, and anxiety. *Personality and Individual Differences*, 176:110781, 2021. ISSN 0191-8869. doi: https://doi.org/10.1016/j.paid.2021.110781. URL https://www.sciencedirect.com/science/article/pii/S0191886921001562.

[13] Kathleen McColl, Marion Debin, Cecile Souty, Caroline Guerrisi, Clement Turbelin, Alessandra Falchi, Isabelle Bonmarin, Daniela Paolotti, Chinelo Obi, Jim Duggan, Yamir Moreno, Ania Wisniak, Antoine Flahault, Thierry Blanchon, Vittoria Colizza, and Jocelyn Raude. Are people optimistically biased about the risk of COVID-19 infection? lessons from the first wave of the pandemic in europe. *Int J Environ Res Public Health*, 19(1), December 2021.

[14] Kathy Leung, Joseph T Wu, and Gabriel M Leung. Real-time tracking and prediction of covid-19 infection using digital proxies of population mobility and mixing. *Nature communications*, 12(1):1501, 2021.

[15] Louise Dyson, Edward M Hill, Sam Moore, Jacob Curran-Sebastian, Michael J Tildesley, Katrina A Lythgoe, Thomas House, Lorenzo Pellis, and Matt J Keeling. Possible future waves of sars-cov-2 infection generated by variants of concern with a range of characteristics. *Nature Communications*, 12(1):5730, 2021.

[16] Tijs W. Alleman, Michiel Rollier, Jenna Vergeynst, and Jan M. Baetens. A stochastic mobility-driven spatially explicit SEIQRD COVID-19 model with VOCs, seasonality, and vaccines. *Applied Mathematical Modelling*, 123:507–525, 2023. ISSN 0307-904X. doi: https://doi.org/10.1016/j.apm.2023.06.027.

[17] John Rust. Structural estimation of markov decision processes. In *Handbook of Econometrics*, volume 4, chapter 51, pages 3081–3143. Elsevier, Amsterdam, 1994.

[18] Peter Arcidiacono and Paul B Ellickson. Practical methods for estimation of dynamic discrete choice models. *Annual Review Economics*, 3(1):363–394, 2011.

[19] M. Begon, M. Bennett, R. G. Bowers, N. P. French, S. M. Hazel, and J. Turner. A clarification of transmission terms in host-microparasite models: Numbers, densities and areas. *Epidemiology and Infection*, 129(1):147–153, 2002.

[20] Andrew William Byrne, David McEvoy, Aine B Collins, Kevin Hunt, Miriam Casey, Ann Barber, Francis Butler, John Griffin, Elizabeth A Lane, Conor McAloon, et al. Inferred duration of infectious period of sars-cov-2: rapid scoping review and analysis of available evidence for asymptomatic and symptomatic covid-19 cases. *BMJ open*, 10(8):e039856, 2020.

[21] Sheikh Taslim Ali, Lin Wang, Eric H. Y. Lau, Xiao-Ke Xu, Zhanwei Du, Ye Wu, Gabriel M. Leung, and Benjamin J. Cowling. Serial interval of SARS-CoV-2 was shortened over time by nonpharmaceutical interventions. *Science*, July 2020. ISSN 0036-8075, 1095-9203. doi: 10.1126/science.abc9004. URL https://science.sciencemag.org/content/early/2020/07/20/science.abc9004. Publisher: American Association for the Advancement of Science Section: Report.

[22] Seran Hakki, Jie Zhou, Jakob Jonnerby, Anika Singanayagam, Jack L. Barnett, Kieran J. Madon, Aleksandra Koycheva, Christine Kelly, Hamish Houston, Sean Nevin, Joe Fenn, Rhia Kundu, Michael A. Crone, Timesh D. Pillay, Shazaad Ahmad, Nieves Derqui-Fernandez, Emily Conibear, Paul S. Freemont, Graham P. Taylor, Neil Ferguson, Maria Zambon, Wendy S. Barclay, Jake Dunning, Ajit Lalvani, Anjna Badhan, Robert Varro, Constanta Luca, Valerie Quinn, Jessica Cutajar, Niamh Nichols, Jessica Russell, Holly Grey, Anjeli Ketkar, Giulia Miserocchi, Chitra Tejpal, Harriet Catchpole, Koji Nixon, Berenice Di Biase, Tamara Hopewell, Janakan Sam Narean, Jada Samuel, Kristel Timcang, Eimear McDermott, Samuel Bremang, Sarah Hammett, Samuel Evetts, and Alexandra Kondratiuk. Onset and window of SARS-CoV-2 infectiousness and temporal correlation with symptom onset: a prospective, longitudinal, community cohort study. *The Lancet Respiratory Medicine*, 10(11):1061–1073, November 2022. ISSN 2213-2600, 2213-2619. doi: 10.1016/S2213-2600(22)00226-0. URL https://www.thelancet.com/journals/lanres/article/PIIS2213-2600(22)00226-0/fulltext. Publisher: Elsevier.

[23] Enrico Lavezzo, Elisa Franchin, Constanze Ciavarella, Gina Cuomo-Dannenburg, Luisa Barzon, Claudia Del Vecchio, Lucia Rossi, Riccardo Manganelli, Arianna Loregian, Nicolò Navarin, Davide Abate, Manuela Sciro, Stefano Merigliano, Ettore De Canale, Maria Cristina Vanuzzo, Valeria Besutti, Francesca Saluzzo, Francesco Onelia, Monia Pacenti, Saverio Parisi, Giovanni Carretta, Daniele Donato, Luciano Flor, Silvia Cocchio, Giulia Masi, Alessandro Sperduti, Lorenzo Cattarino, Renato Salvador, Michele Nicoletti, Federico Caldart, Gioele Castelli, Eleonora Nieddu, Beatrice Labella, Ludovico Fava, Matteo Drigo, Katy A. M. Gaythorpe, Imperial College COVID-19 Response Team, Alessandra R. Brazzale, Stefano Toppo, Marta Trevisan, Vincenzo Baldo, Christl A. Donnelly, Neil M. Ferguson, Ilaria Dorigatti, and Andrea Crisanti. Suppression of a SARS-CoV-2 outbreak in the Italian municipality of Vo'. *Nature*, June 2020. ISSN 1476-4687. doi: 10.1038/s41586-020-2488-1.

[24] Caroline Stein, Hasan Nassereldine, Reed J. D. Sorensen, Joanne O. Amlag, Catherine Bisignano, Sam Byrne, Emma Castro, Kaleb Coberly, James K. Collins, Jeremy Dalos, Farah Daoud, Amanda Deen, Emmanuela Gakidou, John R. Giles, Erin N. Hulland, Bethany M. Huntley, Kasey E. Kinzel, Rafael Lozano, Ali H. Mokdad, Tom Pham, David M. Pigott, Robert C. Reiner Jr, Theo Vos, Simon I. Hay, Christopher J. L. Murray, and Stephen S. Lim. Past SARS-CoV-2 infection protection against re-infection: a systematic review and meta-analysis. *The Lancet*, 401(10379):833–842, March 2023. ISSN 0140-6736, 1474-547X. doi: 10.1016/S0140-6736(22)02465-5. URL https://www.thelancet.com/journals/lancet/article/PIIS0140-6736(22)02465-5/fulltext. Publisher: Elsevier.

[25] Niklas Bobrovitz, Harriet Ware, Xiaomeng Ma, Zihan Li, Reza Hosseini, Christian Cao, Anabel Selemon, Mairead Whelan, Zahra Premji, Hanane Issa, Brianna Cheng, Laith J. Abu Raddad, David L. Buckeridge, Maria D. Van Kerkhove, Vanessa Piechotta, Melissa M. Higdon, Annelies Wilder-Smith, Isabel Bergeri, Daniel R. Feikin, Rahul K. Arora, Minal K. Patel, and Lorenzo Subissi. Protective effectiveness of previous SARS-CoV-2 infection and hybrid immunity against the omicron variant and severe disease: a systematic review and meta-regression. *The Lancet Infectious Diseases*, 0(0), January 2023. ISSN 1473-3099, 1474-4457. doi: 10.1016/S1473-3099(22)00801-5. URL https://www.thelancet.com/journals/laninf/article/PIIS1473-3099(22)00801-5/fulltext. Publisher: Elsevier.

[26] Dina Mistry, Maria Litvinova, Ana Pastore y Piontti, Matteo Chinazzi, Laura Fumanelli, Marcelo FC Gomes, Syed A Haque, Quan-Hui Liu, Kunpeng Mu, Xinyue Xiong, M. Elizabeth Halloran, Ira M. Longini Jr., Stefano Merler, Marco Ajelli, and Alessandro Vespignani. Inferring high-resolution human mixing patterns for disease modeling. *Nature Communications*, 12(1):323, 2021.

[27] Joël Mossong, Niel Hens, Mark Jit, Philippe Beutels, Kari Auranen, Rafael Mikolajczyk, Marco Massari, Stefania Salmaso, Gianpaolo Scalia Tomba, Jacco Wallinga, Janneke Heijne, Malgorzata Sadkowska-Todys, Magdalena Rosinska, and W. John Edmunds. Social Contacts and Mixing Patterns Relevant to the Spread of Infectious Diseases. *PLOS Medicine*, 5(3):e74, March 2008. ISSN 1549-1676. doi: 10.1371/journal.pmed.0050074. URL https://journals.plos.org/plosmedicine/article?id=10.1371/journal.pmed.0050074.

[28] Kiesha Prem, Alex R Cook, and Mark Jit. Projecting social contact matrices in 152 countries using contact surveys and demographic data. *PLoS computational biology*, 13(9):e1005697, 2017.

[29] Nicholas W. Papageorge, Matthew V. Zahn, Michèle Belot, Eline van den Broek-Altenburg, Syngjoo Choi, Julian C. Jamison, and Egon Tripodi. Socio-demographic factors associated with self-protecting behavior during the covid-19 pandemic. *Journal of Population Economics*, Forthcoming, 2021.

[30] Jonathan I. Dingel and Brent Neiman. How many jobs can be done at home? *Journal of Public Economics*, 189:104235, 2020. ISSN 0047-2727. doi: https://doi.org/10.1016/j.jpubeco.2020.104235.

[31] Anjalika Nande, Justin Sheen, Emma L. Walters, Brennan Klein, Matteo Chinazzi, Andrei H. Gheorghe, Ben Adlam, Julianna Shinnick, Maria Florencia Tejeda, Samuel V. Scarpino, Alessandro Vespignani, Andrew J. Greenlee, Daniel Schneider, Michael Z. Levy, and Alison L. Hill. The effect of eviction moratoria on the transmission of SARS-CoV-2. *Nature Communications*, 12(1):2274, April 2021. ISSN 2041-1723. doi: 10.1038/s41467-021-22521-5. URL https://www.nature.com/articles/s41467-021-22521-5. Number: 1 Publisher: Nature Publishing Group.

[32] Jonathan Jay, Jacob Bor, Elaine O. Nsoesie, Sarah K. Lipson, David K. Jones, Sandro Galea, and Julia Raifman. Neighbourhood income and physical distancing during the COVID-19 pandemic in the United States. *Nature Human Behaviour*, pages 1–9, November 2020. ISSN 2397-3374. doi: 10.1038/s41562-020-00998-2. URL https://www.nature.com/articles/s41562-020-00998-2. Publisher: Nature Publishing Group.

[33] Christina Kamis, Allison Stolte, Jessica S. West, Samuel H. Fishman, Taylor Brown, Tyson Brown, and Heather R. Farmer. Overcrowding and COVID-19 mortality across U.S. counties: Are disparities growing over time? *SSM - Population Health*, 15:100845, September 2021. ISSN 2352-8273. doi: 10.1016/j.ssmph.2021.100845. URL https://www.sciencedirect.com/science/article/pii/S2352827321001208.

[34] Ukachi N. Emeruwa, Samsiya Ona, Jeffrey L. Shaman, Amy Turitz, Jason D. Wright, Cynthia Gyamfi-Bannerman, and Alexander Melamed. Associations Between Built Environment, Neighborhood Socioeconomic Status, and SARS-CoV-2 Infection Among Pregnant Women in New York City. *JAMA*, 324(4):390–392, July 2020. ISSN 0098-7484. doi: 10.1001/jama.2020.11370. URL https://doi.org/10.1001/jama.2020.11370.

[35] Miller McPherson, Lynn Smith-Lovin, and James M. Cook. Birds of a Feather: Homophily in Social Networks. *Annual Review of Sociology*, 27(Volume 27, 2001):415–444, August 2001. ISSN 0360-0572, 1545-2115. doi: 10.1146/annurev.soc.27.1.415. URL https://www.annualreviews.org/content/journals/10.1146/annurev.soc.27.1.415. Publisher: Annual Reviews.

[36] M. E. J. Newman. Assortative Mixing in Networks. *Physical Review Letters*, 89(20):208701, October 2002. doi: 10.1103/PhysRevLett.89.208701. URL https://link.aps.org/doi/10.1103/PhysRevLett.89.208701. Publisher: American Physical Society.

[37] Jeffrey A. Smith, Miller McPherson, and Lynn Smith-Lovin. Social Distance in the United States: Sex, Race, Religion, Age, and Education Homophily among Confidants, 1985 to 2004. *American Sociological Review*, 79(3):432–456, June 2014. ISSN 0003-1224. doi: 10.1177/0003122414531776. URL https://doi.org/10.1177/0003122414531776. Publisher: SAGE Publications Inc.

[38] Rafiazka Millanida Hilman, Gerardo Iñiguez, and Márton Karsai. Socioeconomic biases in urban mixing patterns of US metropolitan areas. *EPJ Data Science*, 11(1):1–18, December 2022. ISSN 2193-1127. doi: 10.1140/epjds/s13688-022-00341-x. URL https://epjdatascience.springeropen.com/articles/10.1140/epjds/s13688-022-00341-x. Number: 1 Publisher: SpringerOpen.

[39] Bibha Dhungel, Md Shafiur Rahman, Md Mahfuzur Rahman, Aliza KC Bhandari, Phuong Mai Le, Nushrat Alam Biva, and Stuart Gilmour. Reliability of early estimates of the basic reproduction number of covid-19: a systematic review and meta-analysis. *International journal of environmental research and public health*, 19(18):11613, 2022.

[40] Board of Governors of the Federal Reserve System. Survey of consumer finances (scf). https://www.federalreserve.gov/econres/scfindex.htm, 2024. Accessed: 2024-07-10.

[41] Kearsley A. Hhs standard values for regulatory analysis | aspe. Available from: https://aspe.hhs.gov/reports/standard-ria-values, 2024.

[42] Lisa A. Robinson, Michael R. Eber, and James K. Hammitt. Valuing COVID-19 Morbidity Risk Reductions. *Journal of Benefit-Cost Analysis*, 13(2):247–268, July 2022. ISSN 2194-5888, 2152-2812. doi: 10.1017/bca.2022.11. URL https://www.cambridge.org/core/journals/journal-of-benefit-cost-analysis/article/valuing-covid19-morbidity-risk-reductions/DA88757B384F9C9C70393974D8D8EC9F.

[43] Thomas J. Kniesner and Ryan Sullivan. The forgotten numbers: A closer look at COVID-19 non-fatal valuations. *Journal of Risk and Uncertainty*, 61(2):155–176, 2020. ISSN 0895-5646. URL https://www.jstor.org/stable/45379043. Publisher: Springer.

[44] Andrew T Levin, William P Hanage, Nana Owusu-Boaitey, Kensington B Cochran, Seamus P Walsh, and Gideon Meyerowitz-Katz. Assessing the age specificity of infection fatality rates for covid-19: systematic review, meta-analysis, and public policy implications. *European journal of epidemiology*, 35 (12):1123–1138, 2020.

[45] Isabel Griffin, Jessica King, B Casey Lyons, Alyson L Singleton, Xidong Deng, Beau B Bruce, and Patricia M Griffin. Estimates of sars-cov-2 hospitalization and fatality rates in the prevaccination period, united states. *Emerging Infectious Diseases*, 30(6):1144, 2024.

[46] CDC. Commercial Laboratory Seroprevalence Survey Data: National Commercial Lab Survey, March 2020. URL https://covid.cdc.gov/covid-data-tracker/#national-lab.

[47] CDC. COVID-NET, June 2025. URL https://www.cdc.gov/covid/php/covid-net/index.html.

[48] Qiuyue Ma, Jue Liu, Qiao Liu, Liangyu Kang, Runqing Liu, Wenzhan Jing, Yu Wu, and Min Liu. Global Percentage of Asymptomatic SARS-CoV-2 Infections Among the Tested Population and Individuals With Confirmed COVID-19 Diagnosis: A Systematic Review and Meta-analysis. *JAMA Network Open*, 4(12):e2137257, December 2021. ISSN 2574-3805. doi: 10.1001/jamanetworkopen.2021.37257. URL https://doi.org/10.1001/jamanetworkopen.2021.37257.

[49] Jin-Tan Liu, James K. Hammitt, Jung-Der Wang, and Meng-Wen Tsou. Valuation of the risk of SARS in Taiwan. *Health Economics*, 14(1):83–91, 2005. ISSN 1099-1050. doi: 10.1002/hec.911. URL https://onlinelibrary.wiley.com/doi/abs/10.1002/hec.911. _eprint: https://onlinelibrary.wiley.com/doi/pdf/10.1002/hec.911.

[50] Dorte Gyrd-Hansen, Peder Andreas Halvorsen, and Ivar Sønbø Kristiansen. Willingness-to-pay for a statistical life in the times of a pandemic. *Health Economics*, 17(1):55–66, 2008. ISSN 1099-1050. doi: 10.1002/hec.1236. URL https://onlinelibrary.wiley.com/doi/abs/10.1002/hec.1236. _eprint: https://onlinelibrary.wiley.com/doi/pdf/10.1002/hec.1236.

[51] Shane Frederick, George Loewenstein, and Ted O'donoghue. Time discounting and time preference: A critical review. *Journal of economic literature*, 40(2):351–401, 2002.

[52] Sam Abbott, Joel Hellewell, Robin N Thompson, Katharine Sherratt, Hamish P Gibbs, Nikos I Bosse, James D Munday, Sophie Meakin, Emma L Doughty, June Young Chun, et al. Estimating the time-varying reproduction number of sars-cov-2 using national and subnational case counts. *Wellcome Open Research*, 5(112):112, 2020.
