# Supplementary material for: Improving policy design and epidemic response using integrated models of economic choice and disease dynamics with behavioral feedback
Source: PLoS Comput Biol. 2025 Oct 3;21(10):e1013549. doi: 10.1371/journal.pcbi.1013549 (PMC12510651; doi:10.1371/journal.pcbi.1013549)
Supplement: Attachment — Submitted filename: rep-a-ploscb-PCOMPBIOL-D-25-00647.pdf [file pcbi.1013549.s002.pdf]

Comments on:

"Improving policy design and epidemic response using integrated models of economic choice and disease dynamics with behavioral feedback"

manuscript # PCOMPBIOL-D-25-00647 submitted to *PLOS Computational Biology*

To summarize this work, I use the authors' own description – "feedback-informed epidemiological model that integrates human behavior with disease dynamics" – because it so aptly captures the purpose of the paper. This paper does a lot, and does a great job of condensing the results to a small amount of journal space. To be specific, the authors develop and solve the dynamic model by picking reasonable values of the "free" parameters (calibration) in both the economic and epidemiological parts of the model. They then simulate solution to the calibrated model under four different policy scenarios (with differing values of some parameters within each scenario) and succinctly (as possible) report the results of each policy on health and employment outcomes. They then conduct a cost/benefit analysis (using a social welfare function calculation) to compare the different policies on this dimension and to determine optimal payments under different policy scenarios. Finally, they report impacts on different groups of people defined by vulnerability to disease and socioeconomic status. While the model is "simplified" in the sense that it doesn't capture some important aspects of the COVID-19 pandemic that impacted both work decisions and health outcomes (such as asymptomatic infection, the decision to test/vaccinate, school closings that might have necessitated non-employment, etc.), the model impressively demonstrates the connectedness of individual employment (economic) decisionmaking over time and the dynamic evolution of infection (health). While various extensions can be made to the current model, incorporating some of the very realistic ones will greatly complicate solution (and eventually estimation if good data are available) of the model.

In reviewing the submission, I read back and forth between the main paper and the supplementary appendix (SI.pdf) sections related to each section of the main paper. I decided to keep my specific comments (presented below) in the order of when I encountered them. Hence, the comments jump back and forth between the main paper and the appendix. Some comments suggest editorial/grammatical changes or clarifications; others are more substantive (but likely only require editorial responses).

Your model accounts for employment behavior, but not other types of health behaviors that might mitigate health impacts (e.g., exercising more or eating better) as well as offset the disutility of isolation requirements (e.g., outdoor exercising, getting a dog), although you do mention that the model could be extended to incorporate/consider these other behaviors (line 131). Generally, these omissions could be off-putting to a reader, but the authors do a good job of narrowing the scope of the model in a reasonable way and still demonstrating the advances that the dynamic economic behavior bring to the standard epidemiological model.

The model could similarly be applied to chronic disease detection and progression, as individual behaviors at different points in time (i.e., screening, follow up, treatment, adherence) affect longevity. Models of this type do exist in the literature and may be worth citing. Granted, these endogenous behaviors are each related to health, but could have economic impacts.

Line 36: Suggested revision of sentence: *Models help (who) to predict how infections spread and to compare control strategies.* The who might be policymakers, clinicians, individuals?

Line 79: I suggest changing "certain" to "particular" (as certain suggests 100% probability, which is not what you want to convey here)

Line 100: *One leverages macroeconomic models assuming non-infected individuals supply labor thereby contributing to aggregate output.*

Line 143: Your use of the terms "labor participation" and non-participation is a bit confusing. In economics, we use labor *force* participation (LFP), which is reported periodically as an important indicator of "health" of the economy at the federal level. However, it's not clear what your intention is: do you want to differentiate between working/employment that brings in income and not working (and therefore no income) OR do you want the choices to be working remotely versus going into the office (where the pecuniary impacts could be replaced with non-pecuniary costs such as isolation, lower productivity (perhaps), etc.). I'm guessing the former makes more sense here, since your tradeoff is money versus infection (wherein the latter could still be paid to work from home). If so, could the terms "Working" and "Not working" achieve your purposes? Or does that lose the aspect of "working for pay", which you might be trying to capture? Perhaps "employed" versus "non-employed". (It seems that you lean more to working and non working in the model description.) OR maybe I'm just "old school" and prefer labor force participation to labor participation. (Google AI suggests that labor participation is acceptable, although all references do point to LFP.)

Line 150: You haven't introduced what "infection and non-infection state variables" are, and whether you consider these to be endogenous variables or if you also want to include (treated as) exogenous variables (e.g., age, wage, family size, etc). The diagram suggests that the proportions of people in each infectious state (7 days ago) might be state variables, and one's current infectious state might be a state variable, but so too might be years of employment experience, underlying health state, savings, etc. These variables are introduced in the supplementary appendix (second paragraph), but a reader up to this point in the main paper will not know what a "state variable" means and what constitutes them.

In SI, p. 2, you say: "… individuals make decisions to maximize…". More correctly, the decisionmaking process IS the evaluation of different alternatives and selecting the alternative that maximizes lifetime utility. Hence, the "individual chooses among available alternatives to maximize…", and d_{it} is not the decision, but rather the alternative being evaluated.

In SI, p. 4, it might be worth mentioning that individuals are maximizing lifetime utility when you talk about "highest benefit". So, the benefit is not what they get today (only) but also the impact of today's

chosen alternative on future utility. You do use “lifetime” once but might be worth repeating when you use the term “benefits”.

Line 178-179: “*Potential costs of working for susceptible~~s~~ individuals …infected individuals who work incur~~s~~ costs…”* In addition to the suggested grammar changes in this sentence, you are also conflating future costs (in the first instance) with current costs (in the second). Perhaps you could say: “Potential costs of working for susceptible (currently non-infected) individuals is the increased probability of future loss of income and disutility of disease symptoms if one becomes infected, while infected individuals…”. However, these are future costs to the infected individuals as well, only with certainty until recovered. In other words, the costs in the first part of the sentence (for susceptibles) are also costs to infected folks.

Line 217: *Black curves in each panel show row outcomes using baseline parameters.* Also, unclear what Figure 3a and 3b (etc) refers to. Do you mean 3A (as in column A)? Perhaps use (Figure 3: column A) in the paragraph text.

Line 218: Your explanation of “utility cost of infection” appears to incorporate both non-pecuniary costs (symptoms) as well as pecuniary costs (medical care consumption). The latter, however, takes away from general consumption as well, but is not explicitly modeled because the model and the budget constraint does not include the decision to seek care or any exogenous cost of treatment if ill.

Why do you assume infinite horizon? In this decisionmaking model that specifically addresses health transitions, it makes sense to consider a finite horizon and the probability of death (which may depend on vulnerability and infection and, perhaps, also how long someone has been ill or how many times they have had the illness). This uncertain probability was a concern for many in the case of COVID-19 (even if subjectively incorrect/overstated).

In SI, p. 6, …*could also be over alternative beliefs an~~d~~ individual holds about…”*

In SI, p. 6, you haven’t defined Δt before introducing it in Equation 2. (I think you formally define it on p. 8, but it’s unclear if it’s meant to represent the frequency with which one can change employment status or if it’s meant to capture the lag in updating of infection probabilities.) It’s unclear why you can’t use “t+1” for the future period (in the Bellman equation), and explain that probabilities of infection in the future update with a 7-day (Δt?) lag. Technically, one is making an employment decision every period. Note also that you incorrectly subscript this delta (Δ_t)in the log part of Equation 4. Also, I see that you use t’ in lines 470-474 of the main text and make reference to a “timestep”.

In SI p. 7, …*The assumption that the unobserved states are i.d.d. (should be i.i.d., and you should probably indicate the abbreviation when you introduce it in third bullet point on p. 6)*

In SI p. 7, technically there are some ρ’s in equation 4 that you set to one, and this assumption impacts estimation of other parameters. But, no one (usually) estimates the ρ, so probably best to not introduce them.

In SI, p. 7, "*if the scenario calls ~~from~~ for tracking an individual's age…*"

In SI, p. 7, *For example, in a model that allows for financial savings, the amount of savings in the next period may depend on whether an individual earned labor income in the current period.* In this case, it's not "whether or not the individual earned income in current period" that is the state variable, but rather the stock of savings going into the next period (or coming into the current period).

In SI, p. 9, You could allow for an exogenous treatment cost if infected in the budget constraint (Equation 9). Don't epidemiologists think that treatment is potentially important to recovery and length of time contagious?

Considering recovery, you do not include "# of days ill" as a state variable. I'd assume that this variable would be important for predicting recovery (transition to the recovered state), even among epidemiologists. Why is it not included? (Perhaps you don't/won't see it in the data, but I'm guessing that it must be available or the epidemiologists couldn't predict recovery.) I see that the "$1/\gamma$" represents/defines the average duration of infection. Do durations of infection differ by risk group? (Yes, they do, as I now see in (11), where perhaps you could state in the table that g represents risk group.) Also, days recovered would be a state variable so that one knows when they are susceptible again (after 6 months, for example).

In SI, p. 9, "*We use log $c_{mt}(d_{mt})$ to indicate consumer risk aversion to lost consumption.*"

In SI, p. 10, "*We assume there is a baseline number of contacts each individual~~s~~ in the population would have…*"

In SI, p. 11, "*Note that ~~we~~ our formulation of the force of infection term in…*" What is "force of infection"? Oh, I see it is defined on Line 454 of main text.

The daily cost of infection ($5970, θ_i…and what about θ_v?) seems high relative to the hassle cost of working ($116). Granted, not working also results in lost income and therefore lost log(consumption), so perhaps these make sense together.

It's good that you considered the network aspects of contact by vulnerability and SES group (the only two measures of heterogeneity in your model). Could you include age in some way, given that older individuals are more susceptible and the ones who should isolate? I see that you do mention this omission on p. 4.

In SI, p. 14, "*…or through the use of fixed point acceleration algorithms.*"

In SI, p. 14, you should perhaps also mention that, in policy 1, the individuals who are forced to remain at home also are not paid (and that those not forced still choose whether to work or not).

In SI, p. 14, "*This transfer is added to the income the individual~~s~~ receives that period based on whether they work or not…*"

In SI, p. 17, "*From here we investigate how the model reacts when allowing the probability of getting infected to depend~~s~~ on the decision to work or not.*"

Line 235: *The impact of other parameters — such the time lag in individuals' information on population-level disease burden and additional utility cost of infection for vulnerable individuals — are shown in Figures S5 and S6.*

Line 249: Vulnearable is spelled incorrectly in legend of table.

Line 434: When discussing reasonable extensions, you might also want to mention that these examples not only need to include the additional costs that individuals might incur, but that they may also introduce additional decisions (such as use of take-home tests, masks, and vaccines), which would complicate solution considerably. Nonetheless, it's good to note that you need good data to estimate and identify these costs.

There are a few other grammatical errors that I didn't mention, but I'm sure the journal editor will catch those.

Lastly, I initially found the graphics to be difficult to follow, but once I absorbed the model content, I was able to understand the pictorial aspects of those figures. It's difficult to describe what you've done in so few pages. You've done a great job. Also, the appendix is very tightly explained, despite the complexity of the model.
