# Supplementary material for: Improving policy design and epidemic response using integrated models of economic choice and disease dynamics with behavioral feedback
Source: PLoS Comput Biol. 2025 Oct 3;21(10):e1013549. doi: 10.1371/journal.pcbi.1013549 (PMC12510651; doi:10.1371/journal.pcbi.1013549)
Supplement: Attachment — Submitted filename: Response_to_reviewers.pdf [file pcbi.1013549.s003.pdf]

Response to reviewer for manuscript #PCOMPBIOL-D-25-00647

The authors would like to thank the editor and reviewers for their constructive comments and suggestions that have helped improve the quality of this manuscript. The manuscript has undergone a thorough revision according to the editor and reviewers' comments. Please see below our responses. For the reviewers' convenience, we have highlighted significant changes in the revised manuscript in blue.

Response to the Editor

Editor Comment — Thank you for your submission, I and the reviewers were impressed by the work presented in your manuscript. While the reviewers were enthusiastic about the quality and potential significance of the research, they have identified a number of minor clarifications and adjustments to enhance the manuscript (including with some of the figures). I don't believe that any of these individually should be onerous; however, there are quite a few, so I encourage you to consider and respond to each carefully.

Reply: Thank you for your positive feedback and for overseeing the review process. We're pleased that you and the reviewers found the work compelling, and we appreciate the constructive suggestions. We are carefully addressing each of the comments raised. We agree that together they will strengthen the manuscript, and we are revising accordingly.

Response to the reviewers

Reviewer 1

This paper provides an interesting epidemiological-economic model for describing the spread of an infectious disease and a measure of agent economic stress. They employ an individual agent-based decision process to inform risk groups and couple this with a population level model for disease spread, with decisions and disease spread occurring over (potentially) different time steps. As the authors describe, this framework can allow for quite general models to inform future policy decision balancing health and wealth outcomes. The authors provide a nice example of how their framework might be used with decision makers to co-design policy – the biggest barrier to this as it stands is the authors' model is not trained or validated on real world data. I think this is a well written and presented paper, and if my few comments below are adequately addressed I wholeheartedly support it for publication.

Reviewer Comment 1.1 — I enjoyed the breadth of discussion in the introduction, but the novelty of the current work should be better identified. I suggest moving the paragraph in "Development of a feedback-informed epidemiological model" to the introduction to help this.

Reply: Thank you for your positive remarks about the Introduction. Based on your suggestion, we have we have moved the first paragraph from the Results, "Development of a feedback-informed

epidemiological model" section to the Introduction. In addition, we have now included a Methods section *before* the Results, which includes the remaining part of this former section, as well as the mathematical formulations which were previously in the "Materials and Methods" section at the very end of the last draft.

Reviewer Comment 1.2 — The model presented here is a novel proof of concept and has, for example, not been validated with real-world data. This point should be made clearer to the reader from the outset.

Reply: Thank you for pointing this out. We agree that the proof-of-concept nature of the model should be made clearer upfront. To address this, we have added the following sentence to the final paragraph of the new Methods section which immediately follows the Introduction:

"As a proof of concept, FIEM has not been fully validated against real-world data; instead, its parameters are sourced from previous studies grounded in empirical observations."

This clarification should help set appropriate expectations for the reader from the outset.

Reviewer Comment 1.3 — Figure 1 is hard to follow with a lot going on and not much guidance to assist parsing the information. I suggest reviewing this figure to see if a simpler alternative is possible; alternatively, add to Figure 1 to help with understanding.

Reply: We thank the reviewer for their thoughtful feedback regarding Figure 1. In response, we have introduced a new Figure 1 that provides a simplified, high-level conceptual overview of our approach to guide readers through the core idea of the model. Please see the new Figure 1 below:

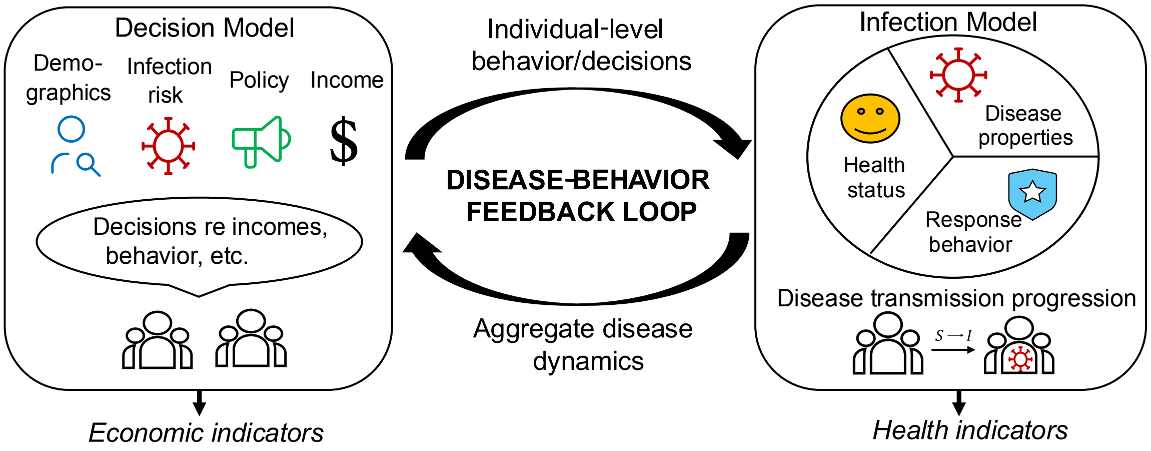

Figure 1: **Conceptual overview of the Feedback-Informed Epidemiological Model (FIEM).** The decision model simulates individual behaviors based on perceived disease dynamics, economic costs, policies, and demographics. These behaviors drive aggregate economic outputs and also disease dynamics. Concurrently, the infection model stratifies individuals into risk groups based on their decisions and individual factors, tracking disease transmission, progression, and recovery. The resulting disease dynamics again affect future individual behaviors and the course of the epidemic. A detailed visualization of each model component is presented in Figure 2.

The original, more detailed figure has been moved to Figure 2, where it now serves to support readers who wish to delve more deeply into the technical details of our method. We believe this restructuring improves the overall readability and flow of the paper, and we appreciate the reviewer's suggestion, which prompted this enhancement.

Reviewer Comment 1.4 — I am confused by Figure 2 B). This seems to show the average time infectious, which is presumed constant at approximately 7 days in the model description. However, across all scenarios depicted here, it is 8 days or more on average. This suggests some misspecification in the underlying model. Please address this.

Reply: Thank you for raising this point. In our configuration, the mean infectious period for a **single** infection event is set to 7 days. However, Figure 2B (Figure 3B in revised manuscript) reports the **cumulative** average number of days each individual spends in the infectious state over a **one-year SIRS** simulation, during which reinfections can occur. As a result, the total average exceeds 7 days. We use this cumulative metric to compare the relative magnitude of outbreaks across different model scenarios. We have added the following clarification to the caption of Figure 2:

"Note that panel B shows the cumulative average days each individual spends in the infectious state over a one-year SIRS simulation (allowing reinfections), so values can exceed the 7-day mean duration of a single infection event."

Reviewer Comment 1.5 — It would be interesting to see psychological components added to this model to investigate whether they have any potential influence on the agent's decision-making process. A paragraph on this in the discussion would be nice.

Reply: We agree that incorporating psychological factors and emotional costs could enhance the realism and scope of the model. To address these points, we have added a paragraph to the Discussion section that outlines how FIEM can be extended to include psychological components of decision-making. Please see revised manuscript lines 464 to 476 and below:

"Beyond rational expectations and economic trade-offs, FIEM can be extended to incorporate psychological and emotional dimensions of decision-making. For example, emotional costs such as social isolation, fear, or stigma can be modeled as additional non-monetary terms in the utility function. Individuals who isolate at home may experience a utility penalty, representing loneliness or reduced social contact, which can be estimated using survey-based measures of well-being [1]. Similarly, cognitive biases, such as misperceptions of infection risk, overconfidence, or time-inconsistent preferences, can be introduced by adjusting belief structures. For example, perceived infection risk could be modeled as a function of actual prevalence, incorporating bias parameters to reflect optimism or pessimism. These features are commonly explored in behavioral economics and psychology [2, 3] and can be integrated into FIEM's decision model without disrupting its core structure. "

Reviewer Comment 1.6 — Please provide references to support the claims in the first paragraph (p.1 ll.45-53).

Reply: Thank you for the suggestion. We have now added references to support the claims in the first paragraph, which emphasize the historical and contemporary importance of behavior in infectious disease transmission and control.

Reviewer Comment 1.7 — P.4 Section "Decision Scenario and parameterization". Please provided references and justification for the epidemiological and economic parameter values. For example, why assume $R_0 = 2.6$? Why assume a non-vulnerable individual is willing to pay $2000 per day to avoid infection?

Reply: Thank you for raising this point. The rationale for all epidemiological and economic parameter values used in our study is now provided in more detail in the Parameters section of the Supplementary Information, with a summary of the values included in Tables S1 and S2 for ease of reference.

Regarding the willingness to pay to avoid infection (cost of infection) for an individual, we note that the correct implied cost per infection in our model is ≈$5,700 (not $2,000 as stated mistakenly in one place in the original manuscript). This has been corrected in the revised manuscript (p. 13) and detailed in the SI (pages 13 - 14). There we review prior literature estimating this quantity for COVID-19 (e.g. [4, 5], and elaborate on the many reasons these estimates should be considered very rough estimates, and likely underestimates, based on prior studies of infectious disease outbreaks [6, 7]. We also detail how we calibrated out utility function parameters to recreate this implied value.

As for $R_0 = 2.6$, our goal was to have the model reproduce the characteristics of epidemic growth seen during early COVID-19 outbreaks, before interventions like social distancing or vaccination. We used a central value estimated in a large meta-analysis [8]. Note that we do not set R_0 value directly in the model, as R_0 is not an input parameter in epidemiological models. Rather, it is a quantity that arises from the combination of per-contact transmissibility (typically impossible to estimate directly), along with the duration of infectiousness, duration of immunity, and risk group specific contact matrices. We draw these latter three values from prior literature, and with their values fixed, we fit the probability of transmission per infectious contact (β) so that the model yields an R_0 around 2.6, estimated based on the logarithmic growth rate of infections during the first 20-day period. This is now described in more detail in the SI (Parameters section, and Table S1).

Reviewer Comment 1.8 — P.4 ll.184 (and throughout). Please specific the currency being used in the economic model. For example, is this in USD, AUD, NZD?

Reply: Thank you for pointing this out. We have clarified that all monetary values in the paper are denominated in U.S. dollars (USD) and have added a sentence to specify this in the main text. See revised manuscript lines 231:

"All monetary values in the paper are expressed in U.S. dollars (USD)."

Reviewer Comment 1.9 — P.6 ll.215 – What pre-defined sensitivity range?

Reply: In our sensitivity analysis, each parameter is varied from 60% to 140% of its baseline value (See the caption of Figure 3). The baseline values are presented in Appendix Tables S1 and S2.

Reviewer Comment 1.10 — In Figure 2, the baseline parameters show an average labor participation of 72%, but table 1 has this as 66.7%. Please explain why these are different.

Reply: Thank you for noticing this discrepancy. The difference arises from the time frames over which labor participation is measured. In Figure 2, the average labor participation (72%) is calculated over the full simulation period of 365 days. In contrast, the value reported in Table 1 (66.7%) reflects the average during the policy implementation period only (from day 20 to day 140). We have clarified this distinction in the caption of Table 1 by adding the following sentence:

All the values are calculated over the policy implementation period (day 20 to day 140).

Reviewer Comment 1.11 — P.15. In the specification of Equation (2) it seems the variable z_{mt} is unnecessary and just adds confusion, as this only appears implicitly in the indicator i_{mt}. I would suggest revising this for clarity.

Reply: Thank you for this helpful comment. We agree that the components of z_{mt} are shown explicitly in Equation (2). However, we define z_{mt} as the full state of the individual, including infection status, socioeconomic status, and vulnerability. We've updated the text around Equation (2) to clarify this and to explain why we retain z_{mt} as a compact notation for the individual's risk and health profile. See added sentences below:

"In this function, the state vector z includes individuals' infection status (e.g., susceptible), socioeconomic status (e.g., high-SES), and vulnerability status (e.g., non-vulnerable). Although the components of z appear explicitly in the utility expression, we retain z as a shorthand to represent the individual's full risk and health profile. A detailed formulation and explanation of these variables is provided in the Supplementary Materials Scenario section."

Reviewer Comment 1.12 — Throughout, the authors have used the utility function as a measure of well-being, but this utility fails to account for many psychological aspects of well-being (e.g. https://doi.org/10.5502/ijw.v13i4.3665). I suggest rephrasing to "financial well-being" or similar as this is what is more accurately captured in their utility function.

Reply: Thank you for raising this point. You rightly point out that the parameterization of the FIEM that we feature in the text has a utility function that does not directly capture psychological aspects of well-being. It does capture some nonfinancial measures of well-being such as an individual's health state, which can adversely affect well-being through nonfinancial channels. However, we agree that it is important to make it clearer how FIEM could be extended further to capture these additional channels for measuring well-being which is why we have added the following text to the Discussion section (see page 15):

Beyond rational expectations and economic trade-offs, FIEM can be extended to incorporate psychological and emotional dimensions of decision-making, capturing a broader sense of well-being than just health or financial. For example, emotional costs such as social isolation, fear, or stigma can be modeled as additional non-monetary terms in the utility function. Individuals who isolate at home may experience a utility penalty, representing loneliness or reduced social contact, which can be estimated using survey-based measures of well-being [1]. Similarly, cognitive biases, such as misperceptions of infection risk, overconfidence, or time-inconsistent preferences, can be introduced by adjusting belief structures. For example, perceived infection risk could be modeled as a function of actual prevalence, incorporating bias parameters to reflect optimism or pessimism. These features are commonly explored in behavioral economics and psychology [2,3] and can be integrated into FIEM's decision model without disrupting its core structure.

Reviewer Comment 1.13 — P.1 ll.57 spell out Susceptible-Infectious-Recovered (SIR) the first time it appears.

Reply: Thank you for pointing this out. We have spelled out Susceptible-Infectious-Recovered (SIR) at its first mention on page 1.

Reviewer Comment 1.14 — P.7 l.226 – some punctuation is missing, i.e. "...labor supply of susceptible individuals infected individuals..."

Reply: Thank you for catching this. We have corrected the sentence to include the missing punctuation.

Reviewer Comment 1.15 — Figure 5: in the caption, the authors say that labor restrictions goes from red to yellow, but the figure shows this as yellow to red.

Reply: Thank you for pointing this out. We have revised the caption as "(yellow to red)".

Reviewer Comment 1.16 — Figure 5: What is the star in E and F?

Reply: The star markers in panels E and F of Figure 5 indicate the baseline model scenario with no intervention. We have updated the figure caption to clarify this: "The black curves and stars are the baseline model with no intervention"

Reviewer Comment 1.17 — Figure 6 – In the caption when defining U_C, the cost utility should be an upper case C.

Reply: Thank you for capturing this, we have corrected this in the caption.

Reviewer Comment 1.18 — Figure 7 – It should be a capital B) rather than b).

Reply: Thank you for capturing this, we have corrected this in the caption.

Reviewer Comment 1.19 — P.15 l.468 there should be a space between "and" and θ_v.

Reply: Thank you for noting this. We have added the missing space.

Reviewer 2

To summarize this work, I use the authors' own description – "feedback-informed epidemiological model that integrates human behavior with disease dynamics" – because it so aptly captures the purpose of the paper. This paper does a lot, and does a great job of condensing the results to a small amount of journal space. To be specific, the authors develop and solve the dynamic model by picking reasonable values of the "free" parameters (calibration) in both the economic and epidemiological parts of the model. They then simulate solution to the calibrated model under four different policy scenarios (with differing values of some parameters within each scenario) and succinctly (as possible) report the results of each policy on health and employment outcomes. They then conduct a cost/benefit analysis (using a social welfare function calculation) to compare the different policies on this dimension and to determine optimal payments under different policy scenarios. Finally, they report impacts on different groups of people defined by vulnerability to disease and socioeconomic status. While the model is "simplified" in the sense that it doesn't capture some important aspects of the COVID-19 pandemic that impacted both work decisions and health outcomes (such

as asymptomatic infection, the decision to test/vaccinate, school closings that might have necessitated non-employment, etc.), the model impressively demonstrates the connectedness of individual employment (economic) decisionmaking over time and the dynamic evolution of infection (health). While various extensions can be made to the current model, incorporating some of the very realistic ones will greatly complicate solution (and eventually estimation if good data are available) of the model.

Reviewer Comment 2.1 — In reviewing the submission, I read back and forth between the main paper and the supplementary appendix (SI.pdf) sections related to each section of the main paper. I decided to keep my specific comments (presented below) in the order of when I encountered them. Hence, the comments jump back and forth between the main paper and the appendix. Some comments suggest editorial/grammatical changes or clarifications; others are more substantive (but likely only require editorial responses).

Reply: Thank you for the thorough review. We appreciate the time you took to engage with both the main manuscript and the supplementary appendix. We understand the back-and-forth structure of your comments and have addressed each point in the order presented to ensure nothing was overlooked. We have made revisions and clarifications where appropriate and hope our responses adequately address your concerns.

Reviewer Comment 2.2 — Your model accounts for employment behavior, but not other types of health behaviors that might mitigate health impacts (e.g., exercising more or eating better) as well as offset the disutility of isolation requirements (e.g., outdoor exercising, getting a dog), although you do mention that the model could be extended to incorporate/consider these other behaviors (line 131). Generally, these omissions could be off-putting to a reader, but the authors do a good job of narrowing the scope of the model in a reasonable way and still demonstrating the advances that the dynamic economic behavior bring to the standard epidemiological model.

Reply: Thank you for the comment. We chose to focus on the decision to engage in in-person work because there is both a clear mechanism and strong empirical evidence for the association between employment behavior and disease risk. We believe that having this simple, binary decision with immediate impact on risk was a useful proof-of-concept scenario to demonstrate how dynamic economic decision-making can shape disease dynamics. We intentionally narrowed the scope to highlight this mechanism clearly while designing the framework to be flexible and extensible. The model can be readily adapted to incorporate other health-related behaviors or coping strategies in future applications or settings. We appreciate your recognition that the model advances epidemiological modeling even within its current scope.

Reviewer Comment 2.3 — The model could similarly be applied to chronic disease detection and progression, as individual behaviors at different points in time (i.e., screening, follow up, treatment, adherence) affect longevity. Models of this type do exist in the literature and may be worth citing. Granted, these endogenous behaviors are each related to health, but could have economic impacts.

Reply: We appreciate the reviewer's suggestion. FIEM is indeed flexible enough to be applied to chronic disease settings, where individual behaviors such as screening, treatment adherence, and lifestyle changes significantly influence long-term health and economic outcomes. The framework's ability to capture dynamic, forward-looking behavioral choices makes it well-suited for these contexts.

That said, this study is specifically focused on infectious diseases, where individual behavior has both individual and population-level consequences due to transmission externalities. Capturing this behavioral-epidemiological feedback is the core innovation we aim to highlight. We hope to explore chronic disease applications in future work and thank the reviewer for encouraging this broader perspective.

Reviewer Comment 2.4 — Line 36: Suggested revision of sentence: Models help (who) to predict how infections spread and to compare control strategies. The who might be policymakers, clinicians, individuals?

Reply: Thank you for the helpful suggestion. We have revised the sentence to clarify the subject. It now reads:

"Models help researchers and policymakers predict how infections spread and compare control strategies."

Reviewer Comment 2.5 — Line 79: I suggest changing "certain" to "particular" (as certain suggests 100% probability, which is not what you want to convey here)

Reply: Thank you for the suggestion. We agree that "particular" is more appropriate in this context and have updated the text accordingly.

Reviewer Comment 2.6 — Line 100: One leverages macroeconomic models assuming non-infected individuals supply labor thereby contributing to aggregate output.

Reply: Thank you for the suggestion. We have revised the sentence accordingly.

Reviewer Comment 2.7 — Line 143: Your use of the terms "labor participation" and non-participation is a bit confusing. In economics, we use labor force participation (LFP), which is reported periodically as an important indicator of "health" of the economy at the federal level. However, it's not clear what your intention is: do you want to differentiate between working/employment that brings in income and not working (and therefore no income) OR do you want the choices to be working remotely versus going into the office (where the pecuniary impacts could be replaced with non-pecuniary costs such as isolation, lower productivity (perhaps), etc.). I'm guessing the former makes more sense here, since your tradeoff is money versus infection (wherein the latter could still be paid to work from home). If so, could the terms "Working" and "Not working" achieve your purposes? Or does that lose the aspect of "working for pay", which you might be trying to capture? Perhaps "employed" versus "non-employed". (It seems that you lean more to working and non working in the model description.) OR maybe I'm just "old school" and prefer labor force participation to labor participation. (Google AI suggests that labor participation is acceptable, although all references do point to LFP.)

Reply:

Thank you for this comment. Our primary intention was to differentiate working vs not-working to create a tradeoff between earning income/consumption and infection risks. Thus we have updated our terminology throughout the paper to reflect "working" or "labor supply" as opposed to "labor participation" to improve clarity.

Reviewer Comment 2.8 — Line 150: You haven't introduced what "infection and non-infection state variables" are, and whether you consider these to be endogenous variables or if you also want

to include (treated as) exogenous variables (e.g., age, wage, family size, etc). The diagram suggests that the proportions of people in each infectious state (7 days ago) might be state variables, and one's current infectious state might be a state variable, but so too might be years of employment experience, underlying health state, savings, etc. These variables are introduced in the supplementary appendix (second paragraph), but a reader up to this point in the main paper will not know what a "state variable" means and what constitutes them.

Reply: Thank you for the comment. We agree that the original text did not clearly define what constitutes "infection" and "non-infection" state variables, which may cause confusion for readers unfamiliar with the modeling framework. To address this, we have revised the sentence at revised manuscript line 133 in the main text to briefly clarify these terms:

"Our framework classifies individuals based on their infection state variables, such as time-varying infection status (e.g., susceptible, infectious) and non-infection state variables, which include decision status (e.g., choice to work or engage in social distancing), as well as by a set of other state variables that may be fixed or time-dependent (e.g., demographics, health vulnerability, socioeconomic profile)."

Reviewer Comment 2.9 — In SI, p. 2, you say: "... individuals make decisions to maximize...". More correctly, the decision making process IS the evaluation of different alternatives and selecting the alternative that maximizes lifetime utility. Hence, the "individual chooses among available alternatives to maximize...", and d_{it} is not the decision, but rather the alternative being evaluated.

Reply: Thank you for pointing out this potential source of confusion. This section is only meant to summarize the model, and details of the decision making process are provided in a later section (SI page 5). There we clarify that the notation d^*_{mt} is reserved for the actual decision taken, while we use d more generally to refer to the alternatives. We have revised the wording in the SI to make it more clear that utility can be evaluated for any alternative decision and that evaluation is used to make the decision:

"Utility ($u(\cdot)$) is a function of observable-state variables summarized by z, a decision an individual could make denoted by d, state variables unobserved to the modeler but known to the individual ϵ and a vector of utility model parameters Θ. Each period, individuals choose among available alternative decisions to maximize the present discounted value of their lifetime flow utilities ... "

Reviewer Comment 2.10 — In SI, p. 4, it might be worth mentioning that individuals are maximizing lifetime utility when you talk about "highest benefit". So, the benefit is not what they get today (only) but also the impact of today's chosen alternative on future utility. You do use "lifetime" once but might be worth repeating when you use the term "benefits".

Reply: Thank you for this suggestion. We agree that it is important to reinforce that "benefits" in our model refer to lifetime utility, not just immediate utility. We have updated the SI to clarify this point:

"A decision is optimal if it is associated with the highest benefits—measured in terms of expected lifetime utility, where these benefits are measured by a value function—to an individual at that time."

Reviewer Comment 2.11 — Line 178-179: "Potential costs of working for susceptibl~~es~~ individuals ...infected individuals who work incur~~s~~ costs..." In addition to the suggested grammar changes in this sentence, you are also conflating future costs (in the first instance) with current costs (in the second). Perhaps you could say: "Potential costs of working for susceptible (currently non-infected) individuals is the increased probability of future loss of income and disutility of disease

symptoms if one becomes infected, while infected individuals...". However, these are future costs to the infected individuals as well, only with certainty until recovered. In other words, the costs in the first part of the sentence (for susceptibles) are also costs to infected folks.

Reply: We agree with both the grammatical corrections and the need to clarify the distinction between the types of costs faced by susceptible and infected individuals. We have revised the sentence accordingly to better reflect that susceptible individuals face the probabilistic risk of future infection, and thus potential income loss and health disutility, while infected individuals face more immediate and certain ongoing costs, such as discomfort and social stigma, until they recover.

Please see revised manuscript, line 223:

"Potential costs of working for susceptible individuals (currently uninfected) are the increased probability of future loss of income and disutility of disease symptoms if one becomes infected, while infected individuals who work incur ongoing costs such as discomfort of working while sick or stigma associated with being infectious."

Reviewer Comment 2.12 — Line 217: Black curves in each panel show row outcomes using baseline parameter~~s~~. Also, unclear what Figure 3a and 3b (etc) refers to. Do you mean 3A (as in column A)? Perhaps use (Figure 3: column A) in the paragraph text.

Reply: Thank you for pointing this out. We have revised the sentence for clarity. We have also updated all figure panel references to use uppercase labels.

Reviewer Comment 2.13 — Line 218: Your explanation of "utility cost of infection" appears to incorporate both non-pecuniary costs (symptoms) as well as pecuniary costs (medical care consumption). The latter, however, takes away from general consumption as well, but is not explicitly modeled because the model and the budget constraint does not include the decision to seek care or any exogenous cost of treatment if ill.

Reply: Thank you for making this observation. You are correct that a richer model would capture some costs of illness by directly reducing consumption as opposed to loading those costs onto a preference parameter that captures the utility costs of illness. The model presented in the paper is intended to be illustrative of how our framework can be used to capture the different types of tradeoffs individuals face when making decisions during a pandemic scenario. The core tradeoffs in our stylized model and a model where illness lower consumption are fundamentally the same so they are unlikely to diverge in their predictions of how choices will respond to the counterfactual policies we simulate. We have added the following sentence to clarify this point in the text (see page 7):

While this set-up limits the impact of infection to arise through the utility function and not other channels such as reduced consumption because an individual needs to seek out and pay for medical care, it preserves our ability to assess the core health-wealth tradeoff that motivates our scenario.

Reviewer Comment 2.14 — Why do you assume infinite horizon? In this decision making model that specifically addresses health transitions, it makes sense to consider a finite horizon and the probability of death (which may depend on vulnerability and infection and, perhaps, also how long someone has been ill or how many times they have had the illness). This uncertain probability was a concern for many in the case of COVID-19 (even if subjectively incorrect/overstated).

Reply: We thank the reviewer for raising this important point. In our framework, adopting an infinite-horizon formulation serves three main purposes:

- **Alignment with policy-making objectives:** An infinite-horizon approach reflects the needs of policymakers, who typically seek sustainable guidance rather than rules tied to a fixed end date. In real-world settings, policy decisions must remain valid as conditions evolve. Optimizing over an infinite horizon supports this by identifying decision rules that are robust and applicable over time, rather than being constrained by an arbitrary period.
- **Discounting down weights far-future uncertainty:** Although the model runs indefinitely, we apply a discount factor k, which reduces the influence of outcomes in the distant future. This means that any uncertainty related to long-run outcomes is given negligible weight in today's decisions. This aligns with how individuals typically behave: the immediate trade-offs (e.g., whether to work while infected) dominate decision-making, while events far in the future carry little practical weight.
- **Computational tractability:** We adopt an infinite-horizon formulation to leverage Rust's nested fixed-point method for solving dynamic discrete choice problems. This approach allows us to compute a stationary value function using fixed-point iteration, and in our setting, it yields a closed-form solution for the value function. This eliminates the need to specify a terminal value function, as would be required in a finite-horizon model, and avoids introducing assumptions about end-of-horizon outcomes such as long-term health or mortality effects.

It is possible to model death in a model with an infinite horizon if it is treated as an absorbing state with a utility payoff of zero in perpetuity. Incorporating this feature would not alter the three advantages we identified in our prior response. While omitting death and the infinite horizon are simplifications, relaxing them would not fundamentally alter the core health-wealth tradeoffs featured in our scenario simulations. In fact, these features may increase the strength of this tradeoff. For example, a finite horizon may introduce stronger pre-cautionary incentives for low income individuals to continue working. Exploring these differences and behaviors would be an interesting area for future work to explore.

Reviewer Comment 2.15 — In SI, p.6, …could also be over alternative beliefs an~~d~~ individual holds about…"

Reply: Thank you for catching this. We have corrected the sentence.

Reviewer Comment 2.16 — In SI, p.6, you haven't defined Δ_t before introducing it in Equation 2. (I think you formally define it on p. 8, but it's unclear if it's meant to represent the frequency with which one can change employment status or if it's meant to capture the lag in updating of infection probabilities.) It's unclear why you can't use "$t+1$" for the future period (in the Bellman equation), and explain that probabilities of infection in the future update with a 7-day (Δ_t?) lag. Technically, one is making an employment decision every period. Note also that you incorrectly subscript this delta (Δ_t)in the log part of Equation 4. Also, I see that you use t' in lines 470-474 of the main text and make reference to a "timestep".

Reply:

Thank you for noticing this source of confusion. We use Δt to describe the time interval at which individuals update their decisions. You are correct that decisions occur in discrete steps, but we have avoided more typical "$t+1$" notation as it tends to imply that updates must be daily (or some other unit, like weekly), when in reality they can be arbitrary. The epidemiological model variables may update

many times - and could even be represented in continuous time - between discrete timepoints at which decisions are made, so we wanted to move away from the suggestion that only discrete time periods are considered in the model. We have now updated the SI ("Decision Model" section, immediately after Equation 2) to clarify this:

where Δt denotes the time interval of arbitrary duration between times when individuals update their decisions

Thank you for catching the indexing typos. We have fixed that throughout the SI and the main text.

Reviewer Comment 2.17 — In SI p.7, . . . The assumption that the unobserved states are i.d.d. (should be i.i.d., and you should probably indicate the abbreviation when you introduce it in third bullet point on p. 6)

Reply: Thank you for catching this. We have corrected the typo from "i.d.d." to "i.i.d." (independent and identically distributed) and now define the abbreviation where it first appears in the third bullet point on p.6 for clarity.

Reviewer Comment 2.18 — In SI p.7, technically there are some ρ's in equation 4 that you set to one, and this assumption impacts estimation of other parameters. But, no one (usually) estimates the ρ, so probably best to not introduce them.

Reply: You are right that in models with extreme value-distributed unobserved utilities, a scale parameter ρ typically governs the variance of the error term and appears in the log-sum expression. Since we do not estimate or vary ρ, we have intentionally chosen not to introduce it in the notation to maintain clarity. We appreciate the opportunity to clarify this modeling choice.

Reviewer Comment 2.19 — In SI, p. 7, "if the scenario calls ~~from~~ for tracking an individual's age. . . "

Reply: Thank you for noting this. We have corrected the sentence.

Reviewer Comment 2.20 — In SI, p.7, For example, in a model that allows for financial savings, the amount of savings in the next period may depend on whether an individual earned labor income in the current period. In this case, it's not "whether or not the individual earned income in current period" that is the state variable, but rather the stock of savings going into the next period (or coming into the current period).

Reply:

Thank you for raising this point. You are correct that a model with savings would track that stock as a separate state variable. We have modified the text on SI page 7 to read as:

For example, in a model that allows for financial savings, the amount of savings in the next period depends on how the stock savings in the current period changed (i.e., all new income is consumed, some new income is saved, savings are used to smooth consumption, etc.).

Reviewer Comment 2.21 — In SI, p.9, You could allow for an exogenous treatment cost if infected in the budget constraint (Equation 9). Don't epidemiologists think that treatment is potentially important to recovery and length of time contagious?

Reply: Thank you for the suggestion. We agree that including an exogenous treatment cost for infected individuals, and allowing treatment to influence the duration of infectiousness and/or the severity of symptoms, could be a valuable extension for certain applications. For the specific example scenario considered here - COVID-19 in 2020 - it was not necessary, as effective antiviral medications (i.e. paxlovid) did not become widely available until 2023. We view this as a natural direction for future work and note that the FIEM framework is flexible enough to accommodate such additions.

Reviewer Comment 2.22 — Considering recovery, you do not include "number of days ill" as a state variable. I'd assume that this variable would be important for predicting recovery (transition to the recovered state), even among epidemiologists. Why is it not included? (Perhaps you don't/won't see it in the data, but I'm guessing that it must be available or the epidemiologists couldn't predict recovery.) I see that the "$\frac{1}{\gamma}$" represents/defines the average duration of infection. Do durations of infection differ by risk group? (Yes, they do, as I now see in (11), where perhaps you could state in the table that g represents risk group.) Also, days recovered would be a state variable so that one knows when they are susceptible again (after 6 months, for example).

Reply: Thank you for the comment. In the current model, we adopt a discrete-time Markov process to govern disease progression, which assumes memoryless transitions between states. That is, the probability of recovery for an infected individual in each timestep is fixed, regardless of how long they have already been infected. The duration of infection for each individual is therefore an exponentially-distributed random variable with expected value γ. This structure does not require us to explicitly track the number of days an individual has been infected. However, if we wanted to increase the complexity of our model to include arbitrary distributions for the duration of infectiousness or more complex profiles of waning immunity (which is more realistic, and commonly done in infectious disease modeling), then these durations could be included as state variables. This would increase the dimensionality of the state space, but the FIEM framework is well-suited to support such extensions if needed for a specific application.

We also confirm that our current model specification allows group-specific disease parameters. As described in Equation (11) of SI, we use a subscript g to denote each transition probability (e.g., recovery, infection), highlighting that these dynamics and parameters can vary by group. To improve clarity, we have revised the text preceding Equation (11). The updated sentence now reads:

"Susceptible individuals in risk group g can acquire infection from infected individuals in any risk group, at a rate that depends on their propensity to make contacts with individuals in corresponding risk group g_2 ($\mathbb{C}_{g,g_2}$) and the per-contact rate of disease transmission (β). Infected individuals immediately become infectious, recover from infection and lose the ability to transmit at a rate γ_g, leading to an average duration of infection of $1/\gamma_g$. Recovered individuals are immune to re-infection, but lose immunity and become susceptible again with a waning rate α_g (where $1/\alpha_g$ is the average duration of protection) ... "

Reviewer Comment 2.23 — In SI, p.11, "Note that ~~we~~ our formulation of the force of infection term in..." What is "force of infection"? Oh, I see it is defined on Line 454 of main text.

Reply: Thank you for pointing this out. We now define force of infection a few paragraphs before this in the SI, and also include the definition again in this sentence for clarity.

Reviewer Comment 2.24 — The daily cost of infection (\$5970, θ_x... and what about θ_v?)

seems high relative to the hassle cost of working ($116). Granted, not working also results in lost income and therefore lost log(consumption), so perhaps these make sense together.

Reply:

We have now expanded the section of the Supplementary Information ('Parameters' section, p. 13–14) where we review the relevant literature on estimates of the value per statistical case of COVID-19 (a measure of individuals' willingness to pay to avoid infection). We justify using an approximate value of $6,000 and calibrating our utility function to recreate this value in our simulations.

We have also added more detail regarding the hassle costs. The hassle costs are calibrated to be moderate in size such that they can generate variation in the decisions that otherwise-identical individuals make each period (as a proxy for variation due to unmodeled variables), which can only be partially accomplished by the reduction in consumption from not working. Richer models informed by data may break up these costs into different sources and allow for more heterogeneity in their values across the population.

The daily cost of infection is indeed much higher than the hassle cost of working, but a direct comparison is not particularly relevant, because hassle costs are *always* incurred when working, while infection at working typically only occurs with a low probability - it requires exposure to an infected individual at work and then for transmission to probabilistically occur.

Reviewer Comment 2.25 — It's good that you considered the network aspects of contact by vulnerability and SES group (the only two measures of heterogeneity in your model). Could you include age in some way, given that older individuals are more susceptible and the ones who should isolate? I see that you do mention this omission on p. 4.

Reply: Thank you for the comment. We agree that incorporating age stratification could enrich the model, since age has been associated with multiple aspects of COVID-19 infection, including vulnerability to severe disease and contact patterns. We have incorporated age stratification into many of our own detailed epidemiological models. For the purpose of this paper, we tried to keep the epidemiological model simple and did not explicitly include age. However, even in our current scenario, age is partially captured through the vulnerability state variable: individuals classified as vulnerable—who face higher infection costs and thus exhibit greater willingness to isolate—could be interpreted as representing older populations. The flexibility of our model means that future work could extend it to include age as an additional state variable, with, for example, a more detailed representation of interaction patterns, health risks, and utility trade-offs by age group.

Reviewer Comment 2.26 — In SI, p.14, "...or through the use of fixed point acceleration algorithms."

Reply: Thank you for catching this grammar error. We have revised the sentence.

Reviewer Comment 2.27 — In SI, p.14, you should perhaps also mention that, in policy 1, the individuals who are forced to remain at home also are not paid (and that those not forced still choose whether to work or not).

Reply: We have revised the description of Policy 1 in the Supplementary Information to clarify that individuals forced to stay home also do not receive wages, while those not restricted retain the ability to choose whether to work.

"Under this policy, a randomly chosen share of the population is forced to remain at home and does not receive wages, while the other portion of the population can choose to work or not."

Reviewer Comment 2.28 — In SI, p. 14, "This transfer is added to the income the individual~~s~~ receives that period based on whether they work or not. . . "

Reply: Thank you for catching this. We have revised the sentence accordingly.

Reviewer Comment 2.29 — In SI, p. 17, "From here we investigate how the model reacts when allowing the probability of getting infected to depend~~s~~ on the decision to work or not."

Reply: Thank you for catching this. We have revised the sentence accordingly.

Reviewer Comment 2.30 — Line 235: The impact of other parameters — such the time lag in individuals' information on population-level disease burden and additional utility cost of infection for vulnerable individuals — are shown in Figures S5 and S6.

Reply: Thanks for pointing this out, we have added — to the sentence.

Reviewer Comment 2.31 — Line 249: Vulne~~a~~rable is spelled incorrectly in legend of table.

Reply: We have corrected the caption for the figure, please see below:

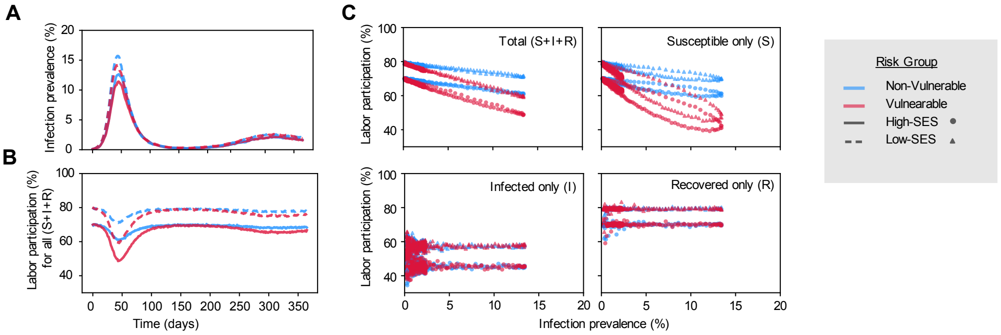

Reviewer Comment 2.32 — Line 434: When discussing reasonable extensions, you might also want to mention that these examples not only need to include the additional costs that individuals might incur, but that they may also introduce additional decisions (such as use of take-home tests, masks, and vaccines), which would complicate solution considerably. Nonetheless, it's good to note that you need good data to estimate and identify these costs.

Reply: We agree, and intended for that paragraph to convey that. We have updated the wording slightly now to make it more clear that we are referring to the addition decisions these interventions introduce:

"The decision to abstain from work to avoid infection was particularly salient during the early phase of COVID-19, when rapid at-home tests, medical-grade face-masks, and vaccines were unavailable. Our model could be extended to consider the additional decisions processes individuals engage in to utilize these interventions, ..."

We also emphasize the importance of having appropriate data to calibrate or estimate the behavioral responses associated with such extensions. Relevant datasets are further discussed in our responses to Comment 3.1, Comment 3.3, and Comment 3.7.

Reviewer Comment 2.33 — There are a few other grammatical errors that I didn't mention, but I'm sure the journal editor will catch those.

Reply: We thank the reviewer for bringing this to our attention. We have carefully proofread the manuscript and corrected several grammatical issues throughout the paper to improve clarity and readability.

Reviewer Comment 2.34 — Lastly, I initially found the graphics to be difficult to follow, but once I absorbed the model content, I was able to understand the pictorial aspects of those figures. It's difficult to describe what you've done in so few pages. You've done a great job. Also, the appendix is very tightly explained, despite the complexity of the model.

Reply: Thank you for the kind words and feedback. We're glad the figures and appendix became clear as the model unfolded, and we appreciate your recognition of the effort to present this work.

Reviewer 3

This is a potentially important attempt to develop behavioural-epi models; the authors couple an SIRS model for infection with waning immunity, to a dynamic model for decision to work during a pandemic. Decisions to work are assumed rational, and exponentially discounted, derived from perfect knowledge of individual's own infection status and global prevalence, with risks and benefits of work stratified by socio-economic status, employment status and vulnerability.

Reviewer Comment 3.1 — It is great to see an attempt to capture heterogeneities in baseline behaviour and behavioural choice during a pandemic. The risk categories within the model are well-motivated in order to enable consideration of a range of potential policy decisions around work during a pandemic. I can see that even this epidemiologically simple (but overall complex) model, could be useful as a tool to communicate trade-offs to policy makers. Regarding the population stratification, do data to parameterise these risk groups exist for most countries? If so that may be something to emphasise as a strength of this approach.

Reply: Thank you for your feedback. We agree that stratifying the population by socioeconomic status (SES) and vulnerability is key to evaluating targeted policy responses, and we appreciate your point about data availability. While the specific SES and health risk factors that are relevant to decision-making may be different for new emerging infections, experiences during COVID-19 suggest that data on these factors can be collected quickly. For example, in spring 2020, the COVID-19 Impact Survey [9] was rapidly designed and implemented to collect nationally representative data across the U.S. regarding work decisions, income levels (for SES stratification), and pre-existing health conditions (which can proxy for vulnerability). The iCARE study is an ongoing multi-year international effort to collect data on risk factors and health behaviors for COVID-19 and beyond [10]. Many other such efforts exist or are in development and are now considered a crucial component of pandemic preparedness.

We have added the following sentence to the Discussion to highlight this data availability:

"Surveys like the COVID-19 Impact Survey [9], which captured income, employment, and health data at the individual level across the U.S. in spring 2020, show that relevant risk group parameters can be estimated rapidly during an outbreak."

Reviewer Comment 3.2 — The focus in construction of the utility function is economic costs, have the authors considered modelling the emotional cost of lack of social contact? Would this be possible in this framework?

Reply: Thank you for the question. Yes, modeling the emotional cost of reduced social contact is possible within the FIEM framework. We have addressed this point more broadly in our response to Comment 1.5 and have added a corresponding paragraph in the revised Discussion section:

Beyond rational expectations and economic trade-offs, FIEM can be extended to incorporate psychological and emotional dimensions of decision-making, capturing a broader sense of well-being than just health or financial. For example, emotional costs such as social isolation, fear, or stigma can be modeled as additional non-monetary terms in the utility function. Individuals who isolate at home may experience a utility penalty, representing loneliness or reduced social contact, which can be estimated using survey-based measures of well-being [1]. Similarly, cognitive biases, such as misperceptions of infection risk, overconfidence, or time-inconsistent preferences, can be introduced by adjusting belief structures. For example, perceived infection risk could be modeled as a function of actual prevalence, incorporating bias parameters to reflect optimism or pessimism. These features are commonly explored in behavioral economics and psychology [2, 3] and can be integrated into FIEM's decision model without disrupting its core structure. These examples highlight how FIEM can use dynamic optimization with additional choice frictions to better approximate the true decision making process and align with other research on the complex determinants of human behavior.

Reviewer Comment 3.3 — While the model allows for 8 different categories of agent, with potential for heterogeneous mixing between these strata, the epidemiological model is potentially overly simplistic for a pathogen like SARS-CoV-2: latent and asymptomatic infectious states would generally be considered crucial for describing SARS-CoV-2 transmission, and calibrating using data streams such as reported infection, and indeed realistically capturing **knowledge** about an agent's infectious state. I can see this is partially discussed in the paragraph beginning line 410, but I'm unsure how you expect this to affect your results? Are the computational costs involved in extending the model prohibitive?

Reply: Thank you for this important comment. We agree that the latent and asymptomatic infectious states, as well as the assumption of perfect knowledge of infection status, are important factors to consider for modeling COVID-19.

Regarding adding more infection states: In our current framework, the computational burden primarily arises from solving the dynamic decision model separately for each risk group. The number of risk groups in our current setup is 8, determined by combinations of SES (high/low), vulnerability (vulnerable/non-vulnerable), and work decision (work/not work). Adding additional infection states (e.g., latent or asymptomatic) does not significantly increase computational cost, because all infection states can be handled within a nested Bellman equation for a given risk group. We acknowledge that including additional infection states may impact simulation results, particularly underestimating presymptomatic transmission (which is less likely to be reduced by behavioral interventions). We have

noted this as a limitation and a direction for future work, and we are currently working on extending FIEM to include more detailed infection progression states.

Regarding capturing imperfect knowledge of infection status: FIEM can be extended to capture imperfect information. The scenario considered in this paper already includes one simple form of imperfect knowledge by assuming a 1-week lag in reports (and thus knowledge) of the aggregate infection rate. Additional types of information friction that can be incorporated into FIEM include an individual not knowing their own true infection state or having biased beliefs about the probability they will become infected. These extensions require tracking additional state variables or integrating over distributions to solve the model, which increases the computational burden. A related challenge to incorporating some of these features is that they require data on individual beliefs. Some data sources collected during the COVID-19 time period (see e.g., Six-Country Survey on COVID-19) track this information and could be incorporated as part of future work extending FIEM.

We have also added the following clarifying sentence to the Discussion section (see revised manuscript lines 478 to 481):

"This includes omitting explicit tracking of pre-symptomatic and asymptomatic infectious states, which are particularly important for accurately capturing SARS-CoV-2 transmission dynamics and act to limit individuals' knowledge of their own infectious status."

Reviewer Comment 3.4 — The models for individual behavioural choice are rooted in economic theory and justified via their previous use in this field. I am not an expert in this but I would have liked to see further discussion of the limitations of this approach, including discussion of whether this 'dispassionate rationality' is still considered plausible (see e.g. Epstein & Chalen, 2006 [11])?

Reply:

Thank you for making this comment. The fundamental assumption of the behavior model within FIEM is optimization; individuals optimize their decisions each period, *conditional on the model specified by the researcher*. Thus, it is possible to specify a model that includes features that may better approximate the true decision making process individuals face. Examples could range from greater uncertainty about infection levels, individual health states, or state variable dynamics. Whether a researcher incorporates these features into a model depends on whether they are central to the research question, their preserve model tractability, and whether there is sufficient data variation to credibly model parameters that capture these forces. Note that "rationality" and "optimization" do not necessarily imply that individuals only use financial considerations in decision making, nor that two individuals will make the identical decision conditional on the observed state variables of themselves and the population. The utility function can be designed to include any type of cost or benefit that influences decision making. Moreover, in the model presented here, we assume there are unobserved factors influencing decision making, and given certain simplifying assumptions about the distribution of these factors in the population, decision making has the appearance of being probabilistic conditional on observed factors only. We have now added a paragraph in the Discussion section (see lines 464–476) to expand on these limitations that we have copied below.

Beyond rational expectations and economic trade-offs, FIEM can be extended to incorporate psychological and emotional dimensions of decision-making, capturing a broader sense of well-being than just health or financial. For example, emotional costs such as social isolation, fear, or stigma can be modeled as additional non-monetary terms in the utility function. Individuals who isolate at home may experience a utility penalty, representing loneliness or reduced social contact, which can be estimated using survey-based measures of well-being [1]. Similarly, cognitive biases, such as misperceptions of

infection risk, overconfidence, or time-inconsistent preferences, can be introduced by adjusting belief structures. For example, perceived infection risk could be modeled as a function of actual prevalence, incorporating bias parameters to reflect optimism or pessimism. These features are commonly explored in behavioral economics and psychology [2,3] and can be integrated into FIEM's decision model without disrupting its core structure. These examples highlight how FIEM can use dynamic optimization with additional choice frictions to better approximate the true decision making process and align with other research on the complex determinants of human behavior.

Reviewer Comment 3.5 — There is much discussion of model validation in the supplementary material, but this seems to focus on qualitative trends in model output with parameter changes? Were quantitative unit tests performed to test epidemiological and behavioural aspects of the model? E.g. when disabling waning immunity is the final size as you would expect for a population with one higher risk group?

Reply: Thank you for the comment. We indeed verified that when the behavioral feedback component of the model is disabled and the population consists of a single risk group, the model is equivalent to a conventional theoretical SIRS framework. We compute the analytical solutions for this single–risk–group scenario, including the basic reproduction number R_0, the initial growth rate r, and the values of the transmission rate required for an initial low level infection to rapidly increase vs go extinct (i.e., $R_0 < 1$). We confirmed that our simulation reproduced these analytical results across a range of parameter values (See revised SI Table 4).

Model	Transmission rate	Rate of recovery	Rate of waning	R_0	Peak size	Peak time
FIEM	0.30	0.14	0.0043	2.11	18.24%	45
Compartmental Model				2.14	18.18%	44
FIEM	0.30	0.14	0	2.12	17.88%	45
Compartmental Model				2.14	17.81%	43
FIEM	0.275	0.14	0	1.99	14.88%	50
Compartmental Model				1.96	14.76%	50
FIEM	0.25	0.14	0	1.99	11.15%	62
Compartmental Model				1.79	11.59%	58
FIEM	0.225	0.14	0	1.69	8.26%	68
Compartmental Model				1.61	8.32%	70
FIEM	0.2	0.14	0	1.46	4.90%	93
Compartmental Model				1.43	5.10%	90
FIEM	0.175	0.14	0	1.26	2.28%	129
Compartmental Model				1.25	2.23%	128
FIEM	0.15	0.14	0	1.08	0.38%	193
Compartmental Model				1.07	0.32%	202
FIEM Compartmental Model	0.125	0.14	0	< 1	–	–

Table 1: Comparison of FIEM (shotdown decision model) and Theoretical Compartmental Model under different parameter settings. FIEM is initialized with a single risk group to align with the assumptions of the theoretical compartmental model for direct comparison under varying parameter settings.

We also compared the timeseries of our model output to prior code developed in our group for other (non-behavioral-feedback) modeling applications to confirm agreement (See SI Figure 1). Those validations mentioned above are added to the revised SI page 16. For FEIM, which has multiple risk

groups and behavioral feedback in addition to the standard epidemiological model components, there is no analytic solution to compare to. Instead, the investigations shared in the Model Validation section of the Supplemental Methods were designed to qualitatively evaluate agreement between the model and the intuition of the various domain experts involved in the project. The more quantitative version of this investigation is the sensitivity analysis (Figures 4 and S6), where we varied each parameter individually (from 60% to 140% of its baseline) while keeping others fixed at their best-estimated value. This provided additional insight into the direction of each parameter's effect and its relative importance in shaping results such as epidemic size and labor participation.

Reviewer Comment 3.6 — The authors adopt an agent-based model, which by their nature are extremely flexible, and the authors emphasise this feature as an advantage of their modelling approach while exploring some of the computational limitations of including dynamic feedback in this approach. If the authors felt able, discussion of the computational feasibility of some of the suggested extensions, e.g. spatial heterogeneities, collective decision making, would be very interesting.

Reply: Thank you for this thoughtful suggestion. We agree that spatial heterogeneity and collective decision-making are promising directions for extending FIEM. Both are conceptually compatible with our framework. In terms of feasibility:

- **Spatial heterogeneity** could be incorporated by dividing the population into subpopulations (e.g., by region or community) and specifying between-subpopulation contact matrices. This would increase the state variables, as each subpopulation would have its own health dynamics and decision problem. From a computational standpoint, the cost scales with the number of risk groups (depends on state variables), since both the epidemic model and the behavioral Bellman equations must be solved separately for each group. While this increases complexity, especially for calibration, it is conceptually straightforward and technically feasible within our framework.
- **Collective decision-making** could be introduced by modeling social influence or coordination, such as peer effects or policy-driven compliance. However, incorporating such features would significantly increase computational complexity. Specifically, it would require solving a much larger number of unique decision problems through Bellman equations, potentially one for each distinct combination of social context and belief state. That said, incorporating collective decision-making remains feasible by employing techniques to reduce the dimensionality of the decision problem.

We believe both extensions are feasible, and we appreciate the opportunity to reflect on their integration into future versions of the framework.

Reviewer Comment 3.7 — While forecasting is deemed beyond the scope of this paper and noted as an area of future work, is it worth discussing the type of data that would allow this model to by verified/falsified, i.e. by expanding on the comments in line 397?

Reply: Thank you for the comment. We believe that the model we present here, and others like it, are much better suited for scenario planning than forecasting. Scenario planning, like that done by the COVID-19 Scenario Modeling Hub, aims to make longer term projections under a set of specific assumptions about control policies, population behavior, disease mechanisms, etc. This is an important direction for our future work, will require the model to be more carefully parameterized and validated for the population, disease, and intervention of interest.

As noted in our response to Comment 3.1 and Comment 3.3, data to parameterize individual decision-making model (i.e., risk perceptions, behavioral responses) and inform the stratification of the population by risk group are already available, from sources such as the COVID-19 Impact Survey in the U.S., and with the right infrastructure could be collected quickly during another emerging infectious disease outbreak. Ideally, model components, if not complete models, should be validated using retrospective data before being applied to real-time projections for emerging infections. From our extensive experience building scenario models using traditional epidemic modeling frameworks, we anticipate there will be many remaining challenges as we move this model from a proof-of-concept to a more applied setting and link to additional data. These include deciding on the degree of population stratification to include (for variables such as SES groups, vulnerability, disease severity), the complexity of the decision model, issues with data quality and compatibility such as representativeness and spatial and temporal resolution, and decision about which parameters will be estimated from independent data sources versus inferred in real-time during model calibration to target data (e.g., cases of disease). We are excited to begin to tackle these challenges in future work.

References

[1] D. Kahneman and A. Deaton, "High income improves evaluation of life but not emotional well-being," *Proceedings of the national academy of sciences*, vol. 107, no. 38, pp. 16 489–16 493, 2010.

[2] G. Loewenstein, "Emotions in economic theory and economic behavior," *American economic review*, vol. 90, no. 2, pp. 426–432, 2000.

[3] J. J. V. Bavel, K. Baicker, P. S. Boggio, V. Capraro, A. Cichocka, M. Cikara, M. J. Crockett, A. J. Crum, K. M. Douglas, J. N. Druckman *et al.*, "Using social and behavioural science to support covid-19 pandemic response," *Nature human behaviour*, vol. 4, no. 5, pp. 460–471, 2020.

[4] L. A. Robinson, M. R. Eber, and J. K. Hammitt, "Valuing COVID-19 Morbidity Risk Reductions," *Journal of Benefit-Cost Analysis*, vol. 13, no. 2, pp. 247–268, Jul. 2022. [Online]. Available: https://www.cambridge.org/core/journals/journal-of-benefit-cost-analysis/article/valuing-covid19-morbidity-risk-reductions/DA88757B384F9C9C70393974D8D8EC9F

[5] T. J. Kniesner and R. Sullivan, "The forgotten numbers: A closer look at COVID-19 non-fatal valuations," *Journal of Risk and Uncertainty*, vol. 61, no. 2, pp. 155–176, 2020, publisher: Springer. [Online]. Available: https://www.jstor.org/stable/45379043

[6] J.-T. Liu, J. K. Hammitt, J.-D. Wang, and M.-W. Tsou, "Valuation of the risk of SARS in Taiwan," *Health Economics*, vol. 14, no. 1, pp. 83–91, 2005, _eprint: https://onlinelibrary.wiley.com/doi/pdf/10.1002/hec.911. [Online]. Available: https://onlinelibrary.wiley.com/doi/abs/10.1002/hec.911

[7] D. Gyrd-Hansen, P. A. Halvorsen, and I. S. Kristiansen, "Willingness-to-pay for a statistical life in the times of a pandemic," *Health Economics*, vol. 17, no. 1, pp. 55–66, 2008, _eprint: https://onlinelibrary.wiley.com/doi/pdf/10.1002/hec.1236. [Online]. Available: https://onlinelibrary.wiley.com/doi/abs/10.1002/hec.1236

[8] B. Dhungel, M. S. Rahman, M. M. Rahman, A. K. Bhandari, P. M. Le, N. A. Biva, and S. Gilmour, "Reliability of early estimates of the basic reproduction number of covid-19: a systematic review and meta-analysis," *International journal of environmental research and public health*, vol. 19, no. 18, p. 11613, 2022.

[9] A. Wozniak, J. Willey, J. Benz, and N. Hart, "COVID Impact Survey: Version 1 [dataset]," Chicago, IL, 2020, dataset.

[10] S. L. Bacon, K. L. Lavoie, J. Boyle, J. Stojanovic, K. Joyal-Desmarais, and iCARE study team, "International assessment of the link between COVID-19 related attitudes, concerns and behaviours in relation to public health policies: optimising policy strategies to improve health, economic and quality of life outcomes (the iCARE Study)," *BMJ open*, vol. 11, no. 3, p. e046127, Mar. 2021.

[11] J. Epstein and J. Chelen, "Advancing agent_zero," *Complexity and evolution: toward a new synthesis for economics*, vol. 19, p. 299, 2016.
