# Supplementary material for: Improving policy design and epidemic response using integrated models of economic choice and disease dynamics with behavioral feedback
Source: PLoS Comput Biol. 2025 Oct 3;21(10):e1013549. doi: 10.1371/journal.pcbi.1013549 (PMC12510651; doi:10.1371/journal.pcbi.1013549)
Supplement: Attachment — Submitted filename: rep-a-ploscb-PCOMPBIOL-D-25-00647_R1.pdf [file pcbi.1013549.s004.pdf]

Comments on:

"Improving policy design and epidemic response using integrated models of economic choice and disease dynamics with behavioral feedback"

manuscript # PCOMPBIOL-D-25-00647_R1 re-submitted to *PLOS Computational Biology*

I just have a few comments.

1.) In my previous review I mentioned that FIEM could similarly be applied to chronic disease detection and progression, as individual behaviors at different points in time (i.e., screening, follow up, treatment, adherence) affect longevity. While I was encouraging it's use in those contexts, I was also advocating for citing some of that literature as efforts to incorporate the behavior in impacting outcomes exist. In fact, one of your parenthetical responses to reviewer #1 (namely that per-contact transmissibility is impossible to estimate directly) reminded me of the contribution in Gilleskie (1998) where illness contraction probability is difficult to estimate with traditional approaches (that may not incorporate behavior) because one doesn't observe (in most data) the sickness of individuals who do not seek treatment or miss work (the behaviors in that context). Yet, because the behavior is modeled explicitly (i.e., there may be some people who get sick who don't seek care and don't miss work), they can recover the illness contraction rate by estimation of the full model. You could cite some of these types of papers that have done something similar (albeit in an individual context) to what you are doing here.

2.) I appreciate the acknowledgement of my pickiness regarding the indicator d, but I still think the revised sentence is not technically correct:
"Utility (u(.)) is a function of observable-state variables summarized by z, a decision an individual could make denoted by d, state variables unobserved to the modeler but known to the individual ϵ and a vector of utility model parameters Θ. Each period, individuals choose among available alternative decisions to maximize the present discounted value of their lifetime flow utilities ... "

I'd suggest:
"Utility (u(.)) is a function of observable-state variables summarized by z, each available alternative in the individual's decisionmaking process is denoted by d, state variables unobserved to the modeler but known to the individual are in the vector ϵ and Θ is a vector of utility model parameters. Each period, individuals choose among available alternatives ~~decisions~~ to maximize the present discounted value of their lifetime flow utilities ... "

In other words, the "choosing among available alternatives to maximize…" IS the decision, or the act of deciding.

3.) I like the revised sentence *"Potential costs of working for susceptible individuals (currently uninfected) are the increased probability …. ",* but I'll admit that I got confused by "working for susceptible individuals". I was reading it as working for those people or with those people, as opposed "the costs for susceptible individuals of working are…". It might be worth thnking of a new way of ordering things to make sure the reader is not confused.

4.) I understand how assuming an infinite horizon helps you computationally, but I don't think it makes sense for individual decisionmaking, which is what you are modeling. Individual's die. And the length of that horizon matters when evaluating different alternatives, as you acknowledge. What is more difficult, I'd imagine, is figuring out how to incorporate a finite-horizon model of individual behavior in a "macro" model of the behavior of a society (lots of individuals who interact and are different ages and have different life expectancies for other reasons – your 8 different categories of agents). AND, then, evaluating societal policies. But, even though policymakers want to design policy that lasts, I believe it's still okay to have a model that isolates one such infectious event and how to handle it. It would allow you to report the tradeoff (in terms of welfare but also deaths) of different policies. Indeed, during our recent pandemic much of the discussion was about minimizing death (or illness) and not about welfare (lifetime utility).

It might be worth explaining why you adopted an infinite horizon to health-related people who would expect a finite horizon.

5.) I agree that adding in age is a logical extension, and perhaps a next step for the authors. This feature, however, makes adding in mortality more important, in my opinion.

6.) I'm not a fan of the statement: "Decisions re incomes, behavior, etc" in the new Figure 1 Decision Model square. Nowhere does your model have people making decisions about income, but rather to work or not, and you don't really have any other "behaviors". Perhaps you could replace it with something like: "Individual decisionmaking regarding behaviors (e.g., work)".
