# Supplementary material for: Improving policy design and epidemic response using integrated models of economic choice and disease dynamics with behavioral feedback
Source: PLoS Comput Biol. 2025 Oct 3;21(10):e1013549. doi: 10.1371/journal.pcbi.1013549 (PMC12510651; doi:10.1371/journal.pcbi.1013549)
Supplement: Attachment — Submitted filename: Epi_Econ_response_letter.pdf [file pcbi.1013549.s005.pdf]

Response to reviewer for manuscript #PCOMPBIOL-D-25-00647

We thank the editor and reviewers for their feedback in this second round of review. We have carefully addressed each comment and revised the manuscript accordingly. Below, we provide detailed, point-by-point responses describing the changes made. For the reviewers' convenience, we have highlighted significant changes in the revised manuscript in blue.

Response to the reviewers

Reviewer 1

Reviewer Comment 1.1 — The authors have done a great job at answering all reviewer comments. The only one that is not answered fully is comment 2.31 (Venerable was only corrected in one of the labels in the figure).

Reply: We thank the reviewer for carefully checking our revisions. We have now updated the type labels in both Figure 5 and Figure 8.

Reviewer 2

Reviewer Comment 2.1 — In my previous review I mentioned that FIEM could similarly be applied to chronic disease detection and progression, as individual behaviors at different points in time (i.e., screening, follow up, treatment, adherence) affect longevity. While I was encouraging it's use in those contexts, I was also advocating for citing some of that literature as efforts to incorporate the behavior in impacting outcomes exist. In fact, one of your parenthetical responses to reviewer 1 (namely that per-contact transmissibility is impossible to estimate directly) reminded me of the contribution in Gilleskie (1998) where illness contraction probability is difficult to estimate with traditional approaches (that may not incorporate behavior) because one doesn't observe (in most data) the sickness of individuals who do not seek treatment or miss work (the behaviors in that context). Yet, because the behavior is modeled explicitly (i.e., there may be some people who get sick who don't seek care and don't miss work), they can recover the illness contraction rate by estimation of the full model. You could cite some of these types of papers that have done something similar (albeit in an individual context) to what you are doing here.

Reply: We appreciate the reviewer's suggestion. We have now added a citation to Gilleskie (1998) to underscore how explicit modeling of individual behavior can recover otherwise unobservable disease dynamics. Please see below (also see revised manuscript page 3):

Nevertheless, there are notable examples where explicit behavioral modeling has been used to recover otherwise hidden health dynamics [1].

At the same time, we note that this manuscript is primarily focused on infectious disease applications, where our framework is most directly developed and validated. We view chronic disease modeling as a

promising direction for future extensions of FIEM, and we are grateful to the reviewer for emphasizing this broader applicability.

Reviewer Comment 2.2 — I appreciate the acknowledgement of my pickiness regarding the indicator d, but I still think the revised sentence is not technically correct:

> *"Utility ($u(\cdot)$) is a function of observable-state variables summarized by z, a decision an individual could make denoted by d, state variables unobserved to the modeler but known to the individual ϵ and a vector of utility model parameters Θ. Each period, individuals choose among available alternative decisions to maximize the present discounted value of their lifetime flow utilities ..."*

I'd suggest:

> *"Utility ($u(\cdot)$) is a function of observable-state variables summarized by z, each available alternative in the individual's decisionmaking process is denoted by d, state variables unobserved to the modeler but known to the individual are in the vector ϵ and Θ is a vector of utility model parameters. Each period, individuals choose among available alternatives ~~decisions~~ to maximize the present discounted value of their lifetime flow utilities ..."*

In other words, the "choosing among available alternatives to maximize..." *is* the decision, or the act of deciding.

Reply: Thank you for the suggested language. We have implemented in the revised draft.

Reviewer Comment 2.3 — I like the revised sentence *"Potential costs of working for susceptible individuals (currently uninfected) are the increased probability ..."*, but I'll admit that I got confused by "working for susceptible individuals". I was reading it as working for those people or with those people, as opposed to "the costs for susceptible individuals of working are...". It might be worth thinking of a new way of ordering things to make sure the reader is not confused.

Reply: Thank you for flagging that the sentence is unclear. We have replaced the text on pages 7–8 with the following, which we hope is easier for readers to follow.

With this formulation, the costs of working for a susceptible individual (currently uninfected) are the increased probability of future income loss and the disutility of becoming infected. The costs for an infected individual that chooses to work are the ongoing costs associated with disease symptoms or the stigma associated with being infectious.

Reviewer Comment 2.4 — I understand how assuming an infinite horizon helps you computationally, but I don't think it makes sense for individual decisionmaking, which is what you are modeling. Individuals die. And the length of that horizon matters when evaluating different alternatives, as you acknowledge. What is more difficult, I'd imagine, is figuring out how to incorporate a finite-horizon model of individual behavior in a "macro" model of the behavior of a society (lots of individuals who interact and are different ages and have different life expectancies for other reasons – your 8 different categories of agents). AND, then, evaluating societal policies. But, even though policymakers want to design policy that lasts, I believe it's still okay to have a model that isolates

one such infectious event and how to handle it. It would allow you to report the tradeoff (in terms of welfare but also deaths) of different policies. Indeed, during our recent pandemic much of the discussion was about minimizing death (or illness) and not about welfare (lifetime utility).

It might be worth explaining why you adopted an infinite horizon to health-related people who would expect a finite horizon.

Reply: Thank you for engaging with this assumption.

We primarily adopted the infinite-horizon for computational/numerical reasons. It is important to note that the infinite-horizon can capture mortality/death in a manner similar to a finite-horizon model. Both types of models can accommodate macroeconomic factors and heterogeneous populations. The comparative advantage of finite-horizon models is for answering questions about dynamic decisions where there is an obvious end point (e.g., optimal labor supply and savings up until retirement). We should note that these are sometimes interchanged in economics since the discount factors means results do not change if we allow finite horizon models with long horizons and infinite horizon models.

We agree that including mortality is an important extension and would enrich the policy evaluation and tradeoff discussion. As with the other extensions mentioned in the paper, FIEM can be modified to capture mortality as a factor in the decision making process. We see this as an important area for future work. The objective of this paper was to serve as a proof of concept for how to integrate dynamic discrete choice and epidemiological disease spread in a rigorous and credible manner. For the current paper, mortality is captured as part of the cost of illness (θ_x) that includes avoidance of illness. We did this to keep the model as simple as possible and still make the point that we can capture economic behavior and epidemiological disease spread in a single framework.

Reviewer Comment 2.5 — I agree that adding in age is a logical extension, and perhaps a next step for the authors. This feature, however, makes adding in mortality more important, in my opinion.

Reply: We agree adding mortality is a natural extension for FIEM. Our prior response illustrates that this can be accommodated in a model with an infinite horizon.

Reviewer Comment 2.6 — I'm not a fan of the statement: "Decisions re incomes, behavior, etc" in the new Figure 1 Decision Model square. Nowhere does your model have people making decisions about income, but rather to work or not, and you don't really have any other "behaviors". Perhaps you could replace it with something like: "Individual decisionmaking regarding behaviors (e.g., work)".

Reply:

We have revised figure as reviewer suggested:

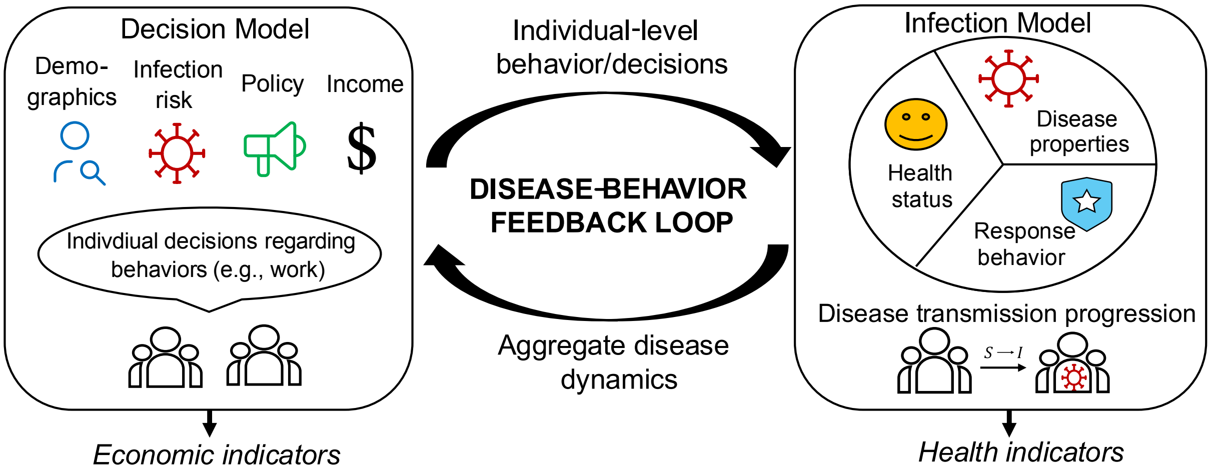
Decision Model
Demo-
graphics
Infection
risk
Policy
Income
$
Indivdiual decisions regarding
behaviors (e.g., work)
Economic indicators
Individual-level
behavior/decisions
DISEASE-BEHAVIOR
FEEDBACK LOOP
Aggregate disease
dynamics
Infection Model
Disease
properties
Health
status
Response
behavior
Disease transmission progression
S → I
Health indicators

References

[1] D. B. Gilleskie, "A dynamic stochastic model of medical care use and work absence," *Econometrica*, pp. 1–45, 1998.
